# Ethics of AI in healthcare: a scoping review demonstrating applicability of a foundational framework

**DOI:** 10.3389/fdgth.2025.1662642

**Published:** 2025-09-10

**Authors:** Aaron J. Gorelik, Mengyuan Li, Jessica Hahne, Junyi Wang, Yongqi Ren, Lei Yang, Xin Zhang, Xing Liu, Xiaomin Wang, Ryan Bogdan, Brian D. Carpenter

**Affiliations:** ^1^ Department of Psychological & Brain Sciences, Washington University in St. Louis, St. Louis, MO, United States; ^2^ School of Humanities, Central South University, Changsha, China; ^3^ Xiangya Hospital, Central South University, Changsha, China; ^4^ Center for Clinical Pharmacology, Third Xiangya Hospital, Central South University, Changsha, China

**Keywords:** ethics, Artificial Intelligence, Responsible AI, healthcare, Beneficence, Non-Maleficence, Autonomy, Justice

## Abstract

Artificial Intelligence (AI) is increasingly being adopted across many industries including healthcare. This has brought forth the development of many new independent ethical frameworks for responsible use of AI within institutions and companies. Risks associated with the application of AI in healthcare have high stakes for patients. Further, the existence of multiple frameworks may exacerbate these risks due to potential differences in interpretation and prioritization in said frameworks. Resolving these risks requires an ethical framework that is both broadly adopted in healthcare settings and applicable to AI. Here, we examined whether a framework consisting of the 4 well-established principles of biomedical ethics (i.e., Beneficence, Non-Maleficence, Respect for Autonomy, and Justice) can serve as a foundation for an ethical framework for AI in healthcare. To this end, we conducted a scoping review of 227 peer-reviewed papers using semi-inductive thematic analyses to categorize patient-related ethical issues in healthcare AI under these 4 principles of biomedical ethics. We found that these principles, which are already widely adopted in healthcare settings, were comprehensively and internationally applicable to ethical considerations concerning use of AI in healthcare. The existing four principles of biomedical ethics can provide a foundational ethical framework for applying AI in healthcare, grounding other Responsible AI frameworks, and can act as a basis for AI governance and policy in healthcare.

## 1 Introduction

The potential of Artificial Intelligence (AI) to improve healthcare (e.g., automate tasks, improve diagnoses and treatment) has led to its widespread adoption. In a 2022 international systematic review, AI usage by medical students and physicians was reported to be between 10% and 30% (1). By 2023, a separate survey found that nearly 38% of physicians in America were already using AI in practice, with nearly two-thirds stating they recognized its potential advantages (2). However, this rapid integration of AI also introduces novel ethical risks (e.g., explainability) across healthcare research and clinical settings (3–5).

In 2022, 75% of Americans believed healthcare providers would adopt AI too quickly before fully understanding risks for patients (6). Concerns among the public are founded on more than simple technological aversion. Indeed, ethical issues and unknowns raised by involvement of AI in healthcare are evolving as quickly as the technology itself, while regulatory governance currently lags behind (e.g., explicit permission to use patient data for training AI models) (7–9). To address these challenges, a new area of study referred to as “Responsible AI” (RAI) has emerged, defined as “the practice of developing, using, and deploying [AI] systems in a way that is ethical, transparent, and accountable” (10–13).

To date, numerous frameworks for RAI have been suggested by both governmental (14–20) and non-governmental organizations (e.g., technology companies) (21–26) (see Tables 1, 2 for common areas of concern in RAI and how they correspond to healthcare issues). However, these frameworks feature noticeable limitations which are especially relevant in healthcare, where use of AI has significant implications for patients (27). Such limitations include but are not limited to the abstract nature of these frameworks (28). For example, several frameworks include the concept of “fairness”. However, fairness in AI and fairness in healthcare do not always align. Fairness may variously describe: the impact of class imbalances in AI model training data (29), poor generalizability of AI models (30), expected patient outcomes from AI-produced recommendations (31), and/or focus on inappropriate metrics (e.g., focusing on accuracy over clinical utility) (32). Further, emerging RAI frameworks vary widely in content, such that no one framework could necessarily claim to be comprehensive. Indeed, the very nature of having a variety of frameworks to choose from may lead to a focus on compliance for the sake of compliance (33). Finally, beyond general RAI principles, specific ethical considerations for healthcare need to be considered (34, 35). As adoption of healthcare AI continues to accelerate, there is a need for RAI frameworks for healthcare to be grounded in foundational principles that are already widely accepted and applied in healthcare around the world.

**Table 1:** Definitions of areas of concern in Responsible AI (10–13, 106).

Areas of concern	Definitions
Privacy	Ensuring that individuals' personal data are collected, processed, and stored in ways that respect their rights, protect their identity, and maintain confidentiality.
Security	The practice of safeguarding AI systems and the data they use from unauthorized access, breaches, and malicious attacks to ensure system integrity and reliability.
Transparency	The presence of clear, accessible, and understandable explanations of how AI systems operate, make decisions, and process data.
Inclusivity/Fairness	Ensures that AI systems are designed and deployed in a manner that avoids bias, promotes equitable outcomes, and considers the needs and perspectives of diverse populations.
Governance	Involves the establishment of policies, frameworks, and processes to guide the responsible development, deployment, and use of AI technologies.
Accountability	Requires that individuals involved in creating and deploying AI systems take responsibility for their outcomes and ensure mechanisms are in place to address errors, misuse, or harm.
Reliability	Focuses on the consistent and dependable performance of AI systems, ensuring they function as intended across diverse scenarios and over time.
Social/Environmental Well-being	Emphasizes the development and operation of AI technologies that does not harm society and minimize environmental impact and support long-term ecological balance.

**Table 2:** Examples of ethical concerns about AI in healthcare and their corresponding Responsible AI principles.

Examples of ethical concerns about AI in healthcare	Corresponding principles of Responsible AI
More extensive informed consent is needed from patients concerning third-party access to their information due to concerns about how their data will be used (8).	Governance and Privacy
Integration of AI into electronic health records or use of AI for health-related communication elevate the risk of data breaches (8).	Security and Privacy
Training and fine-tuning of AI models on patient data may pose risks for inadvertent replication and dissemination of sensitive information (36, 107, 108).	Privacy
There has been a growing precedent of companies using and selling individual user data to other companies to train models, often without regulatory oversight and accountability (109).	Privacy and Governance
Training and fine-tuning of AI models on patient data may pose risks for the exacerbation of existing demographic biases and inequities in healthcare (36, 107).	Inclusivity/fairness and Lack of Transparency
Questions remain as to how much human actors should be held accountable for harm done to patients when clinical decisions are influenced by AI-generated errors. Who are responsible: individuals who curate the training data, individuals training the model, or individuals applying the model? (84, 110)	Accountability, Governance, and Lack of Transparency
Large language models are complex systems which are made up of many components (including both software and hardware) which may fail. Medical/lifesaving equipment typically has much higher failure tolerances given the severe consequences, however, there is no agreed upon standard for the reliability of AI in healthcare (111).	Reliability
Training large-scale AI models requires significant energy, contributing to environmental degradation through increased carbon emissions, which, over time, can indirectly impact patient health because of global warming (112).	Social/Environmental Well-being

A recent literature review by Ong and colleagues (36) proposed the four principles of biomedical ethics by Beauchamp and Childress (37, 38) as an ideal standard to guide use of large language models in medicine. In doing so, the authors called upon a classical health ethics framework with a well-established history across cultures, time, and technology as a potential unifying framework for ethical evaluation of AI in healthcare. Beauchamp and Childress's framework emerged in 1979 as part of an international wave of biomedical ethics reform and is used to this day (37). The framework encompasses the following four principles: Beneficence (to take positive action to enhance the welfare of patients and to minimize potential for harm); Non-Maleficence (not to inflict harm on patients); Respect for Autonomy (to respect the capacities of patients to hold their own views, make choices, and act based on their values and beliefs); and Justice (to distribute healthcare benefits appropriately and fairly).

Although some authors have speculated about the utility of these principles as a foundational framework for assessing ethical implications of AI in healthcare (36, 39), there has been limited effort to systematically examine how research and commentary in current peer-reviewed literature on AI in healthcare maps onto these four well established biomedical principles. In this scoping review, we aim to map ethical discussion from current peer-reviewed literature about AI in healthcare onto the four principles, to demonstrate their utility as a concise, foundation for RAI in healthcare that has already been internationally adopted in healthcare settings. We demonstrate this by using said principles to identify, categorize, and examine emerging patient-related ethical implications of AI in healthcare. Finally, we discuss how leveraging these well-established principles as a foundational framework can help expedite regulatory governance of RAI in healthcare.

## 2 Materials and methods

Our scoping review was conducted using criteria outlined by Mak and Thomas (40) and the Preferred Reporting Items for Systematic Reviews and Meta-Analyses extension for Scoping Reviews (PRISMA-ScR) guidelines (see Supplementary Appendix 1) (41).

### 2.1 Search strategy

PubMed and EMBASE databases were searched for articles on the use of AI in healthcare. The search strategy and rationale as well as search terms are provided in Supplementary Appendix 2. The final search, which included any articles published from database inception to February 17, 2024, after removal of duplicates, yielded 10,107 articles for abstract screening.

### 2.2 Eligibility criteria

Studies included in our review were required to be published or in press in a peer-reviewed journal or conference proceeding in English or have an accompanying English translation and mention AI in healthcare settings in the context of human input or oversight. Broadly, we conceptualized AI in healthcare as a partner to human activity and oversight rather than a sole agent operating independently. In accordance with the qualitative nature of our research question, articles were not required to be empirical in nature; editorials, reviews, and conference proceedings with content more substantial than single-paragraph abstracts were included in the review. To focus our findings on direct ethical implications for patients, we excluded articles that focused only on AI involvement in research paper writing and publishing, clinician education and training, or research that did not involve human participants.

Finally, articles were only selected if they substantively referenced the ethical implications of healthcare AI with respect to patients. To be considered “substantive,” ethical content in abstracts had to either use a broad, inclusive term such as “ethics” or “ethical implications” or at least two phrases referencing ethical issues relating to patients such as “improving patient care,” “data privacy,” “bias,” “informed consent,” “health equity,” “transparency of data use,” or “[sociodemographic] representativeness of training data”.

### 2.3 Screening, data extraction, and synthesis

The full text of articles passing title and abstract screening [conducted by authors JH and AG using Rayyan (42)] was reviewed by a team of 4 coders (authors ML, JW, YR, LY) who extracted and synthesized relevant data. A study eligibility guide based on the Population/Concept/Context framework (43), was developed to train reviewers and referred to in both title/abstract screening and full-text review (Supplementary Appendix 3).

#### 2.3.1 Study eligibility: reviewer calibration and evaluation of consistency

During an initial training phase, a batch of 900 (roughly 9% of total) abstracts were screened by JH and AG independently during initial training to resolve differences through consensus and iteratively refine study eligibility criteria applied to various types of content covered by abstracts. To achieve calibration between the two reviewers following training, a second batch of 870 (roughly 9% of total) abstracts were screened independently by both reviewers, and 93% agreement was achieved, exceeding the minimum of 90% agreement recommended by Mak and Thomas (40). Differences across both batches of 900 and 870 articles respectively were resolved through consensus, and the remainder of abstracts were divided between the two calibrated reviewers until completion of title-and-abstract screening.

Following abstract screening, authors ML and JW underwent an initial training phase for full-text screening, during which both authors independently conducted screening, data extraction, and data synthesis for a preliminary batch of 40 articles (approximately 11% of total articles included after abstract screening). Any differences were resolved by consensus. Authors YR and LY then completed screening, extraction, and synthesis for a second batch of 40 (roughly 11% of total) articles under independent individual supervision by authors ML and JW, and any differences were again resolved by consensus. For each batch of 40 through the remainder of articles, each of the four reviewers completed screening, extraction, and synthesis for 10 articles. To ensure ongoing calibration, a random number generator was used to select one article from each batch of 40 that was then independently reviewed by all four authors. Any differences in the randomly selected calibration articles were resolved through ongoing consensus. Finally, all synthesized data were reviewed by author ML for consistency. Articles were excluded after data extraction and full-text screening if they did not meet eligibility criteria.

#### 2.3.2 Data extraction

Data extraction involved reading each article in full and charting key summary information, including citation information; country of origin; article aims; study population and intervention (if applicable); findings; and ethical considerations discussed (see Supplementary Appendix 4 for details).

#### 2.3.3 Thematic analysis

Data from all articles included after full-text screening was synthesized using semi-inductive thematic analysis in NVivo 12 for analysis of mixed methods research (44). This approach was informed by Fereday and Muir-Cochrane (45), who described thematic analysis that uses a hybrid approach between deductively applying a predetermined framework to data and inductively allowing for additional information to emerge from the data in the process of analysis (46). We used this approach due to our aim to discover emerging ethical implications of AI and examine their relevance to the four principles.

In the process of thematic analysis, we followed the six stages outlined by Fereday and Muir-Cochrane (45). In Stage 1, we developed an initial codebook of four codes, using the principles of biomedical ethics as a deductive theoretical framework to guide our analysis. We established definitions for each of these four main codes based on their definitions from Beauchamp and Childress (37). In Stage 2, we tested the applicability of codes to the data by comparing the application of these four main codes during the aforementioned training phase in which authors ML and JW both coded data from the same batch of 40 articles. In Stage 3, we used the process of data extraction to summarize the data and identify sub-codes that represented emerging ethical implications for patients related to healthcare AI. Sub-codes were defined, discussed, and revised through iterative discussion with the whole research team. In Stage 4, once a final codebook was established, the codes were applied to all articles in the review with the intention of identifying meaningful units of text for the synthesis of themes (see Table 3 for a comprehensive list of all codes included in the final codebook, grouped by theme, with representative coded excerpts). In Stage 5, once all articles were coded, excerpts representing each code were compared and connected to synthesize themes. In Stage 6, themes were scrutinized to ensure that they were accurate summaries of the coded ethical implications that emerged from the data, resulting in our final narrative summary of the results. Finally, as a *post-hoc* analysis, once all themes were generated, they were then mapped to the RAI principles identified in Table 1.

**Table 3:** Main findings organized by ethical principles, Responsible AI principles, themes, and sub-codes with representative excerpts.

Ethical Principle	
Theme	
Representative Responsible AI (RAI) Principles	
Representative Excerpt	
Specific Ethical Issue (Sub-Code)	Articles in Review with Sub-Code Applied *n* (*n*/227)
Beneficence	
Theme 1: Health service quality: accuracy, efficiency, and safety	
RAI Principles: Reliability, Social/Environmental Well-being	
“AI algorithms can also add value by acting as a ’second reader’ [of radiologic images], more broadly […] enhancing the quality of care and safety” (113).	
Accuracy and efficiency of care	75 (33·0%)
Promotion of well-being	52 (22·9%)
Personalized medicine	40 (17·6%)
Clinical validity of algorithms	32 (14·1%)
Quality and safety of care	26 (11·5%)
Theme 2: Patient experience and clinician-patient relationship	
RAI Principles: NA	
“Traditionally, medicine is based on a relationship between a patient and their healthcare providers. AI will add a third component […] which may support or complicate the relationship” (54).	
Clinician-patient relationship/communication	41 (18·1%)
Patient-centered care	17 (7·5%)
Theme 3: Social and humanistic dimensions of health services	
RAI Principles: NA	
“First, AI is a technology based on algorithms and data, unable to experience emotions or demonstrate empathy” (56).	
Dignity, empathy, and humanism in healthcare	16 (7·0%)
Emotional support	10 (4·4%)
Non-Maleficence	
Theme 1: Data quality: accuracy, reliability and generalizability	
RAI Principles: Inclusivity/Fairness, Governance	
“Where medical data are unstructured, lack uniformity and standardization annotation there is potential for such data to directly affect the quality of medical AI algorithm models” (114).	
Low data quality, accuracy/reliability concerns	33 (14·5%)
Insufficient data volume, generalizability concerns	15 (6·6%)
Theme 2: Patient privacy and data protection	
RAI Principles: Privacy, Security, Governance	
“Privacy and security are also significant issues, as ChatGPT collects data during training, including potentially sensitive personal information, and user interactions with the system may inadvertently disclose personal details, posing risks if obtained by malicious entities” (61).	
Privacy and data protection	107 (47·1%)
Theme 3: Other technology risks and regulatory issues	
RAI Principles: Governance, Inclusivity/Fairness	
“From these examples, it is worrisome to learn that chatbots can generate fabricated and incorrect information, or what is known as ‘artificial hallucination’” (115).	
Risk/error management	30 (13·2%)
Need for human regulation/oversight	25 (11·0%)
Trust in implementing AI for healthcare	19 (8·4%)
Misinformation	17 (7·5%)
Misuse	10 (4·4%)
Technology addiction; algorithmic overreliance	5 (2·2%)
Respect for Autonomy	
Theme 1: Patients' right to informed consent	
RAI Principles: Transparency, Governance	
“Individuals interacting with ChatGPT may not always be aware of the fact that they are communicating with an AI system, particularly when incorporated into chat interfaces or client assistance platforms” (47).	
Informed consent	37 (16·3%)
Patients' health-related knowledge	6 (2·6%)
Theme 2: Transparency or understandability	
RAI Principles: Transparency	
“The ‘black-box' nature of NLP (natural language processing)/ML (machine learning) systems poses challenges to human agency, from the perspective of researchers, clinicians, and patients alike” (62).	
Transparency or understandability	93 (41·0%)
Theme 3: Shared decision-making in healthcare	
RAI Principles: Social/Environmental Well-being	
“These illustrate the full range of areas where AI can have an impact: from apps that help patients manage their care themselves, to online symptom checkers and e-triage AI tools, to virtual agents that can carry out tasks in hospitals, to a bionic pancreas to help patients with diabetes” (116).	
Autonomy (without direct mention of the ethical principle of “Respect for Autonomy”)	18 (7·9%)
Shared decision-making	17 (7·5%)
Justice	
Theme 1: Responsibility and accountability for health service quality	
RAI Principles: Accountability, Social/Environmental Well-being	
“Similarly, if an AI algorithm makes a mistake, who is liable, the anesthesiologist or the device maker? These questions will need to be addressed to allow for change to occur” (117).	
Clear assumption of responsibility; accountability	45 (19·8%)
Theme 2: Affordability and accessibility of health services	
RAI Principles: Inclusivity/Fairness, Social/Environmental Well-being	
“Through this synergy, patients could access healthcare services from the comfort of their homes […]. This could significantly improve healthcare accessibility and convenience, particularly for patients in rural or underserved areas” (88).	
Accessibility of services	13 (5·7%)
Affordability/availability of services	10 (4·4%)
Theme 3: Diversity, equity, and inclusion in the context of health	
“This will mitigate a known limitation of AI models, which are often biased due to the underrepresentation of populations such as those individuals with intellectual disabilities” (81).	
RAI Principles: Inclusivity/Fairness	
Bias, discrimination	80 (35·2%)
Equity, fairness	54 (23·8%)
Cultural diversity, sensitivity, and inclusivity	13 (5·7%)

## 3 Results

**Figure 1:**
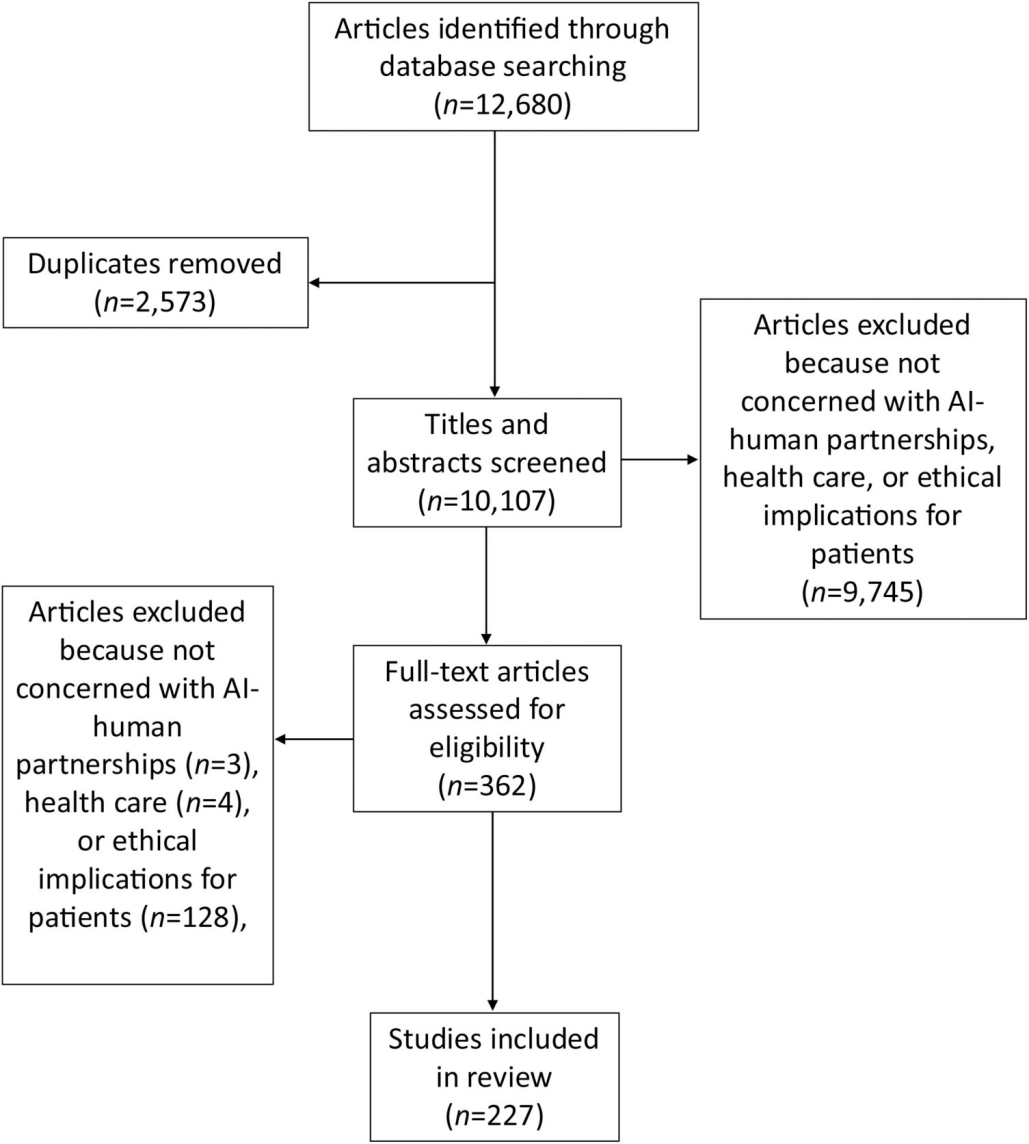
PRISMA flow diagram.

**Figure 2:**
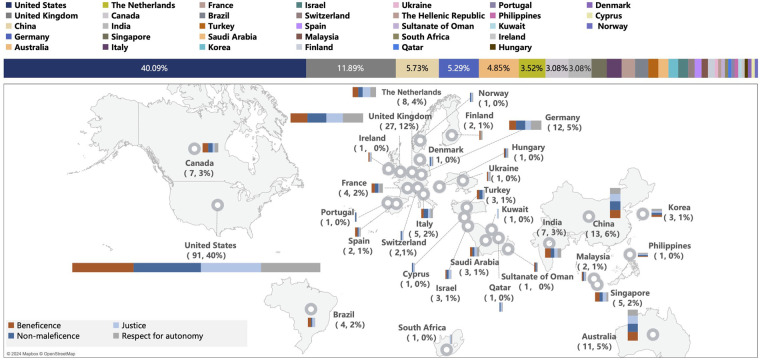
Articles included in review and discussion of issues related to the four principles, by country. Map data from Mapbox and OpenStreetMap.

After abstract and full-text screening of all 10,107 unique articles, 227 articles (2.25%) met criteria for inclusion (Figure 1; Supplementary Appendix 4). The majority of included articles were editorials (*n* = 96; 42%), review articles (*n* = 73; 32%), or empirical articles (*n* = 46; 20%) with the remaining (*n* = 12, 5%) being composed of other article types (e.g., workshop reports, case studies, etc.) (see Supplementary Appendix 5 for details). Publication dates of articles were concentrated between 2020 and 2024 (*n* = 203/227; full date range: 2007–2024). Articles originated across 33 countries (first author's country of affiliation), with the vast majority (*n* = 91; 40%) coming from the US (Figure 2).

In Supplementary Appendix 6, we present a non-exhaustive list of example phrases that were considered representative of each principle during abstract screening, highlighting the relevance of each principle to ethical issues in healthcare AI. Semi-inductive thematic analysis revealed that researchers' discussion of ethical considerations of AI use in healthcare spanned across the four principles. Numbers of articles discussing topics related to each of the four principles were quite comparable, including: Beneficence (*n* = 136), Non-maleficence (*n* = 148), Respect for Autonomy (*n* = 130), and Justice (*n* = 136). Word clouds showing further findings on words mentioned at the highest frequency within coded text for each principle are presented in Supplementary Appendix 7.

A total of 29 more specific ethical issues were identified and treated as sub-codes to the four principles in our analysis. Issues discussed by the highest number of articles included “privacy and data protection” (sub-code to Non-Maleficence; *n* = 107), “transparency and understandability” (sub-code to Respect for Autonomy; *n* = 93), “bias and discrimination” (sub-code to Justice; *n* = 80), “accuracy and efficiency” (sub-code to Beneficence; *n* = 75), and “equity and fairness” (sub-code to Justice; *n* = 54). Findings on the four principles and 29 sub-codes were consolidated into themes, summarized below. A list of all themes and sub-codes pertaining to each principle are included in Table 3.

### 3.1 Narrative summary: relevance of the principle of beneficence

#### 3.1.1 Theme 1: health service quality: accuracy, efficiency, and safety

Articles in our review stated that AI holds promise for addressing human errors and managing escalating healthcare workloads (47–49). It can also be utilized to promote more personalized treatment (50) by tailoring treatment methods based on the specific characteristics and risk status of each patient (51, 52). However, some articles in our review questioned the accuracy and efficiency of AI chatbots, with potentially harmful consequences for patients (53).

#### 3.1.2 Theme 2: patient experience and doctor-patient relationship

The presence of AI will introduce a third party with a shared role in diagnosis, management, and prediction of disease. Some articles suggested that this third party may complicate the traditional healthcare relationship (54) or reduce face-to-face communication, while other articles highlighted how AI can generate comprehensive summaries of patients' medical information and handle other documentation, thus allowing clinicians to spend more time with their patients (52).

#### 3.1.3 Theme 3: social and humanistic dimensions of health services

Several articles in our review emphasized the possibility of incorporating empathy, patient values, and patient preferences into AI-assisted care delivery and decision-making (55). Others suggested that because AI functions based on algorithms, it cannot truly show empathy of the same nature as human providers (56). Particularly in mental healthcare, some articles argued that AI may exacerbate social isolation by inadequately substituting human connection (57, 58).

### 3.2 Relevance of the principle of non-maleficence

#### 3.2.1 Theme 1: data quality: accuracy, reliability and generalizability

The hazards and risks of AI can be influenced by a variety of factors, including data quality. For example, one article noted that during COVID-19, unreliable data and algorithms led to inaccurate outbreak tracking and predictions (59). Other examples in radiology demonstrated that AI accuracy is influenced by the quality of the data (60).

#### 3.2.2 Theme 2: patient privacy and data protection

Healthcare AI, especially chatbots, collect and use sensitive patient data, posing risks for data leaks and in turn compromising patient security (61, 62). Additionally, the healthcare sector faces higher AI related cyber-attack rates than other industries, with AI systems being exploited to expose vulnerabilities (63). While privacy is a fundamental right, it must often be balanced with public health benefits, such as improved care and research. Notably, large datasets and AI make re-identifying de-identified individuals easier, intensifying ethical concerns (64). Experts call for stricter data regulations, stakeholder education on secure practices, and tighter oversight of AI developers and data users (65–67).

#### 3.2.3 Theme 3: other technology risks and regulatory issues

There is a risk of human overreliance on AI in medicine, with clinicians experiencing “alert fatigue” from excessive information input, such as automated notifications (63). Overuse can harm users' mental health, foster technology addiction, and reduce trust in healthcare (68–70). It was also noted that currently, there is a dearth of regulation concerning many AI models, such as Multimodal Large Language Models, raising safety and ethical concerns (71). There was a consistent message across articles that stronger regulatory frameworks are needed, but developing them requires navigating technical, legal, and ethical challenges (72–74).

### 3.3 Relevance of the principle of respect for autonomy

#### 3.3.1 Theme 1: patients' right to informed consent

Informed consent in AI-assisted healthcare ensures patients know how their data is used (75). For true informed consent, clinicians, developers, and researchers must explain AI's roles, limitations, and data use implications (62, 76). Current consent procedures are often either overly simple or overly complex, with lengthy terms of service (57). Given the ubiquity of AI integration, patients may unknowingly interact with AI without clarity on data storage and usage (47). Dynamic consent processes are needed, allowing patients to manage and withdraw data sharing at any time (75, 77, 78).

#### 3.3.2 Theme 2: transparency or understandability

The “black-box” nature of algorithms can erode trust when decisions cannot be validated or security risks arise (79). Many articles argued that improving algorithm traceability, reducing bias, and making AI decisions understandable are essential to fostering trust among clinicians and patients (61, 80, 81).

#### 3.3.3 Theme 3: shared decision-making in healthcare

Shared decision-making involves collaboration between clinicians and patients, balancing information, risks, and preferences. AI facilitates this process by analyzing data to identify risks and assist decisions (82). AI-assisted algorithms can aid medical decisions, using large amounts of health data (83). But while AI can leverage big data to guide decisions, it is currently unclear how AI-derived suggestions can be applied more flexibly to account for the dynamic nature of human autonomy and decision-making.

### 3.4 Relevance of the principle of justice

#### 3.4.1 Theme 1: responsibility and accountability for health service quality

Articles highlighted how AI in healthcare complicates accountability for service quality. Responsibility lies with developers, clinicians, and agencies, requiring clear governance frameworks. Some articles posited that clinicians must always make final decisions, as they are directly accountable, but noted that shared human-AI actions create an “accountability gap,” especially with “black-box” systems (84). Current ethical and legal norms inadequately address responsibility for failures (85). Proposed strategies include clearer guidelines, defining AI's role, and ensuring clinicians retain ultimate responsibility (86). However, health-related AI regulations are still evolving, leaving **gaps in accountability for errors** (87).

#### 3.4.2 Theme 2: affordability and accessibility of health services

Healthcare AI could significantly improve the accessibility and convenience of medical services by providing remote diagnosis, intelligent assisted diagnosis and treatment, and other new means of healthcare that could enable more patients to receive timely and effective assistance; particularly patients in rural or underserved areas (53, 88). During the COVID-19 outbreak, AI-based services offered patients alternatives to face-to-face visits, saving time, transportation costs, and infection risks (89).

#### 3.4.3 Theme 3: diversity, equity, and inclusion in the context of health

AI bias and discrimination were noted as major concerns, with LLM responses reflecting societal biases in training data, amplifying disparities in healthcare (61, 90). Bias often affects minoritized groups due to underrepresentation in training datasets (81). Addressing this requires more diverse, representative data. Some articles also explored AI's emerging role in equitably allocating scarce public health resources (91, 92).

## 4 Discussion

The rapid expansion of AI in healthcare has led to the development of numerous, sometimes overlapping or contradictory frameworks (14–26). In this scoping review of 227 articles, we found that the four well-established principles of biomedical ethics (Beneficence, Non-Maleficence, Respect for Autonomy, and Justice) can provide a useful foundational framework for ethical application of AI in healthcare. Our analysis demonstrated that all ethical considerations of the 227 articles mapped onto one or more of the four ethical principles, and consequently their derived themes (see Table 3). Additionally, many of the identified themes corresponded to one or more concerns highlighted by the general principles from Responsible AI (RAI) (Table 1), such as security, inclusivity/fairness, governance, accountability, social/environmental well-being, and transparency (see Table 3 and Narrative Summary in Supplementary Appendix 8 for details). However, in the thematic analysis, two of the themes identified for the principle of Beneficence did not map onto these general RAI principles but are common to healthcare (i.e., 1) patient experience and clinician-patient relationship and 2) social and humanistic dimensions of health services). Collectively, our findings suggest that the four widely accepted principles of biomedical ethics provide a relevant foundation to build upon for ethical evaluation of AI in healthcare and could be used to ground interpretation and prioritization of other RAI frameworks for specific uses in healthcare. While various newer RAI frameworks may offer more specific ethics-related language relevant to applying AI to modern healthcare (93), our findings suggest that the four bio-medical ethical principles can ground existing RAI frameworks by helping to bridge, clarify, and prioritize existing RAI principles while also offering a “safety net” to ensure that foundational healthcare-specific ethical concerns are addressed (94).

Using a classical, widely accepted ethical framework like the four principles presents an opportunity for government bodies and regulatory agencies to establish more straightforward, consistent, and streamlined guidelines for the governance of AI in healthcare. As can be observed by the international spread of articles (33 countries) that met inclusion criteria for our review (see Figure 2), the ethical issues surrounding application of AI in healthcare are a subject of global discourse. This approach allows for evolution of existing policies based on these principles which have both withstood the test of time and have already shaped existing policies in biomedicine. Thus, rather than the creation of completely new guidelines for regulation of AI in healthcare, government and regulatory agencies can instead focus on building upon these foundational biomedical ethical principles and applying them to the current technological moment. In addition to simplifying development of regulations, this would promote universally recognized ethical standards for integration of AI technologies in the healthcare sector. Enabling broad coverage, in turn, will allow for current and future RAI frameworks in healthcare to be as prescriptive as needed and to evolve with technology while maintaining the same foundational four bio-medical ethical principles.

When mapping the literature on AI in healthcare onto the four principles, we found that, similarly to how various areas of RAI overlap (see Table 2), multiple ethical principles are often simultaneously implicated in addressing AI-related issues in healthcare. We believe in some cases this highlights the complementary nature of the four principles in examining different sides of an issue, while in others, it may highlight ethical dilemmas involving tensions between two or more principles. Still other cases may call for prioritizing application of one or more principles over the others.

For example, ethical issues related to use of patient data in AI can be understood to involve all four principles in a manner that encourages examination of various sides of data-related issues (52, 95). When AI models are handling vast amounts of sensitive patient information, ensuring data security becomes paramount (96), and the principle of Non-Maleficence is implicated in the potential for data breaches, as sensitive health information leaks may lead to personal privacy damage, information abuse, identity theft, and other harms (97, 98). In terms of the principle of Respect for Autonomy, data sharing in AI training and analysis raises need for new forms of informed consent (75, 99). Third, the principle of Beneficence underscores the positive potential to use data to improve healthcare and patient outcomes, as well as the need for benefits to individuals and society to outweigh the risks. Lastly, the principle of Justice is invoked when ensuring that data collection and use do not disproportionately burden or benefit particular groups (56, 100).

In other cases, different principles may conflict, raising ethical dilemmas. Rather than nullifying the applicability of the four principles, such cases highlight that the original intention of the four principles was not to stand alone as a comprehensive moral theory but rather to provide a normative framework as a starting point for ethical practice. In addition, the long history of the four principles offers established methods for resolving ethical conflicts and dilemmas, such as the rule of double effect (RDE). The RDE is invoked to justify claims that a single act, which has one or more good effects and one or more harmful effects (such as death), is not always morally prohibited (101). Classical formulations of the RDE identify four conditions or elements (the nature of the act, the agent's intention, the distinction between means and effects, and proportionality between the good effect and the bad effect) that must be satisfied for an act with a double effect to be justified (102). The RDE may serve as a helpful framework when the need arises to judge single instances of apparent contradictions between the potential benefits of applying AI to healthcare (such as improved efficiency and quality of medical services) and its potential negative effects (such as risks of harmful errors and data breaches).

Finally, in some healthcare applications of AI, one or more principles may be especially salient. For example, when considering effects of AI on the patient experience, the principle of Beneficence becomes prominent. Humanistic care emphasizes the need to develop treatment plans that are tailored to the individual needs of patients (56, 103). Future advancements in AI models are needed to enable deeper understanding of contextual nuances of specific patients' situations and to address subtle differences in individual and societal contexts, fostering a more holistic and patient-centered approach to healthcare (55).

This review has several limitations. Firstly, our use of two databases (PubMed and EMBASE) and constrained search terms may have missed relevant papers. Secondly, due to our focus on mapping our results onto an ethical framework and our inclusion of non-empirical studies and the limited empirical literature in this area, it was beyond the scope of this study to evaluate the quality of existing empirical literature on healthcare AI. Finally, AI research is evolving at a rapid pace, necessitating a dynamic dialogue around its advancements and implications. Our review, which by nature is a static summary based on past literature, represents only a snapshot of existing knowledge up to this point, potentially lagging behind the most recent developments in the field, which future studies should continue to address.

In conclusion, risks associated with the application of AI in healthcare may be high stakes (104, 105). Medical decisions directly impact the quality of life, health, and safety of patients, meaning that any technical errors or ethical misconduct can have serious consequences. Given the already extensive adoption and evolving use cases of AI in healthcare—from using AI to generate research questions, to diagnostic assistance and personalized treatment planning, to patient monitoring—ensuring technological reliability and ethical protections has become particularly critical. Our research suggests that the four widely accepted principles of biomedical ethics (Beneficence, Non-Maleficence, Respect for Autonomy, and Justice) provide a relevant foundation for ethical evaluation of AI engagement in healthcare to build upon and can be used to ground interpretation and prioritization in other Responsible AI Frameworks. These four principles can be an organizing framework to address gaps that may exist in current policies and regulations, in addition to bridging, clarifying, prioritizing, while also offering a “safety net” for handling healthcare specific ethical concerns. By grounding emerging regulatory mechanisms in these established ethical guidelines, we can better construct a comprehensive governance system that promotes the responsible application of AI in the healthcare sector.
