# Supplementary material for: Ethics of AI in healthcare: a scoping review demonstrating applicability of a foundational framework
Source: Front Digit Health. 2025 Sep 10;7:1662642. doi: 10.3389/fdgth.2025.1662642 (PMC12459206; doi:10.3389/fdgth.2025.1662642)
Supplement: Supplementary file 1 [file Supplementaryfile1.docx]

Supplementary Material

# Supplementary Tables and Figures

## Supplementary Tables

**Supplementary Table 1.** Preferred Reporting Items for Systematic reviews and Meta-Analyses extension for Scoping Reviews (PRISMA-ScR) Checklist

| **SECTION** | **ITEM** | **PRISMA-ScR CHECKLIST ITEM** | **REPORTED ON PAGE #** |
| --- | --- | --- | --- |
| **TITLE** | | | |
| Title | 1 | Identify the report as a scoping review. | 1 |
| **ABSTRACT** | | | |
| Structured summary | 2 | Provide a structured summary that includes (as applicable): background, objectives, eligibility criteria, sources of evidence, charting methods, results, and conclusions that relate to the review questions and objectives. | 3-8 |
| **INTRODUCTION** | | | |
| Rationale | 3 | Describe the rationale for the review in the context of what is already known. Explain why the review questions/objectives lend themselves to a scoping review approach. | 5-6 |
| Objectives | 4 | Provide an explicit statement of the questions and objectives being addressed with reference to their key elements (e.g., population or participants, concepts, and context) or other relevant key elements used to conceptualize the review questions and/or objectives. | 6-7 |
| **METHODS** | | | |
| Protocol and registration | 5 | Indicate whether a review protocol exists; state if and where it can be accessed (e.g., a Web address); and if available, provide registration information, including the registration number. | N/A |
| Eligibility criteria | 6 | Specify characteristics of the sources of evidence used as eligibility criteria (e.g., years considered, language, and publication status), and provide a rationale. | 6-7 |
| Information sources* | 7 | Describe all information sources in the search (e.g., databases with dates of coverage and contact with authors to identify additional sources), as well as the date the most recent search was executed. | 6 |
| Search | 8 | Present the full electronic search strategy for at least 1 database, including any limits used, such that it could be repeated. | SA2 |
| Selection of sources of evidence† | 9 | State the process for selecting sources of evidence (i.e., screening and eligibility) included in the scoping review. | 6-7 |
| Data charting process‡ | 10 | Describe the methods of charting data from the included sources of evidence (e.g., calibrated forms or forms that have been tested by the team before their use, and whether data charting was done independently or in duplicate) and any processes for obtaining and confirming data from investigators. | 7-8 |
| Data items | 11 | List and define all variables for which data were sought and any assumptions and simplifications made. | 7-8 |
| Critical appraisal of individual sources of evidence§ | 12 | If done, provide a rationale for conducting a critical appraisal of included sources of evidence; describe the methods used and how this information was used in any data synthesis (if appropriate). | N/A |
| Synthesis of results | 13 | Describe the methods of handling and summarizing the data that were charted. | 7-8 |
| **RESULTS** | | | |
| Selection of sources of evidence | 14 | Give numbers of sources of evidence screened, assessed for eligibility, and included in the review, with reasons for exclusions at each stage, ideally using a flow diagram. | 8 |
| Characteristics of sources of evidence | 15 | For each source of evidence, present characteristics for which data were charted and provide the citations. | SA4 |
| Critical appraisal within sources of evidence | 16 | If done, present data on critical appraisal of included sources of evidence (see item 12). | N/A |
| Results of individual sources of evidence | 17 | For each included source of evidence, present the relevant data that were charted that relate to the review questions and objectives. | SA4 |
| Synthesis of results | 18 | Summarize and/or present the charting results as they relate to the review questions and objectives. | 8-14 |
| **DISCUSSION** | | | |
| Summary of evidence | 19 | Summarize the main results (including an overview of concepts, themes, and types of evidence available), link to the review questions and objectives, and consider the relevance to key groups. | 14-16 |
| Limitations | 20 | Discuss the limitations of the scoping review process. | 16 |
| Conclusions | 21 | Provide a general interpretation of the results with respect to the review questions and objectives, as well as potential implications and/or next steps. | 17 |
| **FUNDING** | | | |
| Funding | 22 | Describe sources of funding for the included sources of evidence, as well as sources of funding for the scoping review. Describe the role of the funders of the scoping review. | 17 |

JBI = Joanna Briggs Institute; PRISMA-ScR = Preferred Reporting Items for Systematic reviews and Meta-Analyses extension for Scoping Reviews.

* Where *sources of evidence* (see second footnote) are compiled from, such as bibliographic databases, social media platforms, and Web sites.

† A more inclusive/heterogeneous term used to account for the different types of evidence or data sources (e.g., quantitative and/or qualitative research, expert opinion, and policy documents) that may be eligible in a scoping review as opposed to only studies. This is not to be confused with *information sources* (see first footnote).

‡ The frameworks by Arksey and O’Malley (6) and Levac and colleagues (7) and the JBI guidance (4, 5) refer to the process of data extraction in a scoping review as data charting*.*

§ The process of systematically examining research evidence to assess its validity, results, and relevance before using it to inform a decision. This term is used for items 12 and 19 instead of "risk of bias" (which is more applicable to systematic reviews of interventions) to include and acknowledge the various sources of evidence that may be used in a scoping review (e.g., quantitative and/or qualitative research, expert opinion, and policy document).

*From:* Tricco AC, Lillie E, Zarin W, O'Brien KK, Colquhoun H, Levac D, et al. PRISMA Extension for Scoping Reviews (PRISMAScR): Checklist and Explanation. Ann Intern Med. 2018;169:467–473. [doi: 10.7326/M18-0850](http://annals.org/aim/fullarticle/2700389/prisma-extension-scoping-reviews-prisma-scr-checklist-explanation).

**Supplementary Table 2.** Detailed Search Strategy

| **Preliminary searches (conducted in both PubMed and Embase in January – February 2024)** |
| --- |
| 1. “Artificial intelligence” OR “Generative AI” OR “Generative artificial intelligence” OR “Machine learning models” OR “Natural Language Processing” OR “Neural Networks” OR “Computer-Assisted Instruction” OR “Algorithms” OR “Pattern Recognition” OR “decision making” OR “Automated” OR “Sentiment Analysis” OR “Transfer Learning” OR “Pretrained Models” OR “Supervised learning” OR “Unsupervised learning” OR “machine learning”  - AND - Health* OR healthcare OR "health care" OR clinical OR "mental health" OR psychotherap* OR "health research" OR "digital health" OR "digital healthcare" OR "digital health care") - AND - Ethic* OR “ethical concern*” OR “ethical issue*” OR “ethical consideration*” OR dilemma* OR “ethical dilemma*” OR “ethical implication*” OR beneficence OR nonmaleficence OR empath* OR humanis* OR justice OR equity OR “respect for person*”  1. “Artificial intelligence” OR “Generative AI” OR “Generative artificial intelligence” OR “Machine learning models” OR “Natural Language Processing” OR “Neural Networks” OR “Computer-Assisted Instruction” OR “Algorithms” OR “Pattern Recognition” OR “decision making” OR “Automated” OR “Sentiment Analysis” OR “Transfer Learning” OR “Pretrained Models” OR “Supervised learning” OR “Unsupervised learning” OR “machine learning”  - AND - Health* OR healthcare OR "health care" OR clinical OR "mental health" OR psychotherap* OR "health research" OR "digital health" OR "digital healthcare" OR "digital health care")  1. "Human-AI" OR "human-AI complementarity" OR “human artificial intelligence complementarity” OR "Human-AI collaboration" OR "Human artificial intelligence collaboration" OR "Human-AI partnership*" OR "Human artificial intelligence partnership*" OR "Human-AI teaming" OR "Human artificial intelligence teaming" OR "Human-robot collaboration*" OR "Human-in-the-loop" OR "human artificial intelligence" OR "human machine" OR "human agent" OR "Collaborative intelligence" OR "Human-computer interaction*" OR “agent collaboration*” OR “Artificial agent*” OR “augmented intelligence” OR “human artificial intelligence augmentation”  - AND - Health* OR healthcare OR "health care" OR clinical OR "mental health" OR psychotherap* OR "health research" OR "digital health" OR "digital healthcare" OR "digital health care")  1. "Human-AI" OR "human-AI complementarity" OR “human artificial intelligence complementarity” OR "Human-AI collaboration" OR "Human artificial intelligence collaboration" OR "Human-AI partnership*" OR "Human artificial intelligence partnership*" OR "Human-AI teaming" OR "Human artificial intelligence teaming" OR "Human-robot collaboration*" OR "Human-in-the-loop" OR "human artificial intelligence" OR "human machine" OR "human agent" OR "Collaborative intelligence" OR "Human-computer interaction*" OR “agent collaboration*” OR “Artificial agent*” OR “augmented intelligence” OR “human artificial intelligence augmentation |
| **Final search (conducted in both PubMed and Embase on February 17, 2024)** |
| "Human-AI" OR "human-AI complementarity" OR “human artificial intelligence complementarity” OR "Human-AI collaboration" OR "Human artificial intelligence collaboration" OR "Human-AI partnership*" OR "Human artificial intelligence partnership*" OR "Human-AI teaming" OR "Human artificial intelligence teaming" OR "Human-robot collaboration*" OR "Human-in-the-loop" OR "human artificial intelligence" OR "human machine" OR "human agent" OR "Collaborative intelligence" OR "Human-computer interaction*" OR “agent collaboration*” OR “Artificial agent*” OR “augmented intelligence” OR “human artificial intelligence augmentation”  AND  “Artificial intelligence” OR “Generative AI” OR “Generative artificial intelligence” OR “Machine learning models” OR “Natural Language Processing” OR “Neural Networks” OR “Computer-Assisted Instruction” OR “Algorithms” OR “Pattern Recognition” OR “decision making” OR “Automated” OR “Sentiment Analysis” OR “Transfer Learning” OR “Pretrained Models” OR “Supervised learning” OR “Unsupervised learning” OR “machine learning” |

**Supplementary Table 3:** Population/Concept/Context (PCC) Eligibility Criteria

| **Criterion** |  |
| --- | --- |
| **P (Population)** | **AI (Specifically, Human-AI partnerships / Human-AI teaming)**  *Working definition:*  Human-AI teaming is a process between one or more human(s) and one or more AI system(s) acting as team members with unique and complementary capabilities, who work interdependently toward a common goal. The team members' roles are dynamically adapting throughout the collaboration, requiring coordination and mutual communication to meet each other's and the task's requirements. For this, a common goal, shared situational awareness, and trust within the team are necessary.(1) |
| **C (Concept)** | **Ethics**  Included article abstracts / articles should either:   1. include a broad mention of ethics using words such as “ethics” or “ethical, OR 2. include at least two phrases concerning ethical issues   *Working definition:*  From Cambridge Dictionary: “a system of accepted beliefs that control behavior, especially such a system based on morals.”  If unsure how to determine whether a specific topic is related to ethics, at minimum, include ANY considerations that could fall under at least one of these four principles:  (1) respect for autonomy (a principle requiring respect for the decision-making capacities of autonomous persons)  (2) nonmaleficence (a principle requiring not causing harm to others)  (3) beneficence (a group of principles requiring that we prevent harm, provide benefits, and balance benefits against risks and costs)  (4) justice (a group of principles requiring appropriate distribution of benefits, risks, and costs fairly).  Note: The articles that we will include may be focused on a specific ethical issue pertaining to health-related AI-human partnerships (e.g., equity, empathy, privacy, etc.), or they may be broader papers about health-related AI-human-partnerships that include content about ethical issues.(2,3) |
| **C (Context)** | Healthcare (By definition, healthcare involving AI is considered digital health)  *Working definition:*  “Digital health is the field of knowledge and practice associated with the development and use of digital technologies to improve health.”(4) (e.g., AI-assisted clinical health care, wearable apps, electronic medical records, health research, etc.)  Note: the articles that we will include may be about AI-human partnerships in a specific health field or intervention (e.g., radiologic imaging, psychotherapy, etc.), or they may be broader papers about AI-human-partnerships that include at least some reference to health care, research, or technologies in the abstract. |

**Supplementary Table 4:** Data Extracted From All Articles

| **Authors** | **Year** | **Country** | **Aims/purpose** | **Study population and sample size** | **Methodology   (For intervention studies: type/duration, comparison group, and outcomes)** | **Main findings / conclusions relevant to Healthcare AI** | **Ethical principles relevant to content** | **Specific codes applied** |
| --- | --- | --- | --- | --- | --- | --- | --- | --- |
| Abraham et al.(5) | 2021 | United States | To use a human-centered approach to identify factors affecting sustainability of postoperative handoff standardization and explore potential for AI risk assessment during handoffs. | 5 clinical anesthesiology fellows, 9 ICU registered nurses, and 10 anesthesiology/critical care residents | Mixed qualitative study | Clinicians had divergent views on AI-generated postoperative risks. | 2.Non-maleficence | （2）Risk; error management |
| Abràmoff et al.(6) | 2022 | United States | To initiate dialogue on issues to consider regarding ethics of AI that uses images of eye structures. | N/A | Literature review | AI could improve health care access and patient outcomes. Nevertheless, substantial ethical concerns exist. | 1.Beneficence 2.Non-maleficence 3.Justice 4.Respect for autonomy | （1）Quality and safety of care/Clinical validity of algorithms （2）Misuse/Risk; error management （3）Bias/Clear assumption of responsibility, accountability （4）Transparency or understandability |
| Adams et al.(7) | 2023 | United States | To examine strengths and limitations of integrating AI and automated technologies into pain medicine. | N/A | Literature review | AI introduces potential for pain providers to have higher quality connections with patients and manage complex data. On the other hand, concerns exist around data quality and decision-making. | 1.Beneficence 2.Non-maleficence 3.Justice | （1）Clinical validity of algorithms/Accuracy and efficiency  （2）Privacy and data protection （3）Equity, fairness |
| Adida et al.(8) | 2024 | United States | To provide an overview of current machine learning (ML) modalities, limitations, and future directions in spine surgery. | N/A | Narrative review | Current limitations associated with ML can be overcome using advanced algorithms that account for biases and generalizability and consideration of privacy, legal, and ethical concerns. | 2.Non-maleficence 3.Justice 4.Respect for autonomy | （2） Insufficient data volume, generalizability concerns/Low data quality, accuracy and reliability concerns （3）Bias, discrimination （4）Transparency or understandability |
| Afnan et al.(9) | 2021 | United Kingdom | To provide recommendations on ethical use of AI for IVF embryo selection. | N/A | Editorial | ML models should be interpretable by clinicians and patients and rigorously evaluated using RCTs. | 3.Justice 4.Respect for autonomy | （3）Bias, discrimination/Clear assumption of responsibility, accountability （4）Transparency or understandability |
| Agmon et al.(10) | 2022 | Israel | To analyze gender bias in clinical trials, design an algorithm that mitigates effects of gender bias on NLP systems trained on text drawn from clinical trials, and to evaluate the algorithm's performance. | 16,772 clinical trial abstracts | Quantitative descriptive analysis | Women were significantly underrepresented in clinical trials. The algorithm designed in this study enables gender-sensitive use of publications as training data for NLP word embeddings. | 3.Justice | （3）Bias, discrimination |
| Ahmad et al.(11) | 2019 | United Kingdom | To describe the potential benefits and barriers to using computer-aided detection and diagnosis systems that rely on AI, in colonoscopies. | N/A | Editorial | Clinical trials utilizing AI-powered software during endoscopy for detection and diagnosis are imminent. Regulatory concerns include lack of transparency and complexity involved in DL-based decision-making. | 4.Respect for autonomy | （4）Transparency or understandability |
| Ahmed et al.(12) | 2017 | Italy | To describe cognitive computing in health care and IBM's efforts in this area. | N/A | Editorial | Cognitive computing enables more personalized care to patients with greater diagnostic certainty. | 1.Beneficence | （1）Personalized medicine/Promotion of well-being/Clinician–patient relationship and communication |
| Ahmed et al.(13) | 2020 | United States | To describe literature on AI and ML approaches to health care, precision medicine, and resource management. | N/A | Literature review | AI and ML approaches have potential to enable better health care at lower costs and better work-life balance for clinicians. However, various ethical concerns exist. | 1.Beneficence 2.Non-maleficence 4.Respect for autonomy | （1）Accuracy and efficiency  （2）Privacy and data protection/Low data quality, accuracy and reliability concerns （4）Shared decision-making |
| Alfano et al.(14) | 2024 | Italy | To discuss ethical considerations surrounding use of AI in psychotherapy, particularly the need for responsible implementation, patient privacy, and human-AI interaction. | N/A | Editorial | A proactive approach is needed to address associated ethical considerations of AI in psychotherapy while leveraging potential benefits. | 1.Beneficence 2.Non-maleficence 4.Respect for autonomy | （1）Dignity, empathy, and humanism in healthcare （2）Need for human regulation; oversight/Risk; error management/ （4）Autonomy/informed consent/ |
| Alhasan et al.(15) | 2023 | Saudi Arabia | To highlight benefits that ChatGPT may have for pediatric nephrology. | N/A | Editorial | ChatGPT offers potential to quickly synthesize information, enhance clinical communication, and encourage interdisciplinary collaboration. Ethical concerns include potential for misinformation. | 1.Beneficence 2.Non-maleficence 3.Justice | （1）Clinician–patient relationship and communication/Promotion of well-being/ Accuracy and efficiency  （2）Need for human regulation; oversight/Misinformation/Low data quality, accuracy and reliability concerns （3）Accessibility of services |
| Allam et al.(16) | 2014 | Switzerland | To compare effects of search engines that show websites emphasizing pro- versus anti- vaccination information, compared with normal Google results as a control group. | 39 American marketing students; 197 Amazon Mechanical Turk participants | Randomized controlled trial  (Two online experiments: (1) pre/post design; two manipulated search engines versus normal Google control; (2) post-test design; five manipulated search engines versus normal Google control. Outcomes: knowledge, beliefs, and attitudes about vaccination.) | Pro- and anti- vaccination content on websites had significant effects on knowledge, beliefs, and attitudes. Selection and sorting algorithms of search engines are influential in online health information seeking. | 2.Non-maleficence | （2）Low data quality, accuracy and reliability concerns/Trust in implementing AI for healthcare/Privacy and data protection |
| Allouch et al.(17) | 2021 | Israel | To describe benefits of conversational agents, technologies used to develop them, and associated moral and security issues. | N/A | Literature review | Some applications of conversational agents are positive; others should be monitored or even avoided for moral reasons. | 2.Non-maleficence | （3）Trust in implementing AI for healthcare/Privacy and data protection |
| Almazyad et al.(18) | 2023 | Saudi Arabia | To evaluate ChatGPT-4's ability to summarize key themes from medical conference panel recommendations on pediatric palliative care do-not-resuscitate conflict resolutions. | 4 palliative care experts (panel) and 70 attendees of the first Pan-Arab Pediatric Palliative Critical Care Hybrid Conference. | ChatGPT-4-integrated qualitative thematic analysis | ChatGPT-4 effectively facilitated complex do-not-resuscitate conflict resolution by summarizing key themes from expert panel discussions. | 1.Beneficence 3.Justice 4.Respect for autonomy | （1）Patient-centered care/Accuracy and efficiency  （3）Cultural diversity, sensitivity, and exclusivity/ （4）autonomy |
| Alrassi et al.(19) | 2021 | United States | To emphasize the critical role of human oversight in safe and effective patient care even as medical practice is increasingly guided by AI. | N/A | Editorial | AI has immense value for medicine, but it must complement, not replace, critical human skills that form the foundation of patient care. | 1.Beneficence 2.Non-maleficence 4.Respect for autonomy | （1）Patient-centered care/Clinician–patient relationship and communication （2）Risk; error management/Low data quality, accuracy and reliability concerns （4）Shared decision-making/Autonomy/ |
| Altamimi et al.(20) | 2023 | Saudi Arabia | To discuss AI chatbots as potential supplements rather than substitutes for medical professionals. | N/A | Editorial | AI chatbots are a powerful supplement to healthcare but should not be seen as a complete substitute for medical professionals. | 2.Non-maleficence 4.Respect for autonomy | （2）Need for human regulation; oversight/Privacy and data protection/misuse （4）Transparency or understandability/Informed consent |
| AlZaabi et al.(21) | 2023 | Sultanate of Oman | To evaluate physicians and medical students' attitudes and perceptions of AI applications in healthcare. | 82 physicians and 211 medical students | Cross-sectional quantitative study | Most of the medical employees and students were aware of the Use of AI and showed willingness to incorporate AI if there is a adequate training and protocol. However, there are some concerns about AI's inability to respond in unexpected scenarios, which can lead to medical errors. | 1.Beneficence 2.Non-maleficence 3.Respect for autonomy | （1）Accuracy and efficiency /Dignity, empathy, and humanism in healthcare/Clinical validity of algorithms/Promotion of well-being （2）Trust in implementing AI for healthcare/Need for human regulation; oversight （4）autonomy/ |
| Anderson et al.(22) | 2019 | United States | To examine how effectively an AI can replace human pathologists in detecting cancer cells. | N/A | Case Study | In order to use AI approach in medical field, the decisions regarding incorporating AI technologies should be made by review board, not only by physicians. | 2.Non-maleficence 3.Justice 4.Respect for autonomy | （1）Risk; error management （2）Bias, discrimination/ （3）Transparency or understandability/ |
| Angelo-poulou et al.(23) | 2022 | United Kingdom | To evaluate the quality of AI explanation in a scientific context. | N/A | Editorial | The quality of AI explanation can be evaluated by four different aspects: (a) Philosophical foundations of what an explanation is; (b) social attribution: how people explain behavior; (c) Cognitive processes underpinning how people explain and evaluate explanations; (d) Social explanation, or how people communicate explanations. | 2.Non-maleficence | （2）Privacy and data protection |
| Arnold(24) | 2021 | Australia | To examine unidentified epistemic, ontologic, ethical, legal, and sociopolitical challenges that AI in Medicine poses. | N/A | Editorial | Physicians should neither uncritically accept nor unreasonably resist developments in AI but must actively engage and contribute to the discourse since AI will affect their roles and the nature of their work. | 1.Beneficence 2.Non-maleficence 3.Justice 4.Respect for autonomy | （1）Clinician–patient relationship and communication/Patient-centered care （2）Low data quality, accuracy and reliability concerns/Risk; error management/Need for human regulation; oversight/Privacy and data protection/Misinformation （3）Equity, fairness/Bias, discrimination/Accessibility of services/Clear assumption of responsibility, accountability （4）Autonomy/Transparency or understandability/Shared decision-making |
| Arshad et al.(25) | 2023 | United States | To examine ChatGPT's capabilities and risks in conducting medical hospital level research. | N/A | Literature Review | Introduction of AI and chatbots in medical research is useful for academic writing, data analysis, statistics handling, and code generation, but there are risks of data security, bias, or error in output. Thus, using AI needs more considerations to ensure ethical standards. | 1.Beneficence 2.Non-maleficence 3.Justice 4.Respect for autonomy | （1）Accuracy and efficiency/ （2）Privacy and data protection/Misinformation/Low data quality, accuracy and reliability concerns/Need for human regulation/ （3）Bias, discrimination/ （4）Transparency or understandability/ |
| Athreya et al.(26) | 2019 | United States | To describe the feasibility of integrating machine learning with functionally validated pharmacogenomic SNP biomarkers to achieve accurate prediction of a treatment response. | N/A | Literature Review | Combination of pharmacogenomics and machine learning is a promising way to achieve the prediction of therapeutic outcomes of antidepressant treatment in patients with MDD with sufficient accuracy for use in the clinic. However, we should be wary of potential methodological pitfalls associated with ML and AI. | 1.Beneficence 3.Justice 4.Respect for autonomy | （1）Accuracy and efficiency/Clinical validity of algorithms （3）Bias, discrimination （4）Transparency or understandability/ |
| Aung et al.(27) | 2021 | United Kingdom | To evaluate AI's present applications in healthcare, including its benefits, limitations and future scope. | N/A | Literature Review | There are both areas of agreements and controversies regarding use of AI in patient care. AI could transform physician workflow, from assisting physicians and replacing administrative tasks. However, since there are challenges training ML systems and unclear accountability, AI's implementation is difficult, and physicians lack understanding of what AI implementation could represent. | 1.Beneficence 2.Non-maleficence 3.Justice 4.Respect for autonomy | （1）Accuracy and efficiency/Personalized medicine/Clinical validity of algorithms/ （2）Insufficient data volume, generalizability concerns/Privacy and data protection/Risk; error management/Trust in implementing AI for healthcare/ （3）Affordability and availability/Bias, discrimination/Clear assumption of responsibility, accountability/ （4）Transparency or understandability/ |
| Ayorinde et al.(28) | 2022 | United Kingdom | To examine challenges that arise in application of AI to renal histopathology, considering areas where choices around model architecture, training strategy, and workflow design may be influenced by factors beyond final performance metrics. | N/A | Literature Review | AI assessment tools are anticipated to provide substantial benefits to renal histopathologic expertise and renal digital slide assessments. However, since there are some areas where AI assessment tools consider tasks in fundamentally different ways to the human assessor, the best way to use AI automation would be to blend it with human supervision. | 2.Non-maleficence 3.Justice 4.Respect for autonomy | （1）Trust in implementing AI for healthcare/Risk; error management （3）Bias, discrimination （4）Transparency or understandability/ |
| Badri et al.(29) | 2018 | Canada | To examine changes and consequences of Occupational Health and Safety (OHS) in Industry 4.0 in order to raise consciousness with regard to integration of OHS into Industry 4.0 | N/A | Literature Review | If the technologies driving Industry 4.0 develop in silos and manufacturers’ initiatives are isolated and fragmented, net impact on OHS will be negative. As major changes are implemented, previous gains in preventive management of workplace health and safety will be at risk. Researchers, field experts and industrialists will have to collaborate on a smooth transition. | 1.Beneficence 2.Non-maleficence 3.Justice | （1）Quality and safety of care/Accuracy and efficiency （2）Need for human regulation; oversight/Risk; error management （3）Affordability and availability |
| Bakken(30) | 2023 | United States | To review papers focused on AI that provide key lessons about the importance of keeping the human in the loop. | N/A | Editorial | To advance application of AI in health that results in safe, high-quality, and equitable care and improved quality of life, it is essential to focus not only on the technical quality of the AI but also on the critical factors related to implementation and use. | 2.Non-maleficence 4.Respect for autonomy | （2）Low data quality, accuracy and reliability concerns （4）Transparency or understandability |
| Balcombe and De Leo(31) | 2021 | Australia | To effectively integrate digital mental health solutions into mental health care service systems, and to apply these technologies among various populations. | N/A | Literature Review | Digitalization and data science should prioritize quality, security, adaptability, technical excellence, positive empowerment, and motivation. End-users should use innovative research methods to overcome difficulties of evaluation through traditional research methods. | 1.Beneficence 2.Non-maleficence 3.Justice 4.Respect for autonomy | （1）Promotion of well-being （2）Low data quality, accuracy and reliability concerns/Privacy and data protection （3）Cultural diversity, sensitivity, and exclusivity/Equity, fairness/Bias, discrimination （4）Transparency or understandability |
| Bandyopadhyay and Goldstein(32) | 2023 | United States | To provide a concise overview of relevant terminology, definitions, and use cases of AI in sleep medicine through a thorough review of relevant published literature | N/A | Literature Review | AI may improve patient care, enhance diagnostic abilities, and augment management of sleep disorders. However, there is a need to regulate and standardize existing ML algorithms prior to its inclusion in sleep clinics. | 1.Beneficence 2.Non-maleficence 3.Justice | （1）Accuracy and efficiency /Clinical validity of algorithms/Promotion of well-being/Clinician–patient relationship and communication （2）Insufficient data volume, generalizability concerns/Low data quality, accuracy and reliability concerns/Privacy and data protection （3）Equity, fairness |
| Banerjee et al.(33) | 2023 | United States | To examine various types of bias from shortcut learning that may occur at different phases of AI model development and provide solutions that can be used to mitigate bias to be applied in medical AI. | N/A | Literature Review | Various tool kits and techniques can be used to evaluate and mitigate bias in medical AI. Ongoing legal changes where use of a biased model will be penalized highlight the necessity of understanding, detecting, and mitigating biases from shortcut learning and will require research examining the whole AI pipeline. | 1.Beneficence 2.Non-maleficence 3.Justice | （1）Accuracy and efficiency （2)Insufficient data volume, generalizability concerns （3）Bias, discrimination/Equity, fairness |
| Bartenschlager et al.(34) | 2023 | Germany | To evaluate the performance of algorithms for triaging COVID-19 patients, through an ethical perspective. | Multicenter dataset with more than 4,000 Covid-19 patients from the Lean European Open Survey on COVID-19 Patients registry. | Quantitative descriptive analysis | Data-driven manipulation of the existing human-made base triage algorithm can improve classification, but AI adaptations promise superior performance. Use of an integrated human-AI algorithm is recommended. | 1.Beneficence 4.Respect for autonomy | （1）Accuracy and efficiency （4）Transparency or understandability |
|  |  |  |  |  |  |  |  |  |
| Batlle et al.(35) | 2021 | United States | To report results from an American College of Radiology data sharing workshop aiming to develop philosophies around best practices in sharing of health information. | N/A | Workshop/conference report | Data sharing involves ethical, practical, technical, legal, and commercial challenges that require a thoughtful, considered approach. | 1.Beneficence 2.Non-maleficence 4.Respect for autonomy | （1）Dignity, empathy, and humanism in healthcare （2）Privacy and data protection （4）Informed consent/Transparency or understandability |
| Baumgartner(36) | 2023 | Australia | To assess the potential impact of ChatGPT on clinical and translational medicine. | N/A | Literature review | ChatGPT has the potential to revolutionize the way information is disseminated and processed in clinical and translational medicine. | 1.Beneficence 2.Non-maleficence 3.Justice | （1）Accuracy and efficiency/Patient-centered care （2）Privacy and data protection/Risk; error management/Low data quality, accuracy and reliability concerns/Misinformation （3）Bias, discrimination |
| Baumgartner et al.(37) | 2023 | Netherlands | To report results from a discussion held during the international conference "Fair Medicine and AI" about ethical and legal opportunities and challenges of AI in healthcare. | N/A | Workshop/conference report | There is a dearth of studies on how human-AI interaction may best be configured to deliver safe, effective, and equitable healthcare. It is important to include social science in the development of intersectionally beneficent and equitable AI for biomedical research and healthcare. | 1.Beneficence 2.Non-maleficence 3.Justice 4.Respect for autonomy | （1）Accuracy and efficiency/Personalized medicine/Patient-centered care/Clinician–patient relationship and communication （2）Insufficient data volume, generalizability concerns/Privacy and data protection （3）Bias, discrimination/Equity, fairness （4）Transparency or understandability |
| Bays et al.(38) | 2023 | United States | To provide an overview of Artificial Intelligence in the management of patients with obesity, from the Obesity Medicine Association. | N/A | Position statement | Chatbots may be a helpful source of information for clinicians and may be helpful in drafting administrative writing. Challenges of Artificial Intelligence include privacy and security, accuracy and reliability, and potential perpetuation of systemic biases. | 1.Beneficence 2.Non-maleficence 4.Respect for autonomy | （1）Emotional support/Personalized medicine （2）Low data quality, accuracy and reliability concerns;Misinformation （4）Informed consent |
| Bazoukis et al.(39) | 2022 | Cyprus | To describe skepticism and concern around AI algorithms and offer an integrated regulatory framework to AI developers, clinicians, and researchers. | N/A | Literature review | Growth of healthcare AI is expected to involve many stakeholders and should be guided by a stepwise approach as well as the necessity to serve every patient. | 2.Non-maleficence 3.Justice | （2）Privacy and data protection/Low data quality, accuracy and reliability concerns/Need for human regulation; oversight/Risk; error management （3）Clear assumption of responsibility, accountability/Equity, fairness/Bias, discrimination/Insufficient data volume, generalizability concerns |
| Bekbolatova et al.(40) | 2024 | United States | To examine the current use and risks of AI in medical field and consider future application of AI in medicine. | N/A | Literature review | AI should not be viewed as a replacement for clinicians but as an adjunct tool. Rather than eliminating jobs, AI is likely to transform jobs, shifting clinicians’ emphasis to duties that complement AI. | 1.Beneficence 2.Non-maleficence 3.Justice 4.Respect for autonomy | （1）Accuracy and efficiency /Promotion of well-being/Personalized medicine/Patient-centered care/Promotion of well-being （2）Privacy and data protection/Need for human regulation; oversight/Trust in implementing AI for healthcare （3）Accessibility of services/Bias, discrimination （4）Informed consent/Shared decision-making |
| Beltrami et al.(41) | 2022 | United States | To describe use of AI in skin cancer detection. | N/A | Literature review | AI-augmented detection of skin cancer has potential to improve quality of life, reduce costs, and promote access to skin assessment. Dermatologists play a critical role in responsible development and deployment of AI for skin cancer. | 1.Beneficence 2.Non-maleficence | （1）Promotion of well-being （2）Risk; error management/Misuse |
| Benzinger et al.(42) | 2023 | Germany | To provide a comprehensive overview of pros and cons of the use of AI in clinical practice. | 44 papers | Qualitative text analysis | Artificial intelligence may increase patient autonomy and beneficence through accurate and reliable statistical predictions. However, there are also concerns that reducing ethical decision making to statistical correlations may limit patient autonomy. | 1.Beneficence 2.Non-maleficence 4.Respect for autonomy | （1）Accuracy and efficiency /Personalized medicine/Dignity, empathy, and humanism in healthcare/Promotion of well-being （2）Misinformation （4）Autonomy/Shared decision-making |
| Bergquist et al.(43) | 2023 | United States | To evaluate applications of ML in healthcare predictions. | 345 registered participants, comprising 25 independent teams | Quasi-experimental study   (Participating teams generated 25 accurate models each trained on a dataset of over 1.1 million patients and evaluated on patients who were prospectively collected over a 1-year observation within a large health system. Results were compared across models.) | ML methods may be able to address some causes of treatment disparities but may cause others disparities if trained without rich longitudinal data. Multisite standardized architecture and independent oversight is needed to assess new methods. | 1.Beneficence 2.Non-maleficence | （1）Promotion of well-being （2）Privacy and data protection/Misinformation |
| Bernstein et al.(44) | 2023 | United States | To evaluate quality of ophthalmology advice generated by a large language model (LLM) chatbot in comparison with ophthalmologist-written advice. | LLM Chatbot; 8 board-certified ophthalmologists (evaluators) | Cross-sectional quantitative study | The LLM chatbot in this study was capable of responding to long user-written posts about questions concerning eye health. It largely generated appropriate responses that did not differ significantly from ophthalmologist-written responses in terms of inaccuracy, likelihood of harm, extent of harm, or deviation from professional standards. | 1.Beneficence 2.Non-maleficence 3.Justice 4.Respect for autonomy | （1）Accuracy and efficiency/Promotion of well-being/Personalized medicine/Dignity, empathy, and humanism in healthcare （2）Risk; error management/Privacy and data protection/Misinformation （3）Accessibility of services （4）Informed consent |
| Berridge et al.(45) | 2022 | United States | To describe the development and feasibility of a self-administered web-based intervention for people living with mild Alzheimer Disease (AD) and their family care partners. | 29 mild AD dementia care dyads | Mixed methods descriptive study | Participants viewed the web-based intervention as easy to use and helpful and reported that they would likely recommend it to others. | 2.Non-maleficence 3.Justice 4.Respect for autonomy | （1）Risk; error management （3）Algorithms designed in light of the multidimensionality of health （2）Transparency or understandability |
| Beyeler and Sanchez-Garcia(46) | 2022 | United States | To propose a patient-centered approach to incorporating deep-learning-based visual augmentations into neuroprosthetic devices. | N/A | Editorial | Existing bionic visual devices can be complemented by deep-learning-based computer vision and AI to provide useful visual augmentations for everyday tasks. | 1.Beneficence | （1）Personalized medicine |
| Bhardwaj et al.(47) | 2022 | United States | To discuss opportunities and challenges associated with the expected influx of AI and ML across domains of medicine and healthcare in the near future. | N/A | Literature review | Despite theoretical advantages of artificial intelligence and ML, many practical issues remain to be addressed. | 1.Beneficence 2.Non-maleficence 3.Justice | （1）Promotion of well-being/Clinician–patient relationship and communication （2）Privacy and data protection （3）Accessibility of services |
| Bhattacharyya et al.(48) | 2021 | India | To examine the possibility of using AI in solving financial toxicity problems in cancer care. | N/A | Editorial | Integrating AI can be a way to mitigate financial toxicity at diagnosis. However, difficulties of implementation in low- and middle-income countries need to be considered. Stakeholders in cancer care should come together to educate the public and address expectations and understanding of financial toxicity and AI-based approaches to its mitigation. | 1.Beneficence | （1）Promotion of well-being/Personalized medicine |
| Bhowmik et al.(49) | 2022 | India | To design and evaluate performance of a portable noninvasive blood perfusion imager augmented with machine-learning-based quantitative analytics for screening precancerous and cancerous traits in oral lesions. | 36 healthy volunteers, 14 individuals with oral submucous fibrosis, and 11 individuals with oral squamous cell cancer, ages ranging from 20 to 85 | Cross-sectional quantitative study | The imager performed strongly, with an overall sensitivity >96.66% and specificity of 100% as compared to gold-standard biopsy-based tests. | 4.Respect for autonomy | （4）Informed consent |
| Bickmore and Gruber(50) | 2010 | United States | To describe the potential for using relational artificial agents as adjuncts to clinical psychiatric care, a range of possible applications, and ethical issues in developing and fielding agents in psychiatric interventions. | N/A | Literature review | Relational agents will not be able to replace highly trained experts in the near future. However, they may become helpful allies in the treatment of mental illness, as adjuncts to psychiatric clinicians. | 1.Beneficence 2.Non-maleficence 3.Justice 4.Respect for autonomy | （1）Promotion of well-being/Emotional support/Clinician–patient relationship and communication/Accuracy and efficiency  （2）Promoting social isolation （3）Accessibility of services （4）Autonomy |
| Bishara et al.(51) | 2022 | United States | To describe how AI can be implemented into decisions regarding acute care to reduce medical error and improve outcomes. | N/A | Literature review | ML-based medical decision support systems have significant potential to enhance clinical care across various stages in acute care settings. Fostering collaboration among clinicians, bioethicists, data scientists, and lawyers and addressing potential obstacles, these technological advances can improve patient safety and outcomes while mitigating existing and potential inequities. | 1.Beneficence 2.Non-maleficence 3.Justice 4.Respect for autonomy | (1)Accuracy and efficiency /Personalized medicine (2)Misinformation/Privacy and data protection (3)Clear assumption of responsibility, accountability/Equity, fairness/Bias, discrimination (4)Transparency or understandability/Informed consent |
| Blanco- González et al.(52) | 2023 | Spain | To examine AI's benefits and challenges in pharmaceutical research, and to test the ability of ChatGPT-3.5 to assist with generating an article about this topic. | N/A | ChatGPT-3.5-integrated qualitative thematic analysis | AI has the potential to revolutionize drug discovery by improving the efficiency, accuracy, and personalization of treatments while offering strategies like data augmentation and explainable AI. However, successful implementation depends on high-quality data, addressing ethical concerns, and recognizing AI's limitations. | 1.Beneficence 2.Non-maleficence 3.Justice 4.Respect for autonomy | (1)Personalized medicine (2)Low data quality, accuracy and reliability concerns/Privacy and data protection/ (3)Equity, fairness/Bias, discrimination (4)Shared decision-making |
| Bleher and Braun(53) | 2022 | Germany | To illustrate how clinical decision-making is changed and diffusions of responsibility take place in the application of AI-driven clinical decision support systems. | N/A | Editorial | AI-based clinical decision support systems create shifts in responsibility across causal, moral, and legal levels. A dynamic approach to responsibility that emphasizes control, a participatory approach, and reliable fault management should be adopted. | 1.Beneficence 2.Non-maleficence 3.Justice 4.Respect for autonomy | （1）Clinician–patient relationship and communication （2）Trust in implementing AI for healthcare （3）Bias, discrimination/Clear assumption of responsibility, accountability （4）Transparency or understandability |
| Borgstadt et al.(54) | 2022 | United States | To discuss issues in medicine and the tools available through AI and other tech to address these issues. | 5 panel experts (1 data scientist, 1 program engineer, 1 AI healthcare platform executive, 1 chief medical information officer, 1 medical informatics program fellow) | Qualitative thematic analysis | There is a need for continued research and careful implementation of AI in healthcare. Developing standards, analyzing algorithm effectiveness, and training healthcare professionals will be crucial for safely integrating AI into mainstream medicine. | 1.Beneficence 2.Non-maleficence 4.Respect for autonomy | （1）Quality and safety of care （2）Privacy and data protection （4）Transparency or understandability/Informed consent |
| Boulos et al.(55) | 2018 | England | To explore the potential benefits and challenges of blockchain in health and healthcare. | 40 articles | Literature review | Blockchain technologies face challenges such as interoperability, security, privacy, suitability, and sustainability. These technologies are expected to become increasingly prevalent, powerful, and robust as they become coupled with AI in healthcare. | 2.Non-maleficence | （2）Privacy and data protection |
| Briggs et al.(56) | 2022 | United States | To outline how physicians may utilize digital health to improve their cancer care delivery. | N/A | Narrative review | Current limitations associated with digital health technologies (DHT) can be overcome by developing solutions to issues surrounding the regulation, liability, quality, security, equity, and disbursement of DHT. | 1.Beneficence 2.Non-maleficence 3.Justice 4.Respect for autonomy | （1）Accuracy and efficiency/Clinician–patient relationship and communication/Promotion of well-being/Personalized medicine/Quality and safety of care/Emotional support （2）Low data quality, accuracy and reliability concerns/Need for human regulation; oversight （3）Cultural diversity, sensitivity, and exclusivity/Equity, fairness （4）Shared decision-making/Informed consent/Patients' health-related knowledge |
| Brown et al.(57) | 2023 | England | To suggest how AI could augment clinician care strategies and avoid delayed diagnoses. | 27-year old woman with chest pain and shortness of breath | Case study | AI should be viewed as a supportive tool that augments human expertise. AI can revolutionize healthcare but requires further evaluation and careful integration to ensure optimal patient outcomes. | 1.Beneficence 3.Justice 4.Respect for autonomy | （1）Personalized medicine （3）Bias, discrimination/Clear assumption of responsibility, accountability （4）Transparency or understandability/Shared decision-making/Informed consent |
| Brown et al.(58) | 2023 | United States | To determine whether integrating AI-driven insights from clinical and social determinants of health data can reduce rehospitalization rates among older adults. | 6,371 adult patients across 12 hospitals | Case-control study | AI-enhanced transitional care models have the potential to reduce 30-day rehospitalizations. | 1.Beneficence | （1）Accuracy and efficiency |
| Bunning et al.(59) | 2023 | United States | To expand on existing literature on data and safety monitoring for clinical trials with an emphasis on Data and Safety Monitoring Boards (DSMBs) charged with monitoring real-world trials. | 3 real-world clinical trials | Literature review | Clinical trials can benefit from incorporating real-world data sources to increase generalizability and efficiency; however, this requires robust data science infrastructure and evolving safety monitoring approaches to maintain trial rigor. | 2.Non-maleficence | （2）Privacy and data protection |
| Burlina et al.(60) | 2021 | United States | To evaluate generative methods to mitigate AI bias when diagnosing diabetic retinopathy. | 44,346 participants (88,692 public domain fundi) | Quasi-experimental study (evaluation)  Added clinician-annotated labels to a public domain dataset and created a data imbalance scenario that prevented the training of diagnostic models from using retinal images from individuals with darker skin tones. A baseline deep learning system (DLS) was compared to DLSs that used training data augmented by generative models for de-biasing. | AI diagnostic algorithms can show bias in accuracy across skin tones due to data imbalance and domain generalization. Synthetic image generation techniques could help reduce the bias in retinol diagnostics. | 3.Justice | （3）Bias, discrimination |
| Burr et al.(61) | 2020 | England | To explore literature on the ethics of digital well-being and examine how digital technologies impact human quality of life. | N/A | Literature review | Positive computing, personalized human-computer interaction, autonomy, and self-determination will be central to identifying questions related to the ethics of digital well-being. | 1.Beneficence 2.Non-maleficence 3.Justice 4.Respect for autonomy | （1）Promotion of well-being （2）Privacy and data protection/Technology addiction （3）Clear assumption of responsibility, accountability （4）Autonomy/Transparency or understandability |
| Capelleras et al.(62) | 2024 | Turkey | To explore the integration of ChatGPT in patient care as a tool for postoperative guidance. | N/A | Qualitative descriptive analysis | ChatGPT has the potential to improve patient outcomes. However, AI should not replace personalized advice from health care professionals. | 2.Non-maleficence | （2）Privacy and data protection |
| Chambers and Beaney(63) | 2020 | United Kingdom | Examine how Amazon's Alexa's existing functions could practically support people with chronic health problems by relaying findings of a pilot project conducted on behalf of the Sustainability and Transformation Partnership's (STP's) digital workstream. | N/A | Editorial | Voice-assisted technologies such as Alexa have strong potential for supporting patients in managing chronic health conditions and engaging in primary healthcare. | 1.Beneficence 2.Non-maleficence 3.Justice | （1）Promotion of well-being /Emotional support/Patient-centered care （2）Privacy and data protection （3）Accessibility of services/Equity, fairness |
| Chandler et al.(64) | 2022 | United States | To promote collaborative ML approaches in psychiatry involving expert clinicians throughout the entire development and implementation process. | 79 health participants, 23 participants with affective disorders (846 responses); 120 healthy participants, 105 participants with serious mental illness (1177 responses) | Quasi-experimental study  Created 2 subsets of randomly sampled responses (100 training set, 100 validation set, 100 evaluation set) | Human-in-the-loop ML using active learning can improve model accuracy moreso than classic random sampling. | 3.Justice | （3）Bias, discrimination |
| Chen(65) | 2023 | China | To review the use of AI and augmented reality (AR) technologies in orthopedic arthroscopy surgery, examining their current applications, system compositions, and potential benefits. | N/A | Literature review | AI and AR technology offer significant potential to address precision and personalization limitations in traditional arthroscopic surgery by enabling advanced positioning, navigation, and patient-specific surgical approaches. | 2.Non-maleficence | （2）Privacy and data protection |
| Chen et al.(66) | 2024 | Canada | To explore the effectiveness of ML classifiers using contextual text representation for automatically classifying public service experience (PSE) reports. | 861 PSE Reports | Qualitative descriptive analysis | ML classifiers trained with contextual text representations can significantly improve the accuracy of public service experience (PSE) report classification. | 4.Respect for autonomy | （4）Transparency or understandability |
| Chen et al.(67) | 2021 | United States | To identify risk factors associated with delayed linkage to care for HIV patients with novel ML models and identify high-risk regions. | 1070 individual PLHIV patient-level data | Quantitative descriptive analysis | CD4+ cell count, diagnostic facility type, and zip codes are strong predictors for delayed linkage to care which can be predicted by ML models with and without the CD4+ cell count. | 4.Respect for autonomy | （4）Transparency or understandability |
| Chew(68) | 2022 | Singapore | To examine AI chatbot use for weight loss and identify components for prolonging user engagement. | 2231 participants (23 studies) | Literature review | AI chatbots for weight loss should be designed to be human-like, personalized, and immersive by integrating various user data points including health metrics, personality, behaviors, and emotional states. | 2.Non-maleficence 3.Justice | （2）Privacy and data protection （3）Clear assumption of responsibility, accountability |
| Christodoulou and Tsoucalas(69) | 2023 | The Hellenic Republic | To explore the potential of artificial intelligence (AI) in cardiac surgery and inquire whether robots could replace cardiac surgeons. | N/A | Editorial | While AI has made substantial progress in surgical applications, a fully autonomous robotic surgeon remains beyond current capabilities due to the following: technological complexity, patient safety, and ethical considerations. | 1.Beneficence 2.Non-maleficence 4.Respect for autonomy | （1）Accuracy and efficiency （2）Need for human regulation; oversight/Risk; error management （4）Transparency or understandability |
| Clement and Maldonado(70) | 2021 | United States | To survey methods research on transplant-related AI applications and identify concerns in implementing these tools. | N/A | Literature review | Key challenges of AI in transplant include bias/accuracy, clinical decision process/AI explainability, and AI acceptability criteria. Steps that can be taken to help advance the use of AI in transplant include forming an AI Team at each transplant center, establishing clinical and ethical acceptability criteria, and incorporating AI into the Shared Decision Making Model. | 1.Non-maleficence 2.Beneficence 3.Justice 4.Respect for autonomy | （1）Low data quality, accuracy and reliability concerns/Risk; error management/Need for human regulation; oversight  （2）Promotion of well-being/Clinician–patient relationship and communication/Personalized medicine  （3）Bias, discrimination （4）Transparency or understandability |
| Condado et al.(71) | 2022 | Portugal | To explore ongoing research that considers how assisted living environments can be improved with the design and use of inexpensive robotics, AI techniques, and human-computer interaction methods. | N/A | Editorial | Inexpensive human-computer systems can be useful improving the quality of life for those who have disabilities and older adults. | 2.Non-maleficence | （2）Privacy and data protection |
| Creed et al.(72) | 2022 | United States | To explore the possibility of implementing an automated fidelity-scoring super vision tool in community mental health settings. | 18 community mental health therapists, 8 clinical supervisors, and 4 other clinical leaders | Mixed qualitative study | Mental health professionals initially perceived automated fidelity tools positively, with potential benefits for supervision, training, and professional growth, despite concerns about privacy and tools' limitations in assessing nuanced therapeutic elements. | 1.Beneficence 2.Non-maleficence 3.Justice | （1）Accuracy and efficiency/Clinician–patient relationship and communication （2）Privacy and data protection （3）Cultural diversity, sensitivity, and exclusivity |
| Crigger and Khoury(73) | 2019 | United States | To promote the potential benefits of AI for patients, physicians, and other community members in health care. | N/A | Editorial | Members in the health care community must work together to ensure AI is deployed in ways that promote the quality of care and minimizes potentially disruptive effects. | 1.Beneficence 2.Non-maleficence 3.Justice | （1）Promotion of well-being （2）Privacy and data protection （3）Bias, discrimination/Clear assumption of responsibility, accountability |
| Crigger et al.(74) | 2022 | United States | To provide guidance for utilizing trustworthy AI in health care. | N/A | Literature review | There is no consensus on guiding principles for development and deployment of AI in health care; however, the benefits can be harnessed through focusing on building the necessary evidence, oversight, and infrastructure. | 1.Beneficence 2.Non-maleficence 3.Justice | （1）Promotion of well-being/Patient-centered care （2）Risk; error management/Equity, fairness/Privacy and data protection （3）Bias, discrimination/Affordability and availability |
| Cuocolo and Imbriaco(75) | 2021 | Italy | To discuss current ethical and regulatory issues in radiomics and ML solutions. | N/A | Editorial | Radiomics and ML are still new in healthcare and are not yet ready for use in daily clinical practice, and buyers should understand their standing as beta testers. | 2.Non-maleficence 4.Respect for autonomy | （2）Privacy and data protection （4）Informed consent |
| Currie and Rohren(76) | 2022 | Australia | To highlight challenges across the AI pipeline that require attention to ensure beneficence over maleficence. | N/A | Editorial | AI-augmented health care could be an integral part of the broader strategy convergence on local, national, and global health equities. | 3.Justice | （3）Bias, discrimination/Equity, fairness |
| Dankwa-Mullan and Weeraratne(77) | 2022 | United States | To discuss applications of AI/ML tools in cancer and recommend methods for addressing and mitigating potential bias. | N/A | Editorial | AI and ML technologies can improve cancer care, but addressing embedded biases requires a systemic, collaborative approach that prioritizes health equity, empathy, and transparency across multiple stakeholders. | 1.Beneficence 3.Justice | （1）Personalized medicine/Patient-centered care/Dignity, empathy, and humanism in healthcare （3）Bias, discrimination/Equity, fairness |
| De Batlle et al.(78) | 2023 | Spain | To describe the rationale for the selection of the group of settings used to test the multinational large-scale piloting of the GATEKEEPER platform. | 7 European countries (Cyprus, Germany, Greece, Italy, Poland, Spain, and the United Kingdom); 3 Asian pilots (Hong Kong, Singapore, and Taiwan) | Editorial | Large-scale pilots, each with its specific features, will eventually sustain the creation and development of the European Data Space. | 1.Beneficence | （1）Promotion of well-being /Quality and safety of care |
| Deferio et al.(79) | 2019 | United States | To highlight current challenges for collecting social determinants of health data and provide an overview of possible paths forward that could aid in reducing mental health disparities. | N/A | Editorial | Better data collection and analysis may allow for the development of strategies to aid individual patients in receiving improved care and reducing barriers to mental health care. | 2.Non-maleficence 3.Justice | （2）Privacy and data protection （3）Bias, discrimination |
| Di Nuovo(80) | 2023 | United Kingdom | To encourage biomedical engineers to lead multidisciplinary teams in developing human-centered AI solutions. | N/A | Editorial | Biomedical Engineers have a responsibility to ensure that the benefits of technology has a reach regardless of cognitive ability. | 1.Beneficence 2.Non-maleficence 3.Justice 4.Respect for autonomy | （1）Promotion of well-being （2）Privacy and data protection （3）Bias, discrimination/Equity, fairness （4）Transparency or understandability |
| Diaz-Asper et al.(81) | 2024 | Norway | To encourage psychologists to assess the trustworthiness of NLP-based psychological tools, particularly when non-psychologists develop these tools. | N/A | Editorial | The American Psychological Association Ethics Code could be improved to reflect generally better-accepted principles of AI ethics. | 2.Non-maleficence 3.Justice 4.Respect for autonomy | （2）Privacy and data protection （3）Equity, fairness （4）Transparency or understandability/Informed consent |
| Dixon and Holmes(82) | 2022 | United States | To summarize research addressing bias, equity, and literacy in health delivery systems published in 2021. | N/A | Literature review | There are many strong articles relevant to working to make health delivery systems inclusive. More evidence and leadership are necessary to develop and implement best practices. | 3.Justice | （3）Equity, fairness/Bias, discrimination |
| Doyen and Dadario(83) | 2022 | Australia | To address common pitfalls and challenges in applying ML to healthcare and provide practical guidance for overcoming these issues to enhance clinical applicability and trust in medical AI systems. | N/A | Editorial | AI in healthcare faces challenges such as biases, lack of generalizability, explainability issues, and operational impracticalities. Collaboration between medical professionals and data scientists, standardized frameworks, and robust validation/testing are crucial. Addressing these concerns can optimize AI adoption and improve patient safety, trust, and outcomes in healthcare applications. | 1.Beneficence 2.Non-maleficence 3.Justice 4.Respect for autonomy | （1）Accuracy and efficiency/Promotion of well-being/Quality and safety of care （2）Insufficient data volume, generalizability concerns/Privacy and data protection （3）Bias, discrimination （4）Transparency or understandability |
| Dzobo et al.(84) | 2020 | South Africa | To explore the synergistic potential of combining AI and human intelligence (HI) in biomedical engineering and medicine for responsible innovation and enhanced patient care. | N/A | Literature review | AI enhances diagnostics, drug discovery, and personalized medicine, but cannot replace HI due to the need for empathy and nuanced decision-making. Ethical considerations, governance frameworks, and HI involvement are crucial to mitigate biases, ensure equitable AI use, and build trust. The combination of AI and HI can improve outcomes from prevention to treatment. | 3.Justice 4.Respect for autonomy | (3) Transparency or understandability (4) Clear assumption of responsibility, accountability |
| Etienne et al.(85) | 2020 | France | To review applications of AI in thoracic surgery, including its potential and limitations, and discuss how it impacts diagnostics, prognostic decision-making, and surgical techniques. | N/A | Systematic review | AI demonstrates high accuracy in diagnosing lung nodules and aiding decision-making in thoracic surgery by evaluating individual risk factors. Challenges include the need for high-quality, representative datasets and improving algorithm generalizability across diverse populations. | 1.Beneficence | （1）Promotion of well-being/Personalized medicine |
| Feinstein et al.(86) | 2024 | United States | To explore future advancements in AI and remote monitoring technologies in anesthesiology, focusing on intraoperative, postoperative, and home care applications by 2050. | N/A | Narrative review | AI will integrate data streams to enhance intraoperative monitoring, automate routine tasks, and allow anesthesiologists to focus on complex decision-making. Wearable devices and remote monitoring will enable early detection of complications post-surgery. Regulatory and legal adaptations will be critical to enable these innovations. | 1.Beneficence 3.Justice | （1）Promotion of well-being （3）Clear assumption of responsibility, accountability |
| Fisher and Rosella(87) | 2022 | Canada | To identify priorities and strategies for implementing AI in public health to enhance equity, effectiveness, and efficiency across core public health functions. | N/A | Literature review | Key priorities include modernizing data governance, addressing workforce skills gaps, fostering partnerships, and ensuring equity and transparency in AI practices. AI offers significant potential for improving public health functions, but careful planning and equity-focused implementation are essential to avoid exacerbating disparities. | 1.Beneficence 2.Non-maleficence 3.Justice 4.Respect for autonomy | （1）Promotion of well-being （2）Privacy and data protection （3）Equity, fairness/Bias, discrimination （4）Transparency or understandability |
| Forghani(88) | 2023 | United States | To provide a structured approach for selecting and integrating AI algorithms in radiology departments to optimize clinical workflow, enhance diagnostic accuracy, and improve patient outcomes. | N/A | Literature review | Effective AI adoption requires assessing tools for clinical impact, operational efficiency, and workflow integration, considering metrics like diagnostic accuracy and return on investment. Barriers include poor generalizability, resource-intensive deployment, and lack of seamless integration. Multidisciplinary collaboration and robust deployment platforms are critical for success. | 1.Beneficence 2.Non-maleficence 3.Justice | （1）Accuracy and efficiency/Quality and safety of care （2）Need for human regulation; oversight （3）Bias, discrimination |
| Fox(89) | 2022 | Finland | To analyze behavioral ethics in human–AI systems using a behavioral ecology framework, focusing on function, evolution (phylogeny), mechanisms, and development (ontogeny) to identify ethical challenges and opportunities. | N/A | Conceptual analysis | Human–AI systems can reduce stress and improve decision-making under ideal conditions but may fail in complex or unpredictable environments. Continuous reliance on AI risks undermining human skills, requiring balanced integration. Robust design, individualized adaptations, and transparent policies are essential to ensure ethical and effective use. | 1.Beneficence 4.Respect for autonomy | （1）Promotion of well-being/Accuracy and efficiency （4）Informed consent/Transparency or understandability |
| Frehywot and Vovides(90) | 2023 | United States | To propose a framework for a transdisciplinary Community of Practice (CoP) to ensure AI is used equitably and sustainably in global health workforce training, addressing biases and structural inequities. | N/A | Editorial | AI should support rather than drives human decision-making in health contexts. The CoP model enables interdisciplinary collaboration to address challenges like algorithmic bias and promotes equitable AI integration into global health workforce training. Establishing governance structures, partnerships, and educational initiatives will ensure the framework's sustainability and inclusivity. | 3.Justice | （3）Equity, fairness/Bias, discrimination |
| Fuhrman et al.(91) | 2022 | United States | To review explainable and interpretable AI techniques in the context of medical imaging for COVID-19, highlighting their role in improving clinical decision-making and building trust in AI systems. | N/A | Literature review | Explainable AI techniques enhance the interpretability of COVID-19 diagnostic and prognostic models. Effective integration of explainable AI supports clinicians, but challenges remain in standardizing methods and addressing confounding features in imaging datasets. There is a need for robust evaluation metrics and transparent explainability practices. | 4.Respect for autonomy | （4）Transparency or understandability |
| Garg(92) | 2024 | India | To explore the potential and limitations of ChatGPT in revolutionizing mental health policy, focusing on its capabilities for policy analysis and the challenges of ethical, operational, and contextual integration. | N/A | Conceptual analysis | ChatGPT offers potential for enhancing mental health policy analysis but has significant limitations. Addressing these challenges requires robust ethical guidelines, collaboration among stakeholders, and advancements in algorithmic accountability. | 1.Beneficence 2.Non-maleficence 3.Justice 4.Respect for autonomy | （1）Accuracy and efficiency/Promotion of well-being （2）Privacy and data protection/Low data quality, accuracy and reliability concerns （3）Cultural diversity, sensitivity, and exclusivity/Equity, fairness/Bias, discrimination （4）Transparency or understandability |
| Gauss et al.(93) | 2023 | France | To review the application of ML in trauma science, focusing on its potential to enhance decision-making, predict outcomes, and improve patient care. | N/A | Literature review | ML holds promise in enhancing trauma care. Challenges include insufficient prospective studies, poor workflow integration, and limited understanding of human-ML interaction. Future ML systems should incorporate continuous physiological data, have enhanced causal inference, and integrate into clinical workflows while addressing ethical and legal concerns. | 1.Beneficence 2.Respect for autonomy 3.Justice 4.Non-maleficence | 1.Accuracy and efficiency/Personalized medicine/Clinician–patient relationship and communication 2.Transparency or understandability/Informed consent 3.Bias, discrimination 4.Privacy and data protection/Need for human regulation; oversight |
| Geraci et al.(94) | 2023 | Canada | To demonstrate the utility of ML in generating hypotheses for patient stratification and identifying therapeutic targets for ALS using small datasets within an Open Science framework. | 800 ALS patients and 100 healthy controls | ML-assisted quantitative descriptive analyses | ML identified eight target classes and novel genetic markers linked to ALS pathophysiology. Integration of ML and expert validation highlighted the potential of small datasets in rare disease research. | 1.Beneficence | （1）Personalized medicine |
| Goldstein et al.(95) | 2020 | United States | To elucidate the American Academy of Sleep Medicine's (AASM's) position on use of AI in analyzing electrophysiological data collected during polysomnography. | N/A | Position statement | AI is well-suited for analysis in polysomnography, in terms of enhancing efficiency and patient outcomes. However, various ethical, logistical, and legal issues remain to be addressed. | 1.Beneficence 2.Non-maleficence 4.Respect for autonomy | （1）Accuracy and efficiency/Personalized medicine/Clinical validity of algorithms （2）Privacy and data protection/Insufficient data volume, generalizability concerns/Need for human regulation; oversight （4）Transparency or understandability |
| Göndöcs and Dörfler(96) | 2024 | United Kingdom | To examine how dermatologists perceive and integrate AI in melanoma diagnosis and decision-making, focusing on AI’s role as a supportive tool rather than a replacement for human expertise. | 17 dermatologists | Qualitative interviews and thematic analysis | Dermatologists view AI as a tool, assistant, or colleague that enhances diagnostic accuracy and efficiency but requires clear explainability and trust-building. Responsibility for the final diagnosis remains firmly with physicians, emphasizing the necessity of human-AI collaboration. There is a need for robust training, explainability of algorithms, and real-world testing with active involvement of medical professionals in AI design. | 1.Beneficence 3.Justice 4.Respect for autonomy | （1）Accuracy and efficiency （3）Clear assumption of responsibility, accountability （4）Transparency or understandability |
| González-Gonzalo et al.(97) | 2022 | The Netherlands | To identify challenges along the trustworthy AI design pipeline in ophthalmology. | N/A | Editorial | Collaboration among stakeholders is needed for trustworthy AI design, in order to realize potential benefits of AI in ophthalmology. | 1.Beneficence 2.Non-maleficence 3.Justice 4.Respect for autonomy | （1）Clinician–patient relationship and communication （2）Privacy and data protection/Low data quality, accuracy and reliability concerns/Risk; error management/Need for human regulation; oversight/Misuse （3）Bias, discrimination/Clear assumption of responsibility, accountability （4）Informed consent/Transparency or understandability |
| Goodman(98) | 2020 | United States | To summarize evolving ethics and contemporary challenges of AI and ML in health. | N/A | Editorial | The tools of applied ethics are adequate to the task of guiding developers, users, and institutions as they adopt and attempt to make the most of health information technology. | 2.Non-maleficence 3.Justice 4.Respect for autonomy | （2）Privacy and data protection （3）Bias, discrimination （4）Transparency or understandability |
| Grote and Berens(99) | 2022 | Germany | To summarize epistemic and normative factors that may contribute to algorithmic overreliance in clinical decision making, and to identify requirements for bridging gaps between ML algorithms and clinicians. | N/A | Editorial | Inaccurate expectations, misestimation of uncertainties, lack of explainability, and factors of "sociotechnical context" may contribute to algorithmic overreliance in clinical decision making. Understanding of these factors can guide development of strategies to bridge gaps between algorithms and clinicians. | 2.Non-maleficence 3.Justice 4.Respect for autonomy | （2）Technology addiction, algorithmic overreliance/Risk; error management （3）Clear assumption of responsibility, accountability （4）Transparency or understandability |
| Guan(100) | 2019 | China | To discuss the potential benefits, ethical concerns, and governance of AI in healthcare. | N/A | Editorial | A global framework for ethical governance of AI in healthcare is needed. Important aspects to consider in such a framework include the role of government in oversight of AI and responsibilities of individual stakeholders in healthcare. | 1.Beneficence 2.Non-maleficence 3.Justice 4.Respect for autonomy | （1）Accuracy and efficiency/Clinical validity of algorithms/Promotion of well-being/Patient-centered care （2）Risk; error management/Privacy and data protection （3）Equity, fairness （4）Autonomy |
| Haines-Delmont et al.(101) | 2020 | United Kingdom | To evaluate the feasibility of applying ML to suicide risk prediction in acute mental healthcare, using data collected through smartphones. | 66 inpatients with acute mental health conditions | Quasi-experimental  (Participant risk level was compared upon entry and exit from inpatient ward) | Results from this study suggest that it is feasible to utilize smartphone user data and passive sensor data to generate a risk prediction algorithm among inpatients at risk for suicide. However, ethical and legal concerns related to this type of approach remain to be addressed. | 1.Beneficence | （1）Accuracy and efficiency/Personalized medicine/Promotion of well-being/Emotional support |
| Halm-Pozniak et al.(102) | 2023 | Germany | To explore the role of digital innovations, such as apps, wearables, robotics, AI, and telemedicine, in advancing orthopedic and trauma care while addressing challenges like ethics, data privacy, and integration into clinical workflows. | N/A | Narrative review | Digital tools improve diagnostic precision, surgical accuracy, and patient monitoring but require ethical oversight and transparent data governance. Apps and wearables enhance patient engagement and remote monitoring, while AI aids in imaging and decision support. Challenges include integration, cost, and workflow adaptability. Collaboration among stakeholders and adherence to ethical frameworks are essential. | 1.Beneficence 2.Non-maleficence 3.Justice 4.Respect for autonomy | （1）Clinician–patient relationship and communication/Personalized medicine/Accuracy and efficiency/Promotion of well-being/Quality and safety of care （2）Privacy and data protection （3）Equity, fairness/Bias, discrimination （4）Transparency or understandability |
| Hammouda and Neyra(103) | 2022 | United States | To review literature on the utilization of AI in continuous renal replacement therapy (CRRT), highlighting current evidence gaps. | 10 articles | Scoping review | Primary research interests in the studies in this review included early indicators of need for CRRT, prognostication of mortality and kidney recovery, and risk factors for mortality. Secondary research interests included dynamic CRRT monitoring, prediction of CRRT complications, and point-of-care analysis using automated data pooling. Under-researched areas included prospective validation, implementation barriers, ascertainment of biases, and measurement of AI-related healthcare disparities. | 1.Beneficence； 3.Justice | （1）Accuracy and efficiency  （3）Bias, discrimination/Equity, fairness |
| Hane and Wasserman(104) | 2022 | United States | To describe pragmatic tools that can support more effective use of risk scores for equitable healthcare outreach programs. | A longitudinal dataset including 1,511,260 members of a commercial or Medicare Advantage plan from the Optum Labs Data Warehouse | Quasi-experimental  (Authors fit model to training data and then evaluated bias in risk scores by comparing predicted outcomes to true outcomes) | The model-based approach developed in this study could be used to optimize equitable selection of risk thresholds for targeted healthcare outreach. | 2.Non-maleficence 3.Justice 4.Respect for autonomy | （2）Risk; error management （3）Equity, fairness （4）Shared decision-making |
| Hariharan et al.(105) | 2023 | United States | To explore how ML can improve spinal cord stimulation (SCS) outcomes for chronic pain patients through enhanced candidate selection, preoperative screening, and programming optimization. | N/A | Narrative review | ML improves candidate selection for SCS, achieving predictive accuracies up to 96%, and supports real-time optimization of stimulation parameters in closed-loop systems. Further large-scale validation is required. | 1.Beneficence 4.Respect for autonomy | （1）Personalized medicine/Promotion of well-being （4）Shared decision-making |
| Harrer(106) | 2023 | Australia | To describe how generative AI, especially Large Language Models (LLMs), could transform data management workflows in healthcare; to explain how the underlying technology works; to provide an assessment of risks and limitations of LLMs; and to propose a framework for responsible design and use of LLMs. | N/A | Editorial | Limitations of LLMs are systemic; boosting training data sizes and numbers of model parameters will amplify rather than overcome limitations. Responsible design and use of LLMs should focus on accountability, fairness, data privacy, transparency, explainability, and alignment of value and purpose. | 1.Beneficence 2.Non-maleficence 3.Justice 4.Respect for autonomy | （1）Accuracy and efficiency/Clinical validity of algorithms/Clinician–patient relationship and communication/Promotion of well-being； （2）Misinformation/Privacy and data protection （3）Cultural diversity, sensitivity, and exclusivity （4）Transparency or understandability |
| Harutyunyan et al.(107) | 2024 | Canada | To explore the evolving role of ultrasound (U/S) in anesthesia, focusing on its integration with AI, robotics, and advanced imaging technologies to enhance patient care and educational practices. | N/A | Narrative review | AI-enabled U/S enhances precision in image interpretation and guides complex procedures, reducing errors and improving outcomes. Integration of robotics and augmented reality with U/S promises less invasive procedures, remote capabilities, and real-time visualization for education and surgical planning. | 1.Beneficence | （1）Accuracy and efficiency/Quality and safety of care/Promotion of well-being/Clinical validity of algorithms |
| Haselager et al.(108) | 2023 | The Netherlands | To propose “reflection machines” (RMs) as an approach to maintaining meaningful human control in decision support systems (DSSs). | N/A | Editorial | Use of DSSs is growing, and there is a need for effective human oversight. RMs could help maintain human oversight and mitigate harms of over-relying on DSSs. | 1.Beneficence 3.Justice 4.Respect for autonomy | （1）Clinical validity of algorithms/Clinician–patient relationship and communication/Promotion of well-being （3）Accessibility of services （4）Shared decision-making/Autonomy |
| Hazarika(109) | 2020 | Philippines | To identify opportunities for AI to potentially transform healthcare providers' roles. | N/A | Literature review | AI introduces new challenges for policymakers, industry, healthcare providers, and patients to address. To optimize the benefits of AI, a balance will need to be struck between facilitating innovation and maintaining transparency and accountability. | 1.Beneficence 2.Non-maleficence 3.Justice 4.Respect for autonomy | （1）Promotion of well-being/Clinician–patient relationship and communication/Accuracy and efficiency/Clinical validity of algorithms/Patient-centered care （2）Risk; error management/Trust in implementing AI for healthcare/Privacy and data protection （3）Clear assumption of responsibility, accountability （4）Autonomy |
| Hesse and Shneiderman(110) | 2007 | United States | To combine evidence from various fields, including human-computer interaction, to suggest improvements to the quality of medical care. | N/A | Editorial | Evidence-based medicine must be merged with user-centered research to enhance quality of life equitably through medicine and prevent a crisis in healthcare from occurring in the wake of expanding eHealth. | 1.Beneficence 3.Justice | （1）Patient-centered care （3）Equity, fairness |
| Hine et al.(111) | 2022 | United Kingdom | To explore ethical issues in smart care systems for people living with dementia and the extent to which system design can address ethical issues. | N/A | Editorial | Developing ethical smart care systems for people living with dementia requires consideration of multiple ethical frameworks. There are many ways to embed ethical principles into design. | 2.Non-maleficence 3.Justice 4.Respect for autonomy | （2）Privacy and data protection （3）Bias, discrimination/Equity, fairness （4）Autonomy |
| Holderried et al.(112) | 2024 | Germany | To evaluate the feasibility and usability of a GPT-powered chatbot as a simulated patient for practicing medical history-taking skills. | 28 medical students | Mixed methods quasi-experimental study  (Students used the GPT chatbot to simulate patient interactions, compared to traditional methods of learning (implied through historical use of real or simulated patients). Outcomes included plausibility of chatbot responses and usability scores.) | The chatbot provided 97.9% plausible answers, effectively simulating a patient for history-taking, though occasional errors reflected socially desirable rather than medically accurate responses. Students rated the chatbot experience positively (77/100 on usability). | 1.Beneficence | （1）Promotion of well-being/Clinician–patient relationship and communication/Clinical validity of algorithms/Patient-centered care |
| Howard(113) | 2019 | United States | To discuss origins of AI and ML, emerging AI applications, and implications for future work arising from use of AI applications. | N/A | Editorial | There is uncertainty about how AI will shape the future of work and human roles in work. As AI applications enter workplaces, attention is needed from occupational safety, healthcare provider, employer, and worker perspectives to address challenges concerning worker safety and well-being. | 2.Non-maleficence 4.Respect for autonomy | （2）Privacy and data protection （4）Transparency or understandability |
| Howard et al.(114) | 2024 | United Kingdom | To present a framework for implementing AI-driven antimicrobial learning systems (ALSs) to address antimicrobial resistance (AMR), focusing on adaptable, scalable, and sustainable solutions. | N/A | Conceptual analysis | AMR-targeted AI models can improve clinical decision support. Effective deployment requires overcoming barriers like data sparsity, regulatory hurdles, algorithmic biases, and integration challenges. Scalability and sustainability depend on adopting open-source frameworks, enabling global collaboration, and integrating One Health data. | 2.Non-maleficence 3.Justice | （2）Privacy and data protection （3）Bias, discrimination |
| Howard et al.(115) | 2024 | United Kingdom | To propose a mathematically grounded, outcome-based model to quantify antimicrobial treatment imprecision and develop learning antimicrobial systems for improving precision in healthcare policy and practice. | N/A | Conceptual analysis | AI-driven imprecision modeling and learning antimicrobial systems can optimize therapeutic outcomes. Ethical, regulatory, and interdisciplinary collaborations are essential. | 1.Beneficence 4.Respect for autonomy | （1）Accuracy and efficiency （4）Transparency or understandability/Shared decision-making/Informed consent |
| Hua et al.(116) | 2024 | Australia | To investigate factors influencing the acceptability of AI in medical imaging among healthcare professionals and provide insights into strategies for improving its adoption and integration. | 31 studies | Scoping review | Key factors affecting AI acceptability include user trust, AI literacy, workflow integration, perceived value, and concerns about ethicality and professional roles. Studies emphasize the need for human-centered AI systems designed with healthcare-specific contexts in mind. Future research should focus on real-world settings, explainable AI, and the role of education in improving AI literacy to bridge gaps in adoption and maximize value in clinical workflows. | 1.Beneficence | （1）Quality and safety of care/Personalized medicine/Dignity, empathy, and humanism in healthcare |
| Isbanner et al.(117) | 2022 | Australia | To assess Australians' attitudes regarding use of AI across various healthcare and social service applications and to determine which characteristics of healthcare and social service AI systems Australians consider most important. | 1,950 Australians, in addition to a further 2,498 responses for a subset of questions | Cross-sectional quantitative study | Australians support use of AI generally, but their support declines when asked about specific scenarios and potential harms versus benefits. Participants considered accuracy to be the most important characteristic of AI systems and indicated that they would prefer for AI systems to augment rather than replace humans in healthcare and social services. | 3.Justice | （3）Clear assumption of responsibility, accountability/Equity, fairness |
| Jansson et al.(118) | 2022 | Finland | To assess content validity of an AI-enhanced system for care pathway planning and scheduling. | 50 clinical professionals from 3 European countries | Cross-sectional quantitative study | The highest ranked functionalities for the proposed AI system were related to risk assessment, patient profiling, and resources. Explainability of ML models was ranked highest for the user interface. Results on relevance scores suggested that there may be a low level of organizational readiness for AI in healthcare. | 4.Respect for autonomy | （4）Transparency or understandability |
| Jeyaraman et al.(119) | 2023 | India | To evaluate the potential ethical challenges of ChatGPT in healthcare, education, and research, emphasizing the balance between its benefits and the necessity of human oversight. | N/A | Literature review | ChatGPT enhances workflows, decision-making, and personalized learning but requires stringent oversight to mitigate risks. Integration demands clear guidelines, interdisciplinary collaboration, and public awareness. Continuous research and proactive policy-making are vital to address concerns. | 1.Beneficence 2.Non-maleficence 3.Justice 4.Respect for autonomy | （1）Personalized medicine/Clinician–patient relationship and communication （2）Privacy and data protection （3）Bias, discrimination （4）Transparency or understandability |
| Joda and Zitzmann(120) | 2022 | Switzerland | To describe personalized workflows in reconstructive dentistry and highlight the disruptive potential of new technologies. | N/A | Literature review | AI can be expected to increase diagnostic accuracy, simplify treatment planning, and contribute to development of personalized reconstructive workflows. | 3.Justice | （3）Clear assumption of responsibility, accountability |
| Jones(121) | 2017 | United States | To examine the role of dignity in data protection laws related to automated decision making | N/A | Editorial | There are two different legal constructions related to automated data processing. One has emphasized a right to "human in the loop," in order to protect human dignity. The other has supported full automation based on the perceived fairness and objectivity of computers. | 2.Non-maleficence | （2）Privacy and data protection |
| Joyce et al.(122) | 2023 | England | To describe the TIFU (Transparency and Interpretability For Understandability) framework and examine how it applies to AI/ML in mental health research. | N/A | Literature review | Human understandability of inputs is essential to trustworthy AI/ML systems in psychiatry. | 4.Respect for autonomy | （4）Transparency or understandability |
| Kellmeyer(123) | 2019 | Germany | To discuss scientific opportunities and ethical challenges of AI in basic and clinical neuroscience. | N/A | Editorial | AI in neuroscience is likely to bring groundbreaking progress as well as a need for increased attention and educational curricula around neuro-ethics. | 2.Non-maleficence 3.Justice 4.Respect for autonomy | （2）Privacy and data protection （3）Bias, discrimination/Clear assumption of responsibility, accountability /Equity, fairness （4）Autonomy |
| Kim et al.(124) | 2023 | Republic of Korea | To critically examine metaverse wearables and their potential applications in immersive digital healthcare, particularly in areas such as rehabilitation, medical education, remote patient management, and surgical training. | N/A | Literature review | Metaverse wearables enhance healthcare outcomes. Challenges include data privacy, technology costs, and long-term power supply. Future advancements in wearable interfaces and brain-computer integration could further revolutionize patient care, especially for elderly and chronic disease populations. | 1.Beneficence 3.Justice | （1）Quality and safety of care （3）Equity, fairness / Bias, discrimination |
| Kirtley et al.(125) | 2022 | United Kingdom | To synthesize empirical and review literature on ML in electronic health records for suicide research and to highlight critical matters related to ML in clinical practice. | N/A | Literature review | ML has a potential role in suicide prevention but is not yet ready to be implemented in practice. | 1.Beneficence 3.Justice 4.Respect for autonomy | （1）Clinical validity of algorithms （3）Bias, discrimination （4）Transparency or understandability |
| Koski and Murphy(126) | 2021 | United States | To describe the origins, building blocks, and challenges of AI applications in healthcare. | N/A | Editorial | AI applications in healthcare can improve precision, reduce disparities, accelerate discovery, and empower patients. | 1.Beneficence 2.Non-maleficence 3.Justice 4.Respect for autonomy | （1）Accuracy and efficiency/Quality and safety of care （2）Low data quality, accuracy and reliability concerns （3）Equity, fairness （4）Transparency or understandability |
| Kremer et al.(127) | 2023 | United States | To evaluate the role of immersive digital training technologies, such as virtual reality, in enhancing patient safety by improving the decontamination process for reusable medical devices. | N/A | Narrative review | Immersive technologies like VR provide interactive training to ensure proper cleaning and sterilization of medical devices. AI and extended reality tools can enhance competency, sustainability, and safety. Digital twins and automation further optimize workflows, reduce errors, and support sustainable practices in medical device reprocessing. | 2.Non-maleficence 4.Respect for autonomy | （2）Low data quality, accuracy and reliability concerns （4）Transparency or understandability |
| Kretzschmar et al.(128) | 2019 | United Kingdom | To discuss strengths and limitations of using chatbots for mental health support, from a young person's perspective, and to outline ethical standards for these platforms. | N/A | Editorial | Young people may rely more and more on digital resources for mental health support. There is a need to attend to ethical standards around these platforms and to fund research and professional services for mental health support. | 1.Beneficence 2.Non-maleficence 4.Respect for autonomy | （1）Quality and safety of care/Personalized medicine （2）Privacy and data protection/Technology addiction，algorithmic overreliance （4）Informed consent/Transparency or understandability |
| Kudina(129) | 2021 | The Netherlands | To examine challenges that are bypassed by the European Union's proposed Artificial Intelligence Act. | N/A | Editorial | Due to the use of AI in healthcare, there is an urgent need to address questions around co-diagnosis between human and AI agents, new clinician and patient responsibilities, and media literacy. | 1.Beneficence 3.Justice 4.Respect for autonomy | （1）Clinician–patient relationship and communication （3）Bias, discrimination/Clear assumption of responsibility, accountability/Equity, fairness/Affordability and availability （4）Informed consent |
| Lakey et al.(130) | 2024 | United States | To explore the use of ML and AI for early prediction and prognosis of Type 2 Diabetes (T2D) by identifying and validating predictive biomarkers. | N/A | Literature review | ML algorithms can integrate multiomics data to identify causal biomarkers, offering improved accuracy and predictive ability for early T2D prognosis. Combining AI with advanced bioinformatics enables personalized and precise interventions, addressing disease heterogeneity and supporting preventive healthcare strategies. Challenges remain in data validation, standardization, and ensuring applicability across diverse populations. | 1.Beneficence | （1）Quality and safety of care |
| Lalmuanawma et al.(131) | 2020 | India | To describe the role of AI and ML as significant methods in screening, predicting, forecasting, contact tracing, and drug development for COVID-19. | N/A | Literature review | AI and ML significantly improved treatment, screening, prediction, contact tracing, and vaccine development for the COVID-19 pandemic. AI and ML have not been deployed widely enough to comment on their efficacy for other healthcare applications. | 1.Beneficence 2.Non-maleficence | （1）Accuracy and efficiency （2）Privacy and data protection |
| Lam et al.(132) | 2022 | United Kingdom | To define "digital surgery" and its surrounding ethical issues, and to identify barriers and research goals for clinical practice. | 38 international experts across the domains of surgery, industry, AI, ethics, law, and policy. | Qualitative Delphi panel | Key ethical issue in digital surgery includes data privacy, confidentiality, public trust, consent, and liability. | 2.Non-maleficence 3.Justice 4.Respect for autonomy | （2）Insufficient data volume, generalizability concerns/Privacy and data protection/Need for human regulation; oversight （3）Clear assumption of responsibility, accountability （4）Informed consent |
| Lamanna(133) | 2021 | Australia | To outline a model that combines human task-sharing with human-AI cooperation for patient care in Sub-Saharan Africa, and to highlight potential benefits, risks, and ethical questions. | N/A | Editorial | AI and healthcare providers can work as a team, with strong potential to improve healthcare in Sub-Saharan Africa. | 1.Beneficence 2.Non-maleficence | （1）Accuracy and efficiency  （2）Low data quality, accuracy and reliability concerns |
| Lee and Yoon(134) | 2021 | Korea | To explore the current state and impact of AI in healthcare and to analyze several real-world examples of AI in healthcare. | N/A | Literature review | AI will likely health healthcare providers improve the efficiency and quality of their care. Integrating AI into healthcare in a way that maximizes benefits will have to involve effective planning and large-scale changes to healthcare operations. | 1.Beneficence 2.Non-maleficence 3.Justice 4.Respect for autonomy | （1）Accuracy and efficiency/Quality and safety of care/Clinician–patient relationship and communication/Clinical validity of algorithms/Promotion of well-being/Patient-centered care （2） Privacy and data protection （3）Clear assumption of responsibility, accountability （4）Shared decision-making |
| Li et al.(135) | 2024 | China | To highlight challenges related to use of AI in the field of infectious diseases and propose strategies for mitigating challenges. | N/A | Literature review | Effectiveness of AI, including in the domain of infectious diseases, depends heavily on data availability. Balancing the need for open data and the ethical imperative to protect individuals' privacy requires careful consideration. | 2.Non-maleficence | （2）Privacy and data protection |
| Li et al.(136) | 2023 | Singapore | To synthesize evidence on effectiveness of AI-based conversational agents (CAs) in mental health care. | 35 studies | Systematic review | In the studies included in this review, AI-based Cas significantly reduced depression and distress, but CA-based interventions showed no effects on overall psychological well-being. Further research is needed to understand mechanisms of AI-based CA effectiveness and safe integration in mental healthcare. | 1.Beneficence 2.Non-maleficence | （1）Promotion of well-being （2）Privacy and data protection/Misinformation |
| Li et al.(137) | 2023 | United States | To identify barriers to effective implementation of AI in healthcare and strategies to overcome barriers. | 306 articles | Systematic review | There are barriers to effective implementation of AI in healthcare at three levels: technical/algorithm, stakeholder, and social. Further research is needed to identify barriers that may be particular to pediatric care and to develop clear protocols for overcoming barriers. | 1.Beneficence 2.Non-maleficence 3.Justice 4.Respect for autonomy | （1）Promotion of well-being/Clinician–patient relationship and communication （2）Privacy and data protection （3）Clear assumption of responsibility, accountability/Bias, discrimination/Equity, fairness （4）Transparency or understandability/Informed consent |
| Li et al.(138) | 2023 | China | To summarize prospective applications of GPT-4 in neurosurgery, taking moral considerations into account. | N/A | Editorial | GPT-4 is unlikely to replace neurosurgeons but has potential to augment precision and effectiveness of procedures. | 1.Beneficence 2.Non-maleficence 3.Justice 4.Respect for autonomy | （1）Accuracy and efficiency/Promotion of well-being （2）Privacy and data protection （3）Clear assumption of responsibility, accountability （4）Autonomy |
| Li et al.(139) | 2023 | China | To explore how AI may influence practice of Traditional Chinese Medicine (TCM). | N/A | Editorial | While AI may enhance efficacy of research on TCM, the role of the human practitioner is irreplaceable. Further legal regulations are needed to ensure appropriate accountability, safety, and quality in AI-enhanced TCM. | 1.Beneficence 3.Justice | （1）Dignity, empathy, and humanism in healthcare/Clinician–patient relationship and communication/Accuracy and efficiency/Personalized medicine （3）Equity, fairness/Clear assumption of responsibility, accountability |
| Li et al.(140) | 2023 | China | To assess how human-robot interaction could be integrated into rehabilitation training following stroke. | 10 rehabilitation training patients   (Model classification accuracy was compared across 10 patients who participated in online and offline protocols.) | Quasi-experimental study | The model tested in this study showed potential to be applied effectively in clinical upper limb rehabilitation training. | 1.Beneficence | （1）Accuracy and efficiency/Clinical validity of algorithms |
| Lin et al.(141) | 2019 | United States | To describe the top ten ways that AI might influence primary care, from the perspective of primary care physicians. | N/A | Editorial | AI holds great promise for freeing up physicians' cognitive and emotional space for patient care and increasing personalization of patient care. If implemented poorly, AI risks displacing humanity. It will be critical to determine specific boundaries of where AI strengthens versus undermines healing. | 1.Beneficence 4.Respect for autonomy | （1）Quality and safety of care/Accuracy and efficiency/Promotion of well-being/Clinical validity of algorithms/Patient-centered care （4）Patients' health-related knowledge |
| Liu and Xiao(142) | 2021 | United States | To describe the importance and challenges of improving public eHealth literacy around communicable and non-communicable diseases, and to suggest methods of improving eHealth literacy using AI. | N/A | Editorial | AI-based approaches to improving eHealth literacy have the advantage of matching suitable information to individual information needs. | 1.Beneficence 4.Respect for autonomy | （1）Patient-centered care （4）Patients' health-related knowledge |
| Liyanage et al.(143) | 2019 | United Kingdom | To form expert consensus around perceptions, issues, and challenges of AI in primary care. | A panel of 20 participants consisting of primary care clinicians and health informatics experts. | Qualitative Delphi panel | Experts believe AI has potential to improve clinical and administrative decisions and processes, which could be further facilitated by common data standards. Agreement was not reached around whether AI should adapt to clinician preferences and behavior, or around the extent to which AI poses harm to patients. | 1.Beneficence | （1）Clinical validity of algorithms |
| Luu et al.(144) | 2024 | France | To describe challenges that must be addressed if AI is to be incorporated into precision oncology practice. | N/A | Editorial | Developing public-private partnerships can help to ensure that AI complies with ethical and regulatory requirements and also increase data availability. | 2.Non-maleficence 4.Respect for autonomy | （2）Privacy and data protection （4）Transparency or understandability |
| Lynn(145) | 2019 | United States | To describe the present state of acute care decision making, the potential integration of AI into acute care and its hazards and challenges, and to describe the role that clinicians must continue to play as overseers of care. | N/A | Editorial | Functional cooperation between human and AI offers the greatest hope for a safe transformation of acute care. Early collaboration by human experts across diverse fields is needed to achieve this goal. | 2.Non-maleficence 4.Respect for autonomy | （2）Risk; error management （4）Transparency or understandability |
| Maidhof et al.(146) | 2023 | Germany | To investigate future users' acceptance and benefit-barrier perception of video-based ambient assisted living (AAL) technologies. | 146 adult participants from Germany and Bulgaria | Cross-sectional quantitative study | Acceptance and perceived benefits of AAL were higher for lower-privacy activities of daily living and lower for activities that could be considered more private or intimate. | 1.Beneficence 2.Non-maleficence | （1）Quality and safety of care （2）Privacy and data protection/Misuse |
| Malerbi et al.(147) | 2023 | Brazil | To emphasize the importance of teaching healthcare providers about digital healthcare, in order to realize the benefits and safe deployment of AI in healthcare. | N/A | Editorial | Healthcare trainees should be taught fundamental AI concepts. Digital competencies could also be taught to licensed healthcare providers through continued education, conferences, and datathon or hackathon events. It is also essential to familiarize patients with the benefits and limitations of AI in health care to gain their trust and support. | 2.Non-maleficence | （2）Trust in implementing AI for healthcare |
| Matheny et al.(148) | 2020 | United States | To discuss the introduction of AI into healthcare, issues to prioritize within the integration of AI and healthcare, and factors of current systems that need improvement in order to achieve successful integration. | N/A | Editorial | Promoting population-representative data with accessibility, standardization, and quality is imperative. It is also important to prioritize ethical, equitable, and inclusive healthcare AI through training programs and frameworks for best practices. | 2.Non-maleficence 3.Justice 4.Respect for autonomy | （2）Privacy and data protection （3）Bias, discrimination （4）Transparency or understandability |
| Mathiesen and Broekman(149) | 2022 | Denmark | To discuss ethical challenges that must be addressed in the context of ML-assisted medical decision making. | N/A | Editorial | Challenges such as lack of guaranteed privacy, risk of unjust resource allocation, and lack of ethical accountability must be addressed to support realization of the potential benefits of ML and AI in medical decision making. | 2.Non-maleficence 3.Justice 4.Respect for autonomy | （2）Privacy and data protection （3）Equity, fairness/Bias, discrimination （4）Transparency or understandability |
| McHugh and Pai(150) | 2023 | United States | To summarize how AI has potential to enhance pathologic reporting on colorectal cancer and identify barriers to implementation. | N/A | Editorial | AI has potential to provide rapid prediction of key features influencing patient outcomes in pathologic reporting for colorectal cancer. Barriers to implementation include costs of slide digitization and algorithm development. | 4.Respect for autonomy | （4）Transparency or understandability |
| Mese(151) | 2024 | Turkey | To explore the benefits of combining virtual reality and augmented reality (VR-AR) tools to provide accurate healthcare information in a more digestible format. | N/A | Editorial | Integrating ChatGPT with VR-AR technologies can revolutionize healthcare and provide at-home access to accurate, easily comprehensible information. However, issues such as data security and privacy should also be considered. | 1.Beneficence 2.Non-maleficence | （1）Accuracy and efficiency/Quality and safety of care/Clinician–patient relationship and communication （2）Privacy and data protection |
| Mese et al.(152) | 2023 | Turkey | To discuss the potential benefits, limitations, and ethical concerns related to integrating ChatGPT into radiology workflows. | N/A | Editorial | ChatGPT can automate repetitive tasks, improve communication, and enhance accuracy of diagnosis. However, ethical concerns including bias, privacy, and source credibility remain to be addressed. | 1.Beneficence 2.Non-maleficence 3.Justice 4.Respect for autonomy | （1）Accuracy and efficiency/Clinician–patient relationship and communication/Dignity, empathy, and humanism in healthcare/Personalized medicine/Clinical validity of algorithms/Promotion of well-being （2）Misinformation/Low data quality, accuracy and reliability concerns/Privacy and data protection （3）Bias, discrimination （4）Transparency or understandability |
| Meskó(153) | 2023 | Hungary | To present the implications of generative AI that uses LLMs and is able to process multimodal inputs and how these relate to the future of AI in healthcare. | N/A | Editorial | M-LLMs can process multiple types of media including video, image, and sound, further amplifying their potential use in healthcare for things like patient interactions, image analyses, and more. M-LLMs can also connect multimodal medias in medicine such as radiology AI models and electronic medical records. However, there are still many barriers that keep generative AI from being a sufficient replacement for human care. | 1.Beneficence 2.Non-maleficence 3.Justice 4.Respect for autonomy | （1）Accuracy and efficiency/Patient-centered care/Clinician–patient relationship and communication/Dignity, empathy, and humanism in healthcare/Personalized medicine （2）Privacy and data protection/Need for human regulation; oversight （3）Cultural diversity, sensitivity, and exclusivity （4）Transparency or understandability |
| Metsch et al.(154) | 2024 | Germany | To create the explainable AI platform, CLARUS, which allows physicians to further understand the graph neural networks behind and factors used in AI decisions in healthcare. | Training datasets downloaded from The Cancer Genome Atlas (TCGA) database, containing molecular and genetic profiles for over 33 different cancer types from 20,000 individual tumor samples | Quantitative descriptive analysis | CLARUS gives explainable AI causability and allows users to better understand how to generate certain predictions using the Graph Neural Network by manipulating variables themselves. | 4.Respect for autonomy | （4）Transparency or understandability |
| Michelson et al.(155) | 2022 | United States | To discuss the potential for ML in developing prediction models to improve care in the pediatric intensive care unit (PICU), as well as related ethical concerns and organizational and legal challenges. | N/A | Editorial | While ML models can efficiently incorporate large datasets, accumulate data while self-adjusting, and enhance the accuracy of predictions there are also several challenges to their implementation. Challenges include biases, lack of diversity/inaccuracies in the datasets themselves, data privacy concerns, and liability concerns. | 1.Beneficence 2.Non-maleficence 3.Justice 4.Respect for autonomy | （1）Accuracy and efficiency/Quality and safety of care/Clinician–patient relationship and communication （2）Trust in implementing AI for healthcare/Privacy and data protection （3）Bias, discrimination （4）Transparency or understandability/Informed consent |
| Mollura et al.(156) | 2020 | United States | To discuss the potential for AI to be used in radiology in low- and middle-income countries. | N/A | Editorial | AI can bring significant benefits to resource-poor health institutions but requires thoughtful and collaborative implementation. The introduction of AI into radiology in low- and middle-income countries will look different from high-income countries because of marked differences in personnel, clinical experience, disease patterns, demographics, and radiology equipment. | 2.Non-maleficence 3.Justice 4.Respect for autonomy | （2）Trust in implementing AI for healthcare/Privacy and data protection/Need for human regulation; oversight （3）Equity, fairness （4）Transparency or understandability |
| Monteith et al.(157) | 2022 | United States | To discuss challenges to successful implementation AI in psychiatry. | N/A | Editorial | Challenges of AI in psychiatry include maturity of AI technology, physician attitudes and knowledge around technology, workflow changes, need for ongoing organizational support, patient safety issues, and problems unique to treating mental illness. Solutions will take time to discover, validate, and implement. | 2.Non-maleficence 3.Justice 4.Respect for autonomy | （2）Low data quality, accuracy and reliability concerns （3）Bias, discrimination （4）Transparency or understandability |
| Mun et al.(158) | 2024 | Republic of Korea | To present a model, U-AnoGAN to address AI challenges, including its black box qualities and data imbalance, and increase accuracy of Clinical Decision Support Systems. | Two different X-ray medical datasets  (U-AnoGAN was compared to two existing Clinical Decision Support Systems; outcome: anomaly scores for Covid, pneumonia, and normal datasets.) | Quasi-experimental | After testing, U-AnoGAN proved to be better than other CDSS models and solved the data imbalance and black box issues. U-AnoGAN could have significant potential for bolstering patient care with AI decision making. | 2.Non-maleficence 4.Respect for autonomy | （2）Insufficient data volume, generalizability concerns （4）Transparency or understandability |
| Murdoch(159) | 2021 | Canada | To examine privacy concerns related to commercial healthcare AI. | N/A | Editorial | Regulation and oversight are falling behind the pace of implementation of AI in healthcare. Increased regulation is needed around patient agency and consent, as well as data protection. | 2.Non-maleficence 4.Respect for autonomy | （2）Privacy and data protection/Risk; error management （4）Transparency or understandability |
| Muthuraj and Singla(160) | 2023 | India | To describe fundamental concepts, techniques, and applications of AI and ML. | N/A | Editorial |  | 2.Non-maleficence 3.Justice 4.Respect for autonomy | （2）Low data quality, accuracy and reliability concerns/Privacy and data protection （3）Bias, discrimination （4）Transparency or understandability |
| Nakagawa et al.(161) | 2023 | United States | To describe technological, clinical, sociological, and legal factors that will influence the adoption of AI in pathology, and to discuss the potential impact of this technology. | N/A | Editorial | Much of the potential impact of AI in pathology remains unknown. AI may increase the efficiency of practice but may also cause deskilling, "dethrilling," and burnout among clinicians. | 2.Non-maleficence 3.Justice | （2）Low data quality, accuracy and reliability concerns （3）Clear assumption of responsibility, accountability/Bias, discrimination/Affordability and availability |
| Nazir and Wang(162) | 2023 | United States | To provide an overview of ChatGPT uses and challenges. | N/A | Editorial | ChatGPT still faces many limits including risk of misinformation, privacy issues, training necessities, biased outputs, and limited contextual understanding. However, with future advancements, language modeling technologies can be used to augment almost any human-computer interaction. | 1.Beneficence 2.Non-maleficence 3.Justice 4.Respect for autonomy | （1）Promotion of well-being/Clinical validity of algorithms/Accuracy and efficiency/Quality and safety of care/Clinician–patient relationship and communication （2）Misinformation/Privacy and data protection/Misuse （3）Bias, discrimination/Clear assumption of responsibility, accountability/Cultural diversity, sensitivity, and exclusivity （4）Transparency or understandability/Informed consent |
| Niel and Bastard(163) | 2019 | France | To provide an overview of AI in nephrology that is relevant to practicing nephrologists. | N/A | Editorial | AI opens up new opportunities for patients and clinicians but also comes with ethical, training, regulatory, and liability challenges. There is a need for high-quality research to validate AI algorithms and for continuous monitoring of algorithm performance. | 1.Beneficence 2.Non-maleficence 4.Respect for autonomy | （1）Accuracy and efficiency/Clinical validity of algorithms/Clinician–patient relationship and communication （2）Insufficient data volume, generalizability concerns/Privacy and data protection （4）Transparency or understandability |
| Ostberg et al.(164) | 2021 | United States | To describe how advances in ML may influence the future of thoracic surgery. | N/A | Literature review | ML is likely to augment surgical performance and patient outcomes. To facilitate this process, surgeons need to have a strong understanding of ML techniques, current applications of ML in surgery, and shortcomings of ML. | 1.Beneficence 2.Non-maleficence 3.Justice 4.Respect for autonomy | （1）Accuracy and efficiency/Clinical validity of algorithms/Personalized medicine/Clinician–patient relationship and communication （2）Risk; error management/Low data quality, accuracy and reliability concerns/Need for human regulation; oversight （3）Bias, discrimination/Equity, fairness （4）Transparency or understandability/Informed consent |
| Ostherr(165) | 2022 | United States | To suggest future directions for humanities research on healthcare AI. | N/A | Editorial | AI is likely to yield new medical knowledge and better patient outcomes. Potential harms include algorithmic bias and dehumanization of healthcare. Humanities research can contribute expertise on the language of "health" and "medicine," social determinants of health, narrative medicine, technological medication, and discussions on disability and other aspects of identity in technology development. | 1.Beneficence 2.Non-maleficence 3.Justice 4.Respect for autonomy | （1）Accuracy and efficiency/Patient-centered care/Clinician–patient relationship and communication/Clinical validity of algorithms （2）Need for human regulation oversight/Privacy and data protection （3）Equity, fairness/Bias, discrimination （4）Autonomy/Informed consent |
| Ott and Dabrock(166) | 2022 | Germany | To discuss the concept of transparency for both humans and AI, and the assumptions on which the concept is based. | N/A | Editorial | Transparency is an intrathecal concept, rather than an ethical principle per se. Related concepts that are important to consider in realizing transparency include intelligibility, data sovereignty, and Open Data. | 4.Respect for autonomy | （4）Transparency or understandability |
| Ou et al.(167) | 2021 | United States | To describe current applications of deep learning methods within breast radiology. | N/A | Literature review | Diagnostic capabilities of deep learning in breast radiology are continuing to improve. Challenges that remain to be addressed include limited availability of high-quality data, potential for overreliance on algorithms, and automation bias. | 1.Beneficence 2.Non-maleficence 3.Justice 4.Respect for autonomy | （1）Accuracy and efficiency  （2）Low data quality, accuracy and reliability concerns/Trust in implementing AI for healthcare （3）Bias, discrimination （4）Transparency or understandability |
| Pagliari(168) | 2021 | United Kingdom | To discuss digital developments in primary care over time and the significant changes prompted by the COVID-19 pandemic. | N/A | Editorial | While the pandemic resulted in a "digital big band" for primary care, the most likely approach to creating fruitful change in the longer term will be an incremental strategy focused on augmenting rather than replacing existing services. | 1.Beneficence 2.Non-maleficence 3.Justice 4.Respect for autonomy | （1）Accuracy and efficiency/Dignity, empathy, and humanism in healthcare/ Personalized medicine （2）Privacy and data protection/Risk; error management/Trust in implementing AI for healthcare （3）Cultural diversity, sensitivity, and exclusivity / Bias, discrimination / Equity, fairness （4）Transparency or understandability/Autonomy |
| Paladugu et al.(169) | 2023 | United States | To discuss the implementation of Generative Adversarial Networks (GANs) in healthcare as a machine learning technique to improve medical images and AI training. | N/A | Editorial | Policymakers must consider issues related to use of GANs, such as data privacy and mismanagement or misuse, including the production of deepfakes and the use of images to generate new data without a patient's consent. | 1.Beneficence 2.Non-maleficence 3.Justice 4.Respect for autonomy | （1）Clinician–patient relationship and communication （2）Need for human regulation; oversight/Privacy and data protection （3）Equity, fairness/Bias, discrimination/Clear assumption of responsibility, accountability （4）Informed consent |
| Paravastu et al.(170) | 2022 | United States | To outline current threats and mitigation strategies related to integrity controls for medical imaging data, in light of increasing adoption of AI. | N/A | Scoping review | Current threats to integrity controls for medical imaging data include deep-fakes, data manipulation, and identity counterfeiting. Mitigation strategies can be drawn from exemplary cases. | 2.Non-maleficence | （2）Privacy and data protection/Misinformation |
| Parker et al.(171) | 2021 | Canada | To guide medical imaging stakeholders on best practices for data management, access to data, de-identification, and accountability, and to inform members of the Canadian Association of Radiologists on practical aspects of de-identification of medical images, limitations of current approaches, and future directions. | N/A | White paper | Application of AI in radiology requires accessing large datasets that contain patient health information. There are practical applications of protecting patient data in the current Canadian clinical landscape. | 1.Beneficence 2.Non-maleficence | （1）Accuracy and efficiency （2）Privacy and data protection/Insufficient data volume, generalizability concerns |
| Pashkov et al.(172) | 2020 | Ukraine | To describe specifics of AI in healthcare and to propose strategies for eliminating complexities of its implementation. | N/A | Literature review | The main challenges of AI implementation in healthcare include safety and efficiency, privacy, and various ethical and liability concerns. | 1.Beneficence 3.Justice 4.Respect for autonomy | （1）Promotion of well-being （2）Clear assumption of responsibility, accountability （3）Informed consent |
| Pedersen et al.(173) | 2020 | Australia | To describe core concepts of AI, particularly deep learning, with respect to how AI might be used to support clinical decisions. | N/A | Editorial | AI has potential to reduce uncertainty surrounding diagnosis and treatment of neurological disease. Significant effort is needed toward high-quality clinical studies. | 1.Beneficence | （1）Accuracy and efficiency |
| Pergolizzi et al.(174) | 2023 | United States | To review the variety of new technologies and advancements that digital health brings to healthcare. | N/A | Narrative Review | Digital health is changing the world of healthcare more rapidly than it can be regulated and healthcare professionals can adjust. While it allows patients to become more informed and monitor their own health, it cannot serve as a replacement for traditional care. Digital health has the potential for misuse and privacy issues. | 2.Non-maleficence 4.Respect for autonomy | （3）Privacy and data protection （4）Transparency or understandability |
| Pierre et al.(175) | 2023 | United States | To outline the role of AI in process improvement and workflow enhancement in radiology. | N/A | Editorial | The role of AI in enhancement of processes and workflows in radiology includes applications at the time of order entry and scan acquisition, applications supporting image interpretation, and applications supporting post-interpretation tasks such as communication of results. | 1.Beneficence | （1）Quality and safety of care/Accuracy and efficiency |
| Prabhakaran et al.(176) | 2019 | India | To describe technological innovations that have potential to strengthen primary care and reduce health inequities, along with the challenges that accompany these innovations. | N/A | Editorial | AI and other technological innovations are being evaluated in Indian medicine with mixed success. If applied optimally, many of these innovations could help address healthcare inequities. Salient challenges include scale, reach, and sustainability. | 1.Beneficence 3.Justice | （1）Quality and safety of care （3）Accessibility of services |
| Pruinelli and Michalowski(177) | 2021 | United States | To describe the future of augmented nursing. | N/A | Editorial | As use of AI increases, it will be crucial for nurses to be proactive about interacting with these new applications and to be involved in their design and implementation. | 1.Beneficence 2.Non-maleficence | （1）Patient-centered care/Accuracy and efficiency （2）Privacy and data protection |
| Rabbani et al.(178) | 2023 | United States | To assess whether a natural language processing algorithm can accurately identify confidential content within clinical progress notes for adolescents. | 240 clinical progress notes | Quantitative descriptive analysis | The algorithm tested in this study demonstrated efficiency gains compared to manual note review, for identifying confidential content in adolescent clinical progress notes. | 2.Non-maleficence | （2）Privacy and data protection |
| Rajpurkar et al.(179) | 2022 | United States | To describe how AI is expected to broadly transform medicine and improve experiences of both patients and clinicians. | N/A | Literature review | Medical AI has advanced considerably through RCTs and other prospective studies as well as medical image analysis. However, as a field, it remains at an early phase of validation and implementation. | 1.Beneficence 2.Non-maleficence 3.Justice | （1）Clinical validity of algorithms （2）Privacy and data protection （3）Clear assumption of responsibility, accountability/Equity, fairness/Bias, discrimination |
| Ramsdale et al.(180) | 2021 | United States | To outline steps in implementation of ML, discuss how it is different from traditional statistics approaches, and compare effectiveness of model outputs in geriatric oncology. | N/A | Editorial | ML is increasingly being used for analysis of healthcare data. Clinicians need to become familiar with basic ML concepts. Constraints on the application of ML in geriatric oncology include small available datasets and limited workforce with expertise in healthcare ML. | 2.Non-maleficence 4.Respect for autonomy | （2）Insufficient data volume, generalizability concerns/Low data quality, accuracy and reliability concerns （4）Transparency or understandability |
| Randazzo et al.(181) | 2023 | Italy | To explore the benefits of the Metaverse in the field of urology. | N/A | Literature Review | The Metaverse has an immense amount of potential in urology, including pain management, education, patient support, remote access to healthcare, surgical practices, and research. Responsible practices and policies must be put in place to regulate use of the Metaverse. | 2.Non-maleficence 3.Justice | （2）Privacy and data protection （3）Affordability and availability/Bias, discrimination |
| Ribeiro et al.(182) | 2023 | Brazil | To test the POTTER calculator as a method of calculating mortality and morbidity in emergency surguries. | 194 patients in trauma and non-trauma emergency surgery | Quasi-experimental study  (After a systematic review of medical records, POTTER predictions were calculated for 30-day and 30-day morbidity rates of each patient using existing algorithms and the phone app; Outcomes: POTTER Accuracy in predicting both 30-day mortality and 30-day morbidity) | The POTTER calculator proved to have excellent performance in predicting morbidity and mortality in this study. | 1.Beneficence | （1）Accuracy and efficiency |
| Roberts et al.(183) | 2024 | England | To test the capabilities of LLMs to create clinical letters for complicated profiles, increasing clinician efficiency and improving patient comprehension. | 42 hypothetical clinical letters created by ChatGPT-4, ChatGPT-3.5, or Google Bard | Cross-sectional quantitative study | There were varying levels of accuracy and readability among all LLMs evaluated. Reading level of letters was typically higher than desired. ChatGPT-4 proved to be the most precise in interpreting the information from the BAAPS and ASPS profiles. There is remaining concern about errors and lack of clinical knowledge of LLMS. | 1.Beneficence 4.Respect for autonomy | （1）Accuracy and efficiency/Clinician–patient relationship and communication （4）Shared decision-making |
| Rodler et al.(184) | 2023 | Germany | To explore patient trust in the use of AI for decision making and future applications of AI in urology. | 466 patients receiving diagnostic or therapeutic interventions for prostate cancer | Cross-sectional quantitative study | Trust in AI and use of technology were strongly positively correlated. Patients showed significantly higher trust in diagnoses made by physicians over AI, and AI regulated by physicians over non-regulated AI. Patients' overall preferred treatment setting was physicians assisted by AI. The study shows the importance of integrating AI in collaboration with physicians and identifying factors for strengthening trust in AI. | 2.Non-maleficence 4.Respect for autonomy | （2）Trust in implementing AI for healthcare （4）Transparency or understandability |
| Rose et al.(185) | 2023 | United States | To explore barriers to AI effectiveness stemming from missingness in health data and discuss potential solutions. | 164 attendees of a digital conference (represented by 13 expert speakers, as the study participants) | Qualitative thematic analysis | Problems identified to be stemming from missingness in data include lack of representation, inaccuracy of predictive models, needed human input, and trust and privacy issues. Suggested strategies included ensuring inclusive data collection, improving transparency and communication to foster trust in AI, developing clear regulations, involving communities and medical professionals in research and AI development, and testing AI models for fairness and generalizability. | 2.Non-maleficence 3.Justice 4.Respect for autonomy | （2）Privacy and data protection/Insufficient data volume for use of machine learning/concerns about generalizability/Low data quality, accuracy and reliability concerns （3）Bias, discrimination （4）Transparency or understandability |
| Rowell and Sebro(186) | 2022 | United States | To describe processes around data, consent, and the potential financial stakeholders involved in development of medical AI. | N/A | Editorial | The question of data ownership is particularly important in the use of AI in healthcare. Development of AI involves multiple stakeholders, and it is difficult to dissect individual contributions of each stakeholder. There is urgent need for discussion about data ownership in medical AI and how data use will be reimbursed. | 4.Respect for autonomy | （4）Informed consent |
| Russell et al.(187) | 2023 | United States | To identify AI-related clinical competencies for healthcare providers. | 15 healthcare AI experts | Qualitative thematic analysis | There is a need for healthcare providers to become competent in the following domains: (1) basic knowledge about AI; (2) social and ethical implications of AI; (3) AI enhancement of clinical encounters; (4) evidence-based evaluation of AI; (5) workflow analysis of AI; and (6)practice-based learning and improvement around AI. | 3.Justice | （3）Clear assumption of responsibility, accountability/Equity, fairness/Bias, discrimination |
| Saba et al.(188) | 2019 | Italy | To describe the influence of deep learning on healthcare, particularly radiology. | 150 articles | Literature review | If properly utilized, deep learning has potential to improve outcomes and reduce costs in radiology. | 2.Non-maleficence 3.Justice | （2）Need for human regulation; oversight/Privacy and data protection （3）Clear assumption of responsibility, accountability |
| Sangers et al.(189) | 2023 | Netherlands | To examine the opinions of dermatologists and general practitioners (GPs) on the use of AI in detecting skin cancer. | 16 dermatologist, 17 GPs | Qualitative focus group study | Dermatologists and GPs identified many potential benefits to the use of AI in skin cancer care, including its educational capabilities and improved diagnostic accuracy, which would lead to lower costs in care, earlier detection, and improved confidence in skin assessments. Some possible barriers are privacy concerns, questioning of AI accuracy, potential health inequalities, and fear of being replaced by AI. | 2.Non-maleficence 3.Justice | （2）Privacy and data protection （3）Equity, fairness |
| Sezgin et al.(190) | 2023 | United States | To present the opportunity for collaboration between humans and AI, and to use AI as a tool rather than a replacement for existing systems. | N/A | Editorial | A human-in-the-loop approach can be used to foster collaboration between physicians and AI, allowing the expertise of doctors to be enhanced by the quality control and efficiency of AI, limiting mistakes and maintaining timeliness in the healthcare process. Further, to allow the transition to using AI systems in healthcare, organizations must implement regulatory frameworks and ensure rigorous evaluation to make sure AI systems are safe to use. | 1.Beneficence | （1）Promotion of well-being/Clinician–patient relationship and communication |
| Sheikh et al.(191) | 2021 | United Kingdom | To describe the UK's utilization of health information technology (HIT) during the COVID-19 pandemic and to outline priorities and opportunities to strengthen HIT in the UK. | N/A | Editorial | The COVID-19 pandemic accelerated digitization of the UK healthcare system. However, persistent barriers were apparent during the pandemic as well, including poor interoperability, privacy concerns, and difficulties related to data flow. These barriers provide targets to address in future policy. | 2.Non-maleficence | （2）Privacy and data protection |
| Shuaib et al.(192) | 2020 | Kuwait | To describe successes, opportunities, and challenges related to integration of AI into healthcare. | N/A | Editorial | Use of AI is growing rapidly in healthcare. While there is potential for AI to replace human clinicians in some roles, it is more likely for AI to augment the capabilities of human clinicians. There is a need to better understand challenges associated with AI and potential technological singularity in order to better prepare for upcoming change in healthcare. | 3.Justice | （3）Clear assumption of responsibility, accountability |
| Siddiqi et al.(193) | 2023 | Singapore | To test Bablibot (AI chatbot) in answering questions about immunization from caregivers in Pakistan and test its use in low-resource settings. | 677 users of Bablibot | Mixed methods descriptive study | Balibot returned a high satisfaction rate of 90% after conversations with its users. Bablibot was high performing technically, being fully functional over 95% of the time after development of the beta version. Results find that a local language AI chatbot is a possible method for delivering immunization information to caregivers in limited resource settings. | 1.Beneficence 3.Justice 4.Respect for autonomy | （1）Promotion of well-being/Personalized medicine （3）Accessibility of services （4）Patients' health-related knowledge |
| Sidebottom et al.(194) | 2021 | United Kingdom | To describe the background of healthcare AI and issues around its use. | N/A | Editorial | Large-scale, well-curated clinical datasets will be essential to realizing the benefits of healthcare AI. | 2.Non-maleficence | （2）Low data quality, accuracy and reliability concerns |
| Sim et al.(195) | 2023 | Singapore | To emphasize the efficiency improvements and cost savings that can be made through the use of AI in readiology services, using Singapore as an example. | N/A | Literature review | Radiology services have seen a wide increase in demand. AI has the potential to make the imaging process much more efficient and help with early detection of illness to lower the burden of chronic disease. | 1.Beneficence | （1）Promotion of well-being/Patient-centered care/Accuracy and efficiency |
| Spear et al.(196) | 2023 | United States | To explain how AI can improve quality of care for patients and physician experience, and lower costs. | N/A | Editorial | AI can relieve physician burnout by taking care of administrative tasks and can also help with improving productivity by helping with diagnoses. While some people may be concerned about AI costs or AI replacing jobs, it will actually have opposite effects in the long-term, by increasing efficiency and allowing for increased demand. | 1.Beneficence | （1）Promotion of well-being/Dignity, empathy, and humanism in healthcare/Accuracy and efficiency |
| Sqalli et al.(197) | 2023 | Qatar | To summarize considerations regarding humanization of AI in medical training. | N/A | Editorial | Past research on electrocardiogram interpretation and wearable eHealth devices can inform a framework for responsible healthcare AI design. | 2.Non-maleficence 3.Justice 4.Respect for autonomy | （2）Risk; error management （3）Clear assumption of responsibility, accountability/Equity, fairness/Bias, discrimination （4）Transparency or understandability |
| Steil et al.(198) | 2019 | Germany | To discuss various viewpoints and challenges raised by novel team-machine interactions in healthcare. | N/A | Editorial | Inter- and multi-disciplinary collaboration are needed to appropriately analyze the significant change that will likely result from changing relationships between humans and machines in healthcare. | 2.Non-maleficence 3.Justice | （2） Privacy and data protection/Technology addiction, algorithmic overreliance （3）Clear assumption of responsibility, accountability |
| Stogiannos et al.(199) | 2023 | Ireland | To explore AI policies in the UK, specifically about medical imaging and radiotherapy. | N/A | Scoping review | Key components of AI governance were identified, including evaluation procedures, continuous monitoring of safety and efficiency, and compliance with regulations and appropriate accreditation bodies. Appropriate education must occur to ensure proper use and comfortability with AI tools. | 1.Beneficence 3.Justice 4.Respect for autonomy | （1）Promotion of well-being （3）Clear assumption of responsibility, accountability （4）Informed consent/Transparency or understandability |
| Sujan et al.(200) | 2022 | United Kingdom | To analyze stakeholder perceptions of healthcare AI, including potential advantages or disadvantages and needed safety or regulatory practices. | 26 participants (patients, hospital staff, technology developers, and technology regulators) | Qualitative thematic analysis | With respect to healthcare AI, needed steps include: (1) a socio-technical, systems-level approach to design; (2) continued learning from existing highly automated systems; (3) continued attention to the critical role of the patient-clinician relationship; and (4) cultural change around regulating the safety of healthcare AI and digital health in general. | 1.Beneficence 2.Non-maleficence 4.Respect for autonomy | （1）Accuracy and efficiency/Clinician–patient relationship and communication （2）Low data quality, accuracy and reliability concerns （4）Autonomy |
| Suppadungsuk et al.(201) | 2023 | United States | To address applications of AI in critical care nephrology. | N/A | Editorial | The inclusion of chatbots in critical care nephrology has many potential uses, including improved efficiency, quicker information access, improved patient monitoring, improved decision support, and personalized patient education. Some challenges that remain include integrating chatbots with already existing systems and securing the privacy of patients. | 1.Beneficence 2.Non-maleficence 3.Justice 4.Respect for autonomy | （1）Personalized medicine/Promotion of well-being/Clinician–patient relationship and communication/Quality and safety of care/ Accuracy and efficiency  （2）Privacy and data protection （3）Accessibility of services/Equity, fairness （4）Patients' health-related knowledge/Informed consent/Shared decision-making |
| Tan et al.(202) | 2024 | China | To improve adherence to exercise regimens and to boost mental health outcomes from exercise. | 40 adult participants  (comparison: 6-month exercise regimen prescribed by AI vs. standard exercise regimens; outcomes: effectiveness of regimens for health management at 6 months, 12 months, and 18 months.) | Randomized controlled trial protocol | AI is expected to provide personalized approaches to exercise plans, improving adherence to exercise plans and boosting health outcomes. | 1.Beneficence | （1）Personalized medicine/Quality and safety of care |
| Ting and Al-Aswad(203) | 2021 | Singapore | To outline the "six rights" that guide the translation of AI technologies to ophthalmology practice. | N/A | Editorial | To guide translation of AI technologies into ophthalmology practice, it is important to identify the right intended use environment, the right training and testing dataset, the right techniques, the right reporting guidelines, the right enabler, and the patient's right along with ethics of AI. | 1.Beneficence 2.Non-maleficence 3.Justice 4.Respect for autonomy | （1）Clinical validity of algorithms （2）Privacy and data protection/Need for human regulation; oversight （3）Clear assumption of responsibility, accountability （4）Transparency or understandability/Informed consent |
| Trenfield et al.(204) | 2022 | United Kingdom | To describe the benefits and challenges of virtual health interventions. | N/A | Editorial | Use of virtual technologies in healthcare is increasing and can bring benefits such as more personalized medicine, improved efficacy for treatments, and better treatment delivery to remote areas. Challenges include data security and accessibility. | 1.Beneficence 2.Non-maleficence 3.Justice 4.Respect for autonomy | （1）Accuracy and efficiency/Clinical validity of algorithms/Personalized medicine （2）Privacy and data protection/Need for human regulation; oversight （4）Transparency or understandability/Informed consent （3）Affordability and availability |
| Tustumi et al.(205) | 2023 | Brazil | To describe the potential benefits and challenges of ChatGPT in healthcare. | N/A | Editorial | ChatGPT has potential to improve patient outcomes and increase efficiency of healthcare. Challenges include risk of information bias and inability to understand context. | 1.Beneficence 3.Justice | （1）Accuracy and efficiency/Quality and safety of care （3）Bias, discrimination/Equity, fairness |
| Van Der Stigchel et al.(206) | 2023 | Netherlands | To test if human collaboration with AI is sufficient to manage biases stemming from AI. | 34 adult participants with normal vision and higher education | Quasi-experimental study  (Participants were asked to perform a triage on patients with the help of either a biased or an unbiased agent; Outcomes: triage effectiveness, trust in AI agent's advice, and participants' assessment of type of agent.) | Results suggested a lack of human capability to identify bias in AI agents. This emphasizes the crucial component of human control in decision making when using AI and the importance of acknowledging the risks of using AI. | 3.Justice 4.Respect for autonomy | （3）Bias, discrimination （4）Shared decision-making |
| Van Der Waa et al.(207) | 2021 | Netherlands | To evaluate team designs that address meaningful human control (MHC) in human-agent teams. | 7 healthcare professionals | Mixed qualitative study | Team design should support the human in holding responsibility for the agent's behavior and the team's decisions. | 1.Beneficence 3.Justice | （1）Patient-centered care/Clinical validity of algorithms （3）Algorithms designed in light of the multidimensionality of health |
| Vearrier et al.(208) | 2022 | United States | To describe legal and ethical issues of AI in emergency medicine. | N/A | Editorial | AI has significant potential benefits for clinicians and patients, which are transforming the therapeutic relationship into a triadic clinician-patient-machine relationship. Attention is needed toward legal standards, patient safeguards, and clinician education. | 1.Beneficence 2.Non-maleficence 3.Justice 4.Respect for autonomy | （1）Accuracy and efficiency/Clinical validity of algorithms （2）Privacy and data protection/Misuse （3）Clear assumption of responsibility, accountability/Bias, discrimination （4）Informed consent/Transparency or understandability |
| Vo et al.(209) | 2023 | Australia | To review opinions on AI from clinicians, patients and the general public. | N/A | Literature review | The findings of this review suggest an overall positive attitude towards AI in healthcare. However, there are still barriers. Clinicians must receive training on AI and modify their positions to include AI. Emphasis must be placed on fostering trust between patients and AI. | 1.Beneficence 2.Non-maleficence 3.Justice 4.Respect for autonomy | （1）Promotion of well-being/Clinician–patient relationship and communication （2）Risk; error management/Privacy and data protection （3）Accessibility of services/Clear assumption of responsibility, accountability/Bias, discrimination/Equity, fairness （4）Transparency or understandability/Patients' health-related knowledge; |
| Volovici et al.(210) | 2022 | The Netherlands | To outline steps to avoid overuse and misuse of machine learning in clinical research. | N/A | Editorial | Integration of ML and big data is likely to improve clinical medicine. There is a need for ML researchers to recognize the limits of algorithms and models to prevent overuse and misuse. | 2.Non-maleficence | （2）Misuse |
| Walker et al.(211) | 2023 | United States | To argue that biases in AI stem from power imbalances in the innovation and use of healthcare technologies. | N/A | Editorial | Biases in AI algorithms stem from power imbalances and biases already in place in the healthcare system. To solve this, we must have community accountability to represent the priorities and demands of all people. | 3.Justice 4.Respect for autonomy | （3）Bias, discrimination （4）Transparency or understandability |
| Wang et al.(212) | 2022 | United States | To describe metaverse use cases and identify action items for building the "medical technology and AI" (MeTAI) metaverse. | N/A | Editorial | Collective effort is needed to pioneer a metaverse. This effort could include virtual comparative scanning and raw data sharing. | 1.Beneficence 2.Non-maleficence | （1）Personalized medicine （2）Trust in implementing AI for healthcare / Privacy and data protection |
| Wang et al.(213) | 2021 | United States | To analyze the extent to which AI systems in primary care examine inherent biases concerning vulnerable populations and to assess how these systems mitigate the impact of such biases. | 405 articles | Scoping review | AI has strong potential to improve the patient-clinician relationship. Primary care is an especially promising area for AI integration. However, algorithms are susceptible to disparities in performance across subgroups. | 1.Beneficence | （1）Clinician–patient relationship and communication |
| Wang et al.(214) | 2023 | China | To summarize applications of AI in clinical decision support systems (CDSS), challenges of using AI in oncology decisions, and suggestions for applications of AI in oncology decisions. | N/A | Editorial | Concordance between CDSS-based decisions and decisions made by multidisciplinary teams will likely increase as AI technology improves over time. | 1.Beneficence | （1）Accuracy and efficiency/Clinician–patient relationship and communication |
| Watson et al.(215) | 2020 | United States | To discuss the relevance of nursing in a postmodern, technologically advanced healthcare system. | N/A | Editorial | The nursing profession must befriend AI and examine how it can augment nursing practice. Additional data that AI provides can improve patient care and enhance the nurse-patient relationship. | 1.Beneficence | （1）Accuracy and efficiency |
| Wehbe et al.(216) | 2020 | United States | To summarize current evidence on prognostic variables and comment on the current state of risk prediction for patients with heart failure. | N/A | Editorial | It is generally difficult to predict outcomes in patients with heart failure. Methods of risk prediction utilizing ML have potential but are not yet living up to expectations. | 3.Justice 4.Respect for autonomy | （3）Bias, discrimination （4）Transparency or understandability |
| Werutsky et al.(217) | 2021 | Brazil | To summarize how emerging technologies, personalized medicine, and clinical research can improve cancer control in Latin America and the Caribbean. | N/A | Editorial | Control of cancer incidence in Latin America and the Caribbean will require new technologies that are not currently widely implemented because of inadequate funding, insufficient capacity of humans trained to manage new technologies, and larger structural deficiencies. | 1.Beneficence 3.Justice | （1）Clinical validity of algorithms （3）Equity, fairness |
| Whiteson and Frishman(218) | 2023 | United States | To discuss the implementation of AI and ML technologies in cardiovascular disease procedures, including detection and prevention. | N/A | Literature review | By helping improve efficiency and accuracy, AI and ML can dramatically improve the detection and prevention of cardiovascular diseases and increase the amount of people providers can assist. AI can also make treatment more equitable by assisting lower-resource areas and can implement genomic analysis to personalize diagnosis and increase prevention. Some challenges to AI in healthcare include trust and acceptance, biases in algorithms, training of physicians to use AI, and development of regulatory frameworks. | 1.Beneficence 3.Justice 4.Respect for autonomy | （1）Promotion of well-being （3）Bias, discrimination/Equity, fairness/Clear assumption of responsibility, accountability （4）Transparency or understandability |
| Wong et al.(219) | 2023 | Malaysia | To examine the use of ChatGPT in medical spaces, including education for medical purposes. | N/A | Editorial | ChatGPT has many significant applications to healthcare, including decision assistance, data collection, disease prevention, providing information to patients and physicians, research, and participant recruitment. ChatGPT accuracy is still a major drawback to its use, and it also raises ethical issues, including privacy, accountability of AI in the event of errors, appropriate regulatory frameworks, and academic dishonesty. | 2.Non-maleficence 3.Justice 4.Respect for autonomy | （2）Misinformation/Privacy and data protection （3）Clear assumption of responsibility, accountability/Bias, discrimination （4）Transparency or understandability/Informed consent |
| Xue et al.(220) | 2023 | China | To describe the potential impact of ChatGPT in translational and clinical medicine. | N/A | Editorial | ChatGPT has potential to transform clinical and translational medicine, but appropriate strategies are needed to mitigate risks and harms. | 1.Beneficence 2.Non-maleficence 3.Justice | （1）Clinical validity of algorithms/Accuracy and efficiency/Personalized medicine/Promotion of well-being （2）Privacy and data protection （3）Bias, discrimination |
| Yang(221) | 2023 | Malaysia | To discuss the benefits and drawbacks of ChatGPT in digital health. | N/A | Editorial | Overall, ChatGPT displays immense potential to distribute information by informing patients, giving personalized health advice, and assisting decision making. ChatGPT still falls short on many occasions due to data privacy concerns, errors in diagnosis, and failure to understand human sentiment. | 1.Beneficence | （1）Promotion of well-being/Personalized medicine/Quality and safety of care/Emotional support |
| Young et al.(222) | 2020 | United States | To summarize current research on automated classification and monitoring of skin lesions, barriers to implementation, and potential metrics for evaluating AI model performance. | N/A | Editorial | AI is expected to be helpful for triaging diseases into broad categories. Dermatology clinician expertise will still be needed for more detailed diagnosis and management of conditions or unique cases that require contextual knowledge. | 2.Non-maleficence 3.Justice 4.Respect for autonomy | （2）Low data quality, accuracy and reliability concerns （3）Equity, fairness/Bias, discrimination （4）Transparency or understandability |
| Youngmann et al.(223) | 2019 | Israel | To develop and evaluate performance of a new web-based classifier designed to screen for Parkinson's disease among search engine users. | Search engine data from 1,490,987 web users in the United States  (Data was collected from January 2016-April 2017; positive predictive value was compared between user groups based on risk level.) | Quasi-experimental study | The automatic classifier in this study was able to reliably identify search engine users at high risk for Parkinson's disease. Ethical questions about use of this type of technology remain to be addressed. | 1.Beneficence 2.Non-maleficence 3.Justice | （1）Clinical validity of algorithms （2）Risk; error management/Misuse （3）Bias, discrimination |
| Zack et al.(224) | 2024 | United States | To test if GPT-4 perpetuates racial and gender biases with the potential to harm its use in healthcare. | 19 medical education cases from NEJM Healer, as prompts | Quasi-experimental study  (Tested for bias in GPT-4 by prompting it to provide treatment recommendations based on clinical vignettes; comparison group: true US prevalence estimates; standard statistical tests were used to identify differences in treatment planning across demographic groups.) | GPT-4 does hold biases and does not appropriately represent the demographic diversity of medical conditions. | 3.Justice | （3）Bias, discrimination |
| Zhang(225) | 2023 | China | To analyze drone policing applications, including bioethical and biotechnological considerations. | N/A | Editorial | There is a need for more ethically informed and technologically advanced approaches to drone policing. | 2.Non-maleficence 3.Justice 4.Respect for autonomy | （2）Privacy and data protection/ Risk; error management （3）Clear assumption of responsibility, accountability （4）Transparency or understandability |
| Zhang et al.(226) | 2023 | China | To discuss recent advancements in AI technologies used for breast cancer treatment. | N/A | Literature review | AI continues to become more involved in breast cancer imaging. AI can help with personalized diagnosis, staging, treatment, and prognosis, increasing accuracy, and assisting physicians with decision making. | 2.Non-maleficence 3.Justice 4.Respect for autonomy | （2）Privacy and data protection （3）Clear assumption of responsibility, accountability; （4）Transparency or understandability; |
| Zhang et al.(227) | 2024 | United States | To present the FDA review processes and specific regulations for AI and ML algorithms in radiology. | N/A | Literature review | FDA-approved AI/ML tools still make errors, emphasizing the importance of human interaction with AI. As algorithms continue to improve and evolve, regulations must actively and systematically evolve with them. | 4.Respect for autonomy | （4）Transparency or understandability |
| Zhang et al.(228) | 2020 | United States | To summarize applications of chatbots to promote physical activity and healthy diets, describe a chatbot behavior change model, and discuss ethical considerations around this use of chatbots. | 101 articles | Literature review | The framework developed by this review can help to conceptualize use of chatbots for physical activity and health diet promotion. There is a need for further interdisciplinary effort to guide use of chatbots for this purpose in ways that follow ethical principles. | 2.Non-maleficence 3.Justice 4.Respect for autonomy | （2）Privacy and data protection （3）Equity, fairness （4）Transparency or understandability/Informed consent |
| Zhao et al.(229) | 2021 | China | To identify potential ethical issues discovered during the COVID-19 pandemic with using AI for monitoring and preventing infectious disease outbreaks. | N/A | Literature review | The general public felt uninformed on the risks and purposes of contact tracing and desired more transparency about the technology used. In the future, a systematic framework must be put in place to regulate the use of AI in tracking disease outbreaks and to ensure data protection. | 2.Non-maleficence 4.Respect for autonomy | （2）Insufficient data volume, generalizability concerns/Low data quality, accuracy and reliability concerns/Trust in implementing AI for healthcare/Privacy and data protection （4）Informed consent |
| Zhu et al.(230) | 2022 | China | To examine current literature on ethics of smart home technologies for elderly care, including AI. | 15 articles; 1047 participants total | Scoping review | There are significant ethical issues related to the application of smart home technologies for elderly care. It is necessary to further investigate these ethical issues, weighing the advantages and disadvantages of these technologies. | 1.Beneficence 2.Non-maleficence 3.Justice 4.Respect for autonomy | （1）Dignity, empathy, and humanism in healthcare （2）Privacy and data protection （3）Equity, fairness （4）Autonomy |
| Zidaru et al.(231) | 2021 | England | To explore public engagement in design, development, testing, implementation, and evaluation of AI technologies used for mental health. | 144 articles | Scoping review | Public engagement has begun to take place in the forms of inclusive design, patient-focused networks of research, user-focused and experience-led design and research methods, and care decision making that involves communities or social networks. | 2.Non-maleficence 3.Justice | （2）Trust in implementing AI for healthcare / Privacy and data protection （3）Bias, discrimination |

**Supplementary Table 5.** Article Types for All Included Articles

| Article Type |  |  | Specific Methodology |  |  |
| --- | --- | --- | --- | --- | --- |
| Editorial | 96 | 42% | Editorial | 96 | 42% |
| Review | 73 | 32% | Literature review | 55 | 24% |
|  |  |  | Scoping review | 7 | 3% |
|  |  |  | Systematic review | 3 | 1% |
|  |  |  | Narrative review | 8 | 4% |
| Empirical | 46 | 20% | Empirical | 46 | 20% |
| Other | 12 | 5% | Conceptual analysis | 4 | 2% |
|  |  |  | Position statement | 2 | 1% |
|  |  |  | Workshop/conference report | 2 | 1% |
|  |  |  | White paper | 1 | 0% |
|  |  |  | Case Study | 3 | 1% |

**Supplementary Table 6.** Example Representative Phrases from Abstract Screening

| **Principle** | **Example Representative Phrases Identified during Abstract Screening** |
| --- | --- |
| Beneficence | “improved patient outcomes,” “elevate healthcare quality,” “augmenting patient care,” “improved diagnostic precision,” “early detection,” “risk assessment,” “identify patients who are at risk,” “prevent patient harm,” “benefitting patients” |
| Non-Maleficence | “data security,” “privacy concerns,” “safety,” “delivering incorrect medical diagnoses,” “harmful impact on medical care” |
| Respect for Autonomy | “black box problem,” “readability,” “empowerment,” “engagement in self-management,” “ensuring transparency” |
| Justice | “diagnosis in low-resource settings,” expand accessibility,” “data accessibility,” “promise underserved communities greater access to healthcare,” “addressing the challenges posed by rising costs and limited resources,” “structural inequalities,” “perpetuating biases,” “promoting equity” |

## Supplementary Figures

**Supplementary Figure 1.** Highest-Frequency Words in Excerpts Coded for Beneficence


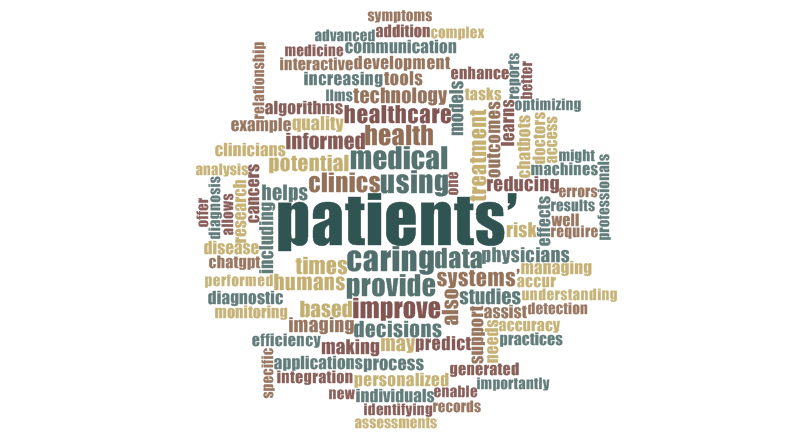


**Supplementary Figure 2.** Highest-Frequency Words in Excerpts Coded for Non-Maleficence


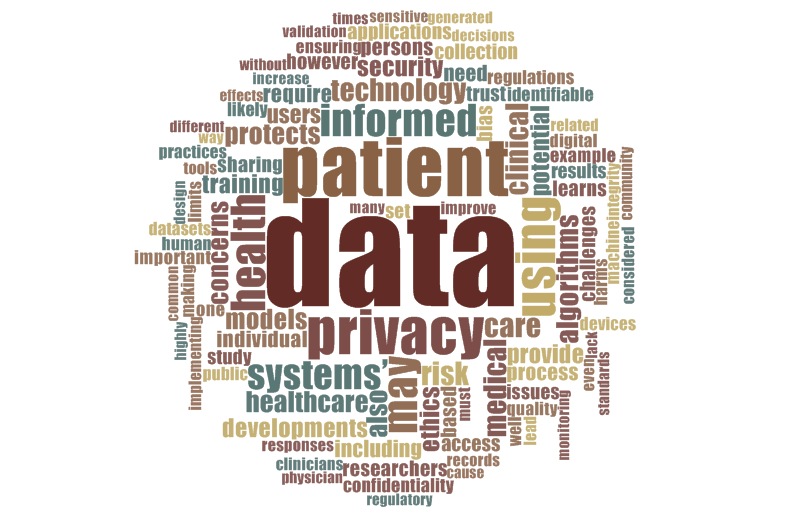


**Supplementary Figure 3.** Highest-Frequency Words in Excerpts Coded for Autonomy


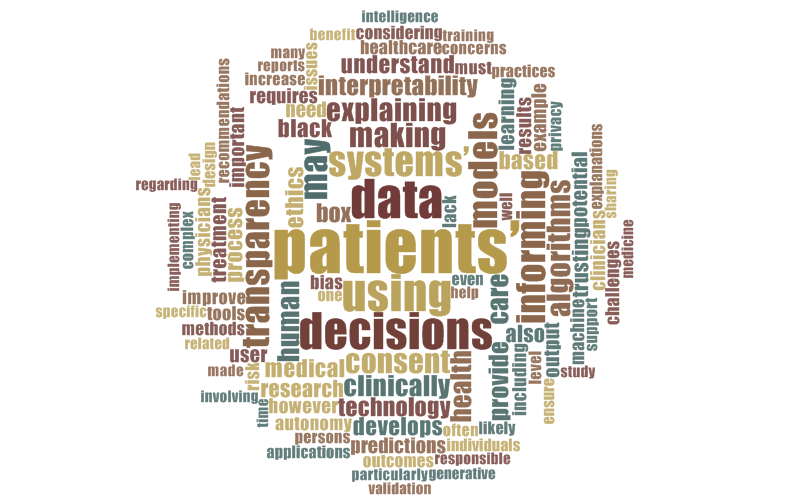


**Supplementary Figure 4.** Highest-Frequency Words in Excerpts Coded for Justice


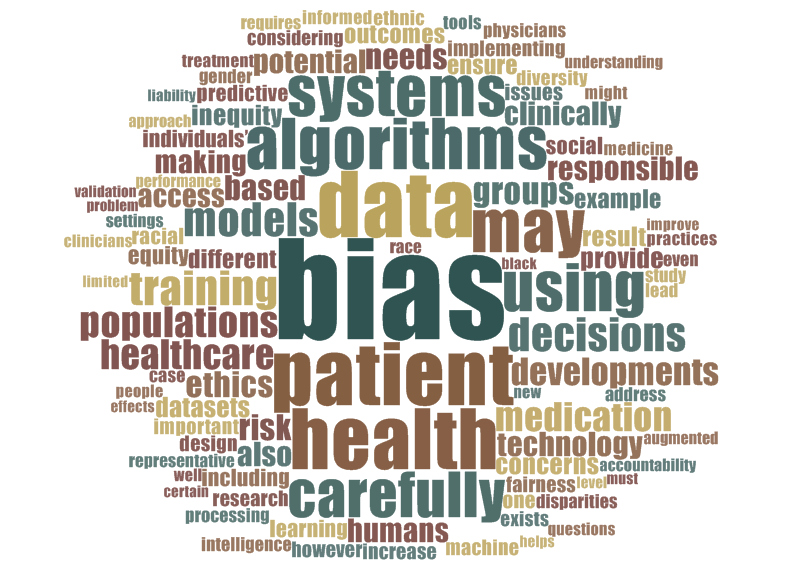


# References

1. Berretta S, Tausch A, Ontrup G, Gilles B, Peifer C, Kluge A. Defining human-AI teaming the human-centered way: a scoping review and network analysis. Front Artif Intell. 2023 Sep 29;6:1250725.

2. Beauchamp TL. Principles of Biomedical Ethics. Childress JF, editor. New York: Oxford University Press; 1979.

3. Beauchamp TL. A Defense of Universal Principles in Biomedical Ethics. In: Valdés E, Lecaros JA, editors. Biolaw and Policy in the Twenty-First Century: Building Answers for New Questions [Internet]. Cham: Springer International Publishing; 2019 [cited 2024 Aug 3]. p. 3–17. Available from: https://doi.org/10.1007/978-3-030-05903-3_1

4. World Health Organization. Digital health EURO [Internet]. [cited 2025 Jan 5]. Available from: https://www.who.int/europe/health-topics/digital-health

5. Abraham J, King CR, Meng A. Ascertaining Design Requirements for Postoperative Care Transition Interventions. Appl Clin Inform. 2021 Jan;12(1):107–15.

6. Abràmoff MD, Cunningham B, Patel B, Eydelman MB, Leng T, Sakamoto T, et al. Foundational Considerations for Artificial Intelligence Using Ophthalmic Images. Ophthalmology. 2022 Feb;129(2):e14–32.

7. Adams MCB, Nelson AM, Narouze S. Daring discourse: artificial intelligence in pain medicine, opportunities and challenges. Reg Anesth Pain Med [Internet]. 2023 May 11 [cited 2025 Jan 5]; Available from: https://rapm.bmj.com/content/early/2023/05/10/rapm-2023-104526

8. Adida S, Legarreta AD, Hudson JS, McCarthy D, Andrews E, Shanahan R, et al. Machine Learning in Spine Surgery: A Narrative Review. Neurosurgery. 2024 Jan;94(1):53.

9. Afnan MAM, Liu Y, Conitzer V, Rudin C, Mishra A, Savulescu J, et al. Interpretable, not black-box, artificial intelligence should be used for embryo selection. Hum Reprod Open. 2021;2021(4):hoab040.

10. Agmon S, Gillis P, Horvitz E, Radinsky K. Gender-sensitive word embeddings for healthcare. J Am Med Inform Assoc. 2022 Jan 29;29(3):415–23.

11. Ahmad OF, Stoyanov D, Lovat LB. Human-machine collaboration: bringing artificial intelligence into colonoscopy. Frontline Gastroenterology. 2018 Oct 15;10(2):198.

12. Ahmed MN, Toor AS, O’Neil K, Friedland D. Cognitive Computing and the Future of Health Care Cognitive Computing and the Future of Healthcare: The Cognitive Power of IBM Watson Has the Potential to Transform Global Personalized Medicine. IEEE Pulse. 2017 May;8(3):4–9.

13. Ahmed Z, Mohamed K, Zeeshan S, Dong X. Artificial intelligence with multi-functional machine learning platform development for better healthcare and precision medicine. Database (Oxford). 2020 Jan 1;2020:baaa010.

14. Alfano L, Malcotti I, Ciliberti R. Psychotherapy, artificial intelligence and adolescents: ethical aspects. Journal of Preventive Medicine and Hygiene. 2024 Jan 1;64(4):E438.

15. Alhasan K, Raina R, Jamal A, Temsah MH. Combining human and AI could predict nephrologies future, but should be handled with care. Acta Paediatr. 2023 Sep;112(9):1844–8.

16. Allam A, Schulz PJ, Nakamoto K. The impact of search engine selection and sorting criteria on vaccination beliefs and attitudes: two experiments manipulating Google output. J Med Internet Res. 2014 Apr 2;16(4):e100.

17. Allouch M, Azaria A, Azoulay R. Conversational Agents: Goals, Technologies, Vision and Challenges. Sensors (Basel). 2021 Dec 17;21(24):8448.

18. Almazyad M, Aljofan F, Abouammoh NA, Muaygil R, Malki KH, Aljamaan F, et al. Enhancing Expert Panel Discussions in Pediatric Palliative Care: Innovative Scenario Development and Summarization With ChatGPT-4. Cureus. 2023 Apr;15(4):e38249.

19. Alrassi J, Katsufrakis PJ, Chandran L. Technology Can Augment, but Not Replace, Critical Human Skills Needed for Patient Care. Acad Med. 2021 Jan 1;96(1):37–43.

20. Altamimi I, Altamimi A, Alhumimidi AS, Altamimi A, Temsah MH. Artificial Intelligence (AI) Chatbots in Medicine: A Supplement, Not a Substitute. Cureus. 2023 Jun 25;15(6):e40922.

21. AlZaabi A, AlMaskari S, AalAbdulsalam A. Are physicians and medical students ready for artificial intelligence applications in healthcare? Digit Health. 2023;9:20552076231152167.

22. Anderson M, Anderson SL. How Should AI Be Developed, Validated, and Implemented in Patient Care? AMA J Ethics. 2019 Feb 1;21(2):E125-130.

23. Angelopoulou A, Kapetanios E, Smith DH, Steuber V, Woll B, Zeller F. Editorial: Explanation in human-AI systems. Front Artif Intell [Internet]. 2022 Oct 26 [cited 2025 Jan 5];5. Available from: https://www.frontiersin.org/journals/artificial-intelligence/articles/10.3389/frai.2022.1048568/full

24. Arnold MH. Teasing out Artificial Intelligence in Medicine: An Ethical Critique of Artificial Intelligence and Machine Learning in Medicine. Journal of Bioethical Inquiry. 2021 Jan 7;18(1):121.

25. Arshad HB, Butt SA, Khan SU, Javed Z, Nasir K. ChatGPT and Artificial Intelligence in Hospital Level Research: Potential, Precautions, and Prospects. Methodist DeBakey Cardiovascular Journal. 2023 Nov 16;19(5):77.

26. Athreya AP, Iyer R, Wang L, Weinshilboum RM, Bobo WV. Integration of Machine Learning and Pharmacogenomic Biomarkers for Predicting Response to Antidepressant Treatment: Can Computational Intelligence be used to Augment Clinical Assessments? Pharmacogenomics. 2019 Sep 1;20(14):983–8.

27. Aung YYM, Wong DCS, Ting DSW. The promise of artificial intelligence: a review of the opportunities and challenges of artificial intelligence in healthcare. Br Med Bull. 2021 Sep 10;139(1):4–15.

28. Ayorinde JOO, Citterio F, Landrò M, Peruzzo E, Islam T, Tilley S, et al. Artificial Intelligence You Can Trust: What Matters Beyond Performance When Applying Artificial Intelligence to Renal Histopathology? J Am Soc Nephrol. 2022 Dec;33(12):2133–40.

29. Badri A, Boudreau-Trudel B, Souissi AS. Occupational health and safety in the industry 4.0 era: A cause for major concern? Safety Science. 2018 Nov 1;109:403–11.

30. Bakken S. AI in health: keeping the human in the loop. Journal of the American Medical Informatics Association. 2023 Jul 1;30(7):1225–6.

31. Balcombe L, De Leo D. Digital Mental Health Challenges and the Horizon Ahead for Solutions. JMIR Ment Health. 2021 Mar 29;8(3):e26811.

32. Bandyopadhyay A, Goldstein C. Clinical applications of artificial intelligence in sleep medicine: a sleep clinician’s perspective. Sleep Breath. 2023 Mar;27(1):39–55.

33. Banerjee I, Bhattacharjee K, Burns JL, Trivedi H, Purkayastha S, Seyyed-Kalantari L, et al. “Shortcuts” Causing Bias in Radiology Artificial Intelligence: Causes, Evaluation, and Mitigation. J Am Coll Radiol. 2023 Sep;20(9):842–51.

34. Bartenschlager CC, Grieger M, Erber J, Neidel T, Borgmann S, Vehreschild JJ, et al. Covid-19 triage in the emergency department 2.0: how analytics and AI transform a human-made algorithm for the prediction of clinical pathways. Health Care Manag Sci. 2023 Sep;26(3):412–29.

35. Batlle JC, Dreyer K, Allen B, Cook T, Roth CJ, Kitts AB, et al. Data Sharing of Imaging in an Evolving Health Care World: Report of the ACR Data Sharing Workgroup, Part 1: Data Ethics of Privacy, Consent, and Anonymization. J Am Coll Radiol. 2021 Dec;18(12):1646–54.

36. Baumgartner C. The opportunities and pitfalls of ChatGPT in clinical and translational medicine. Clinical and Translational Medicine. 2023;13(3):e1206.

37. Baumgartner R, Arora P, Bath C, Burljaev D, Ciereszko K, Custers B, et al. Fair and equitable AI in biomedical research and healthcare: Social science perspectives. Artif Intell Med. 2023 Oct;144:102658.

38. Bays HE, Fitch A, Cuda S, Gonsahn-Bollie S, Rickey E, Hablutzel J, et al. Artificial intelligence and obesity management: An Obesity Medicine Association (OMA) Clinical Practice Statement (CPS) 2023. Obes Pillars. 2023 Jun;6:100065.

39. Bazoukis G, Hall J, Loscalzo J, Antman EM, Fuster V, Armoundas AA. The inclusion of augmented intelligence in medicine: A framework for successful implementation. Cell Rep Med. 2022 Jan 18;3(1):100485.

40. Bekbolatova M, Mayer J, Ong CW, Toma M. Transformative Potential of AI in Healthcare: Definitions, Applications, and Navigating the Ethical Landscape and Public Perspectives. Healthcare (Basel). 2024 Jan 5;12(2):125.

41. Beltrami EJ, Brown AC, Salmon PJM, Leffell DJ, Ko JM, Grant-Kels JM. Artificial intelligence in the detection of skin cancer. J Am Acad Dermatol. 2022 Dec;87(6):1336–42.

42. Benzinger L, Ursin F, Balke WT, Kacprowski T, Salloch S. Should Artificial Intelligence be used to support clinical ethical decision-making? A systematic review of reasons. BMC Medical Ethics. 2023 Jul 6;24(1):48.

43. Bergquist T, Schaffter T, Yan Y, Yu T, Prosser J, Gao J, et al. Evaluation of crowdsourced mortality prediction models as a framework for assessing artificial intelligence in medicine. J Am Med Inform Assoc. 2023 Dec 22;31(1):35–44.

44. Bernstein IA, Zhang YV, Govil D, Majid I, Chang RT, Sun Y, et al. Comparison of Ophthalmologist and Large Language Model Chatbot Responses to Online Patient Eye Care Questions. JAMA Netw Open. 2023 Aug 1;6(8):e2330320.

45. Berridge C, Turner NR, Liu L, Karras SW, Chen A, Fredriksen-Goldsen K, et al. Advance Planning for Technology Use in Dementia Care: Development, Design, and Feasibility of a Novel Self-administered Decision-Making Tool. JMIR Aging. 2022 Jul 27;5(3):e39335.

46. Beyeler M, Sanchez-Garcia M. Towards aSmart Bionic Eye: AI-powered artificial vision for the treatment of incurable blindness. J Neural Eng. 2022 Dec 7;19(6).

47. Bhardwaj A. Promise and Provisos of Artificial Intelligence and Machine Learning in Healthcare. Journal of Healthcare Leadership. 2022 Jul 20;14:113.

48. Bhattacharyya GS, Bothra SJ, Malhotra H, Govindbabu K. Solving the Malady of Financial Toxicity Using Augmented Intelligence. JCO Clin Cancer Inform. 2021 Mar;5:348–52.

49. Bhowmik A, Ghosh B, Pal M, Paul RR, Chatterjee J, Chakraborty S. Portable, handheld, and affordable blood perfusion imager for screening of subsurface cancer in resource-limited settings. Proceedings of the National Academy of Sciences. 2022 Jan 11;119(2):e2026201119.

50. Bickmore T, Gruber A. Relational agents in clinical psychiatry. Harv Rev Psychiatry. 2010;18(2):119–30.

51. Bishara A, Maze EH, Maze M. Considerations for the implementation of machine learning into acute care settings. Br Med Bull. 2022 Mar 21;141(1):15–32.

52. Blanco-González A, Cabezón A, Seco-González A, Conde-Torres D, Antelo-Riveiro P, Piñeiro Á, et al. The Role of AI in Drug Discovery: Challenges, Opportunities, and Strategies. Pharmaceuticals (Basel). 2023 Jun 18;16(6):891.

53. Bleher H, Braun M. Diffused responsibility: attributions of responsibility in the use of AI-driven clinical decision support systems. AI Ethics. 2022;2(4):747–61.

54. Borgstadt JT, Kalpas EA, Pond HM. A Qualitative Thematic Analysis of Addressing the Why: An Artificial Intelligence (AI) in Healthcare Symposium. Cureus. 2022 Mar;14(3):e23704.

55. Boulos MNK, Wilson JT, Clauson KA. Geospatial blockchain: promises, challenges, and scenarios in health and healthcare. Int J Health Geogr. 2018 Jul 5;17(1):25.

56. Briggs LG, Labban M, Alkhatib K, Nguyen DD, Cole AP, Trinh QD. Digital technologies in cancer care: a review from the clinician’s perspective. J Comp Eff Res. 2022 May;11(7):533–44.

57. Brown C, Nazeer R, Gibbs A, Page PL, Mitchell AR. Breaking Bias: The Role of Artificial Intelligence in Improving Clinical Decision-Making. Cureus. 2023 Mar 20;15(3):e36415.

58. Brown Z, Bergman D, Holt L, Miller K, Frownfelter J, Bleau H, et al. Augmenting a Transitional Care Model With Artificial Intelligence Decreased Readmissions. J Am Med Dir Assoc. 2023 Jul;24(7):958–63.

59. Bunning BJ, Hedlin H, Chen JH, Ciolino JD, Ferstad JO, Fox E, et al. The evolving role of data & safety monitoring boards for real-world clinical trials. Journal of Clinical and Translational Science. 2023 Aug 2;7(1):e179.

60. Burlina P, Joshi N, Paul W, Pacheco KD, Bressler NM. Addressing Artificial Intelligence Bias in Retinal Diagnostics. Translational Vision Science & Technology. 2021 Feb 11;10(2):13.

61. Burr C, Taddeo M, Floridi L. The Ethics of Digital Well-Being: A Thematic Review. Sci Eng Ethics. 2020 Aug 1;26(4):2313–43.

62. Capelleras M, Soto-Galindo GA, Cruellas M, Apaydin F. ChatGPT and Rhinoplasty Recovery: An Exploration of AI’s Role in Postoperative Guidance. Facial Plast Surg. 2024 Oct;40(5):628–31.

63. Chambers R, Beaney P. The potential of placing a digital assistant in patients’ homes. Br J Gen Pract. 2020 Jan;70(690):8–9.

64. Chandler C, Foltz PW, Elvevåg B. Improving the Applicability of AI for Psychiatric Applications through Human-in-the-loop Methodologies. Schizophr Bull. 2022 Sep 1;48(5):949–57.

65. Chen H. Application progress of artificial intelligence and augmented reality in orthopaedic arthroscopy surgery. Journal of Orthopaedic Surgery and Research. 2023 Oct 14;18(1):775.

66. Chen H, Cohen E, Wilson D, Alfred M. A Machine Learning Approach with Human-AI Collaboration for Automated Classification of Patient Safety Event Reports: Algorithm Development and Validation Study. JMIR Human Factors. 2024 Jan 25;11:e53378.

67. Chen S, Owolabi Y, Dulin M, Robinson P, Witt B, Samoff E. Applying a machine learning modelling framework to predict delayed linkage to care in patients newly diagnosed with HIV in Mecklenburg County, North Carolina, USA. AIDS. 2021 May 1;35(Suppl 1):S29–38.

68. Chew HSJ. The Use of Artificial Intelligence-Based Conversational Agents (Chatbots) for Weight Loss: Scoping Review and Practical Recommendations. JMIR Med Inform. 2022 Apr 13;10(4):e32578.

69. Christodoulou KC, Tsoucalas G. Artificial Intelligence-Oriented Heart Surgery: A Complex Bioethical Concept. Cureus. 2023 Jul;15(7):e41911.

70. Clement J, Maldonado AQ. Augmenting the Transplant Team With Artificial Intelligence: Toward Meaningful AI Use in Solid Organ Transplant. Front Immunol. 2021;12:694222.

71. Condado PA, Lobo FG, Carita T. Towards Richer Assisted Living Environments. SN Comput Sci. 2022;3(1):96.

72. Creed TA, Kuo PB, Oziel R, Reich D, Thomas M, O’Connor S, et al. Knowledge and Attitudes Toward an Artificial Intelligence-Based Fidelity Measurement in Community Cognitive Behavioral Therapy Supervision. Adm Policy Ment Health. 2022 May;49(3):343–56.

73. Crigger E, Khoury C. Making Policy on Augmented Intelligence in Health Care. AMA J Ethics. 2019 Feb 1;21(2):E188-191.

74. Crigger E, Reinbold K, Hanson C, Kao A, Blake K, Irons M. Trustworthy Augmented Intelligence in Health Care. J Med Syst. 2022 Jan 12;46(2):12.

75. Cuocolo R, Imbriaco M. Machine learning solutions in radiology: does the emperor have no clothes? Eur Radiol. 2021 Jun;31(6):3783–5.

76. Currie G, Rohren E. Social Asymmetry, Artificial Intelligence and the Medical Imaging Landscape. Semin Nucl Med. 2022 Jul;52(4):498–503.

77. Dankwa-Mullan I, Weeraratne D. Artificial Intelligence and Machine Learning Technologies in Cancer Care: Addressing Disparities, Bias, and Data Diversity. Cancer Discov. 2022 Jun 2;12(6):1423–7.

78. de Batlle J, Benítez ID, Moncusí-Moix A, Androutsos O, Angles Barbastro R, Antonini A, et al. GATEKEEPER’s Strategy for the Multinational Large-Scale Piloting of an eHealth Platform: Tutorial on How to Identify Relevant Settings and Use Cases. J Med Internet Res. 2023 Jun 28;25:e42187.

79. Deferio JJ, Breitinger S, Khullar D, Sheth A, Pathak J. Social determinants of health in mental health care and research: a case for greater inclusion. J Am Med Inform Assoc. 2019 Aug 1;26(8–9):895–9.

80. Di Nuovo A. Letter to the Editor: “How Can Biomedical Engineers Help Empower Individuals With Intellectual Disabilities? The Potential Benefits and Challenges of AI Technologies to Support Inclusivity and Transform Lives.” IEEE J Transl Eng Health Med. 2023;12:256–7.

81. Diaz-Asper C, Hauglid MK, Chandler C, Cohen AS, Foltz PW, Elvevåg B. A framework for language technologies in behavioral research and clinical applications: Ethical challenges, implications, and solutions. Am Psychol. 2024 Jan;79(1):79–91.

82. Dixon BE, Holmes JH. Special Section on Inclusive Digital Health: Notable Papers on Addressing Bias, Equity, and Literacy to Strengthen Health Systems. Yearbook of Medical Informatics. 2022 Dec 4;31(1):100.

83. Doyen S, Dadario NB. 12 Plagues of AI in Healthcare: A Practical Guide to Current Issues With Using Machine Learning in a Medical Context. Front Digit Health. 2022;4:765406.

84. Dzobo K, Adotey S, Thomford NE, Dzobo W. Integrating Artificial and Human Intelligence: A Partnership for Responsible Innovation in Biomedical Engineering and Medicine. OMICS. 2020 May;24(5):247–63.

85. Etienne H, Hamdi S, Le Roux M, Camuset J, Khalife-Hocquemiller T, Giol M, et al. Artificial intelligence in thoracic surgery: past, present, perspective and limits. Eur Respir Rev. 2020 Sep 30;29(157):200010.

86. Feinstein M, Katz D, Demaria S, Hofer IS. Remote Monitoring and Artificial Intelligence: Outlook for 2050. Anesth Analg. 2024 Feb 1;138(2):350–7.

87. Fisher S, Rosella LC. Priorities for successful use of artificial intelligence by public health organizations: a literature review. BMC Public Health. 2022 Nov 22;22:2146.

88. Forghani R. A Practical Guide for AI Algorithm Selection for the Radiology Department. Semin Roentgenol. 2023 Apr;58(2):208–13.

89. Fox S. Behavioral Ethics Ecologies of Human-Artificial Intelligence Systems. Behavioral Sciences. 2022 Apr;12(4):103.

90. Frehywot S, Vovides Y. An equitable and sustainable community of practice framework to address the use of artificial intelligence for global health workforce training. Hum Resour Health. 2023 Jun 13;21(1):45.

91. Fuhrman JD, Gorre N, Hu Q, Li H, El Naqa I, Giger ML. A review of explainable and interpretable AI with applications in COVID-19 imaging. Med Phys. 2022 Jan;49(1):1–14.

92. Garg S. ChatGPT is still struggling to revolutionize mental health policy. Asian J Psychiatr. 2024 Mar;93:103906.

93. Gauss T, Perkins Z, Tjardes T. Current knowledge and availability of machine learning across the spectrum of trauma science. Curr Opin Crit Care. 2023 Dec 1;29(6):713–21.

94. Geraci J, Bhargava R, Qorri B, Leonchyk P, Cook D, Cook M, et al. Machine learning hypothesis-generation for patient stratification and target discovery in rare disease: our experience with Open Science in ALS. Frontiers in Computational Neuroscience. 2024 Jan 4;17:1199736.

95. Goldstein CA, Berry RB, Kent DT, Kristo DA, Seixas AA, Redline S, et al. Artificial intelligence in sleep medicine: an American Academy of Sleep Medicine position statement. J Clin Sleep Med. 2020 Apr 15;16(4):605–7.

96. Göndöcs D, Dörfler V. AI in medical diagnosis: AI prediction & human judgment. Artificial Intelligence in Medicine. 2024 Mar 1;149:102769.

97. González-Gonzalo C, Thee EF, Klaver CCW, Lee AY, Schlingemann RO, Tufail A, et al. Trustworthy AI: Closing the gap between development and integration of AI systems in ophthalmic practice. Progress in Retinal and Eye Research. 2022 Sep 1;90:101034.

98. Goodman KW. Ethics in Health Informatics. Yearb Med Inform. 2020 Aug;29(1):26–31.

99. Grote T, Berens P. How competitors become collaborators-Bridging the gap(s) between machine learning algorithms and clinicians. Bioethics. 2022 Feb;36(2):134–42.

100. Guan J. Artificial Intelligence in Healthcare and Medicine: Promises, Ethical Challenges and Governance. Chin Med Sci J. 2019 Jun 30;34(2):76–83.

101. Haines-Delmont A, Chahal G, Bruen AJ, Wall A, Khan CT, Sadashiv R, et al. Testing Suicide Risk Prediction Algorithms Using Phone Measurements With Patients in Acute Mental Health Settings: Feasibility Study. JMIR Mhealth Uhealth. 2020 Jun 26;8(6):e15901.

102. Halm-Pozniak A, Lohmann CH, Zagra L, Braun B, Gordon M, Grimm B. Best practice in digital orthopaedics. EFORT Open Rev. 2023 May 9;8(5):283–90.

103. Hammouda N, Neyra JA. Can Artificial Intelligence Assist in Delivering Continuous Renal Replacement Therapy? Adv Chronic Kidney Dis. 2022 Sep;29(5):439–49.

104. Hane CA, Wasserman M. Designing Equitable Health Care Outreach Programs From Machine Learning Patient Risk Scores. Med Care Res Rev. 2023 Apr;80(2):216–27.

105. Hariharan V, Harland TA, Young C, Sagar A, Gomez MM, Pilitsis JG. Machine Learning in Spinal Cord Stimulation for Chronic Pain. Oper Neurosurg (Hagerstown). 2023 Aug 1;25(2):112–6.

106. Harrer S. Attention is not all you need: the complicated case of ethically using large language models in healthcare and medicine. EBioMedicine. 2023 Apr;90:104512.

107. Harutyunyan R, Jeffries SD, Morse J, Hemmerling TM. Beyond the Echo: The Evolution and Revolution of Ultrasound in Anesthesia. Anesth Analg. 2024 Feb 1;138(2):369–75.

108. Haselager P, Schraffenberger H, Thill S, Fischer S, Lanillos P, van de Groes S, et al. Reflection Machines: Supporting Effective Human Oversight Over Medical Decision Support Systems. Camb Q Healthc Ethics. 2023 Jan 10;1–10.

109. Hazarika I. Artificial intelligence: opportunities and implications for the health workforce. Int Health. 2020 Jul 1;12(4):241–5.

110. Hesse BW, Shneiderman B. eHealth research from the user’s perspective. Am J Prev Med. 2007 May;32(5 Suppl):S97-103.

111. Hine C, Nilforooshan R, Barnaghi P. Ethical considerations in design and implementation of home-based smart care for dementia. Nurs Ethics. 2022 Jun;29(4):1035–46.

112. Holderried F, Stegemann-Philipps C, Herschbach L, Moldt JA, Nevins A, Griewatz J, et al. A Generative Pretrained Transformer (GPT)-Powered Chatbot as a Simulated Patient to Practice History Taking: Prospective, Mixed Methods Study. JMIR Med Educ. 2024 Jan 16;10:e53961.

113. Howard J. Artificial intelligence: Implications for the future of work. Am J Ind Med. 2019 Nov;62(11):917–26.

114. Howard A, Reza N, Aston S, Woods B, Gerada A, Buchan I, et al. Antimicrobial treatment imprecision: an outcome-based model to close the data-to-action loop. Lancet Infect Dis. 2024 Jan;24(1):e47–58.

115. Howard A, Reza N, Aston S, Woods B, Gerada A, Buchan I, et al. Antimicrobial treatment imprecision: an outcome-based model to close the data-to-action loop. Lancet Infect Dis. 2024 Jan;24(1):e47–58.

116. Hua D, Petrina N, Young N, Cho JG, Poon SK. Understanding the factors influencing acceptability of AI in medical imaging domains among healthcare professionals: A scoping review. Artif Intell Med. 2024 Jan;147:102698.

117. Isbanner S, O’Shaughnessy P, Steel D, Wilcock S, Carter S. The Adoption of Artificial Intelligence in Health Care and Social Services in Australia: Findings From a Methodologically Innovative National Survey of Values and Attitudes (the AVA-AI Study). J Med Internet Res. 2022 Aug 22;24(8):e37611.

118. Jansson M, Ohtonen P, Alalääkkölä T, Heikkinen J, Mäkiniemi M, Lahtinen S, et al. Artificial intelligence-enhanced care pathway planning and scheduling system: content validity assessment of required functionalities. BMC Health Services Research. 2022 Dec 12;22(1):1513.

119. Jeyaraman M, Ramasubramanian S, Balaji S, Jeyaraman N, Nallakumarasamy A, Sharma S. ChatGPT in action: Harnessing artificial intelligence potential and addressing ethical challenges in medicine, education, and scientific research. World J Methodol. 2023 Sep 20;13(4):170–8.

120. Joda T, Zitzmann NU. Personalized workflows in reconstructive dentistry-current possibilities and future opportunities. Clin Oral Investig. 2022 Jun;26(6):4283–90.

121. Jones ML. The right to a human in the loop: Political constructions of computer automation and personhood. Soc Stud Sci. 2017 Apr 1;47(2):216–39.

122. Joyce DW, Kormilitzin A, Smith KA, Cipriani A. Explainable artificial intelligence for mental health through transparency and interpretability for understandability. npj Digit Med. 2023 Jan 18;6(1):1–7.

123. Kellmeyer P. Artificial Intelligence in Basic and Clinical Neuroscience: Opportunities and Ethical Challenges. Neuroforum. 2019 Nov 1;25(4):241–50.

124. Kim K, Yang H, Lee J, Lee WG. Metaverse Wearables for Immersive Digital Healthcare: A Review. Adv Sci (Weinh). 2023 Nov;10(31):e2303234.

125. Kirtley OJ, van Mens K, Hoogendoorn M, Kapur N, de Beurs D. Translating promise into practice: a review of machine learning in suicide research and prevention. Lancet Psychiatry. 2022 Mar;9(3):243–52.

126. Koski E, Murphy J. AI in Healthcare. In: Nurses and Midwives in the Digital Age [Internet]. IOS Press; 2021 [cited 2024 Nov 9]. p. 295–9. Available from: https://ebooks.iospress.nl/doi/10.3233/SHTI210726

127. Kremer T, Murray N, Buckley J, Rowan NJ. Use of real-time immersive digital training and educational technologies to improve patient safety during the processing of reusable medical devices: Quo Vadis? Science of The Total Environment. 2023 Nov 20;900:165673.

128. Kretzschmar K, Tyroll H, Pavarini G, Manzini A, Singh I, NeurOx Young People’s Advisory Group. Can Your Phone Be Your Therapist? Young People’s Ethical Perspectives on the Use of Fully Automated Conversational Agents (Chatbots) in Mental Health Support. Biomed Inform Insights. 2019;11:1178222619829083.

129. Kudina O. Regulating AI in Health Care: The Challenges of Informed User Engagement. Hastings Cent Rep. 2021 Sep;51(5):6–7.

130. Lakey JR, Casazza K, Lernhardt W, Mathur EJ, Jenkins I. Machine Learning and Augmented Intelligence Enables Prognosis of Type 2 Diabetes Prior to Clinical Manifestation. Curr Diabetes Rev. 2024 Feb 1;

131. Lalmuanawma S, Hussain J, Chhakchhuak L. Applications of machine learning and artificial intelligence for Covid-19 (SARS-CoV-2) pandemic: A review. Chaos Solitons Fractals. 2020 Oct;139:110059.

132. Lam K, Abràmoff MD, Balibrea JM, Bishop SM, Brady RR, Callcut RA, et al. A Delphi consensus statement for digital surgery. NPJ Digit Med. 2022 Jul 19;5(1):100.

133. Lamanna C. Task-sharing with artificial intelligence: a design hypothesis for an Emergency Unit in sub-Saharan Africa. Pan Afr Med J. 2021;38:387.

134. Lee D, Yoon SN. Application of Artificial Intelligence-Based Technologies in the Healthcare Industry: Opportunities and Challenges. Int J Environ Res Public Health. 2021 Jan 1;18(1):271.

135. Li C, Ye G, Jiang Y, Wang Z, Yu H, Yang M. Artificial Intelligence in battling infectious diseases: A transformative role. J Med Virol. 2024 Jan;96(1):e29355.

136. Li H, Zhang R, Lee YC, Kraut RE, Mohr DC. Systematic review and meta-analysis of AI-based conversational agents for promoting mental health and well-being. npj Digit Med. 2023 Dec 19;6(1):1–14.

137. Li LT, Haley LC, Boyd AK, Bernstam EV. Technical/Algorithm, Stakeholder, and Society (TASS) barriers to the application of artificial intelligence in medicine: A systematic review. J Biomed Inform. 2023 Nov;147:104531.

138. Li W, Fu M, Liu S, Yu H. Revolutionizing Neurosurgery with GPT-4: A Leap Forward or Ethical Conundrum? Ann Biomed Eng. 2023 Oct;51(10):2105–12.

139. Li W, Ge X, Liu S, Xu L, Zhai X, Yu L. Opportunities and challenges of traditional Chinese medicine doctors in the era of artificial intelligence. Front Med (Lausanne). 2023;10:1336175.

140. Li X, Lu Q, Chen P, Gong S, Yu X, He H, et al. Assistance level quantification-based human-robot interaction space reshaping for rehabilitation training. Front Neurorobot. 2023;17:1161007.

141. Lin SY, Mahoney MR, Sinsky CA. Ten Ways Artificial Intelligence Will Transform Primary Care. J Gen Intern Med. 2019 Aug;34(8):1626–30.

142. Liu T, Xiao X. A Framework of AI-Based Approaches to Improving eHealth Literacy and Combating Infodemic. Front Public Health. 2021;9:755808.

143. Liyanage H, Liaw ST, Jonnagaddala J, Schreiber R, Kuziemsky C, Terry AL, et al. Artificial Intelligence in Primary Health Care: Perceptions, Issues, and Challenges. Yearb Med Inform. 2019 Aug;28(1):41–6.

144. Luu VP, Fiorini M, Combes S, Quemeneur E, Bonneville M, Bousquet PJ. Challenges of artificial intelligence in precision oncology: public-private partnerships including national health agencies as an asset to make it happen. Ann Oncol. 2024 Feb;35(2):154–8.

145. Lynn LA. Artificial intelligence systems for complex decision-making in acute care medicine: a review. Patient Safety in Surgery. 2019 Feb 1;13(1):6.

146. Maidhof C, Offermann J, Ziefle M. Eyes on privacy: acceptance of video-based AAL impacted by activities being filmed. Front Public Health. 2023;11:1186944.

147. Malerbi FK, Nakayama LF, Gayle Dychiao R, Zago Ribeiro L, Villanueva C, Celi LA, et al. Digital Education for the Deployment of Artificial Intelligence in Health Care. J Med Internet Res. 2023 Jun 22;25:e43333.

148. Matheny ME, Whicher D, Thadaney Israni S. Artificial Intelligence in Health Care: A Report From the National Academy of Medicine. JAMA. 2020 Feb 11;323(6):509–10.

149. Mathiesen T, Broekman M. Machine Learning and Ethics. Acta Neurochir Suppl. 2022;134:251–6.

150. McHugh K, Pai RK. Deep Learning and Colon Cancer Interpretation: Rise of the Machine. Surg Pathol Clin. 2023 Dec;16(4):651–8.

151. Mese I. Letter to the editor - Leveraging virtual reality-augmented reality technologies to complement artificial intelligence-driven healthcare: the future of patient–doctor consultations. European Journal of Cardiovascular Nursing. 2024 Jan 1;23(1):e9–10.

152. Mese I, Taslicay CA, Sivrioglu AK. Improving radiology workflow using ChatGPT and artificial intelligence. Clin Imaging. 2023 Nov;103:109993.

153. Meskó B. The Impact of Multimodal Large Language Models on Health Care’s Future. Journal of Medical Internet Research. 2023 Nov 2;25:e52865.

154. Metsch JM, Saranti A, Angerschmid A, Pfeifer B, Klemt V, Holzinger A, et al. CLARUS: An interactive explainable AI platform for manual counterfactuals in graph neural networks. J Biomed Inform. 2024 Feb;150:104600.

155. Michelson KN, Klugman CM, Kho AN, Gerke S. Ethical Considerations Related to Using Machine Learning-Based Prediction of Mortality in the Pediatric Intensive Care Unit. J Pediatr. 2022 Aug;247:125–8.

156. Mollura DJ, Culp MP, Pollack E, Battino G, Scheel JR, Mango VL, et al. Artificial Intelligence in Low- and Middle-Income Countries: Innovating Global Health Radiology. Radiology. 2020 Dec;297(3):513–20.

157. Monteith S, Glenn T, Geddes J, Whybrow PC, Achtyes E, Bauer M. Expectations for Artificial Intelligence (AI) in Psychiatry. Curr Psychiatry Rep. 2022 Nov;24(11):709–21.

158. Mun C, Ha H, Lee O, Cheon M. Enhancing AI-CDSS with U-AnoGAN: Tackling data imbalance. Comput Methods Programs Biomed. 2024 Feb;244:107954.

159. Murdoch B. Privacy and artificial intelligence: challenges for protecting health information in a new era. BMC Medical Ethics. 2021 Sep 15;22(1):122.

160. Muthuraj, Singla S. Artificial Intelligence and Machine Learning. Medico Legal Update. 2023 Nov 29;23(5):6–11.

161. Nakagawa K, Moukheiber L, Celi LA, Patel M, Mahmood F, Gondim D, et al. AI in Pathology: What could possibly go wrong? Semin Diagn Pathol. 2023 Mar;40(2):100–8.

162. Nazir A, Wang Z. A comprehensive survey of ChatGPT: Advancements, applications, prospects, and challenges. Meta-Radiology. 2023 Sep 1;1(2):100022.

163. Niel O, Bastard P. Artificial Intelligence in Nephrology: Core Concepts, Clinical Applications, and Perspectives. Am J Kidney Dis. 2019 Dec;74(6):803–10.

164. Ostberg NP, Zafar MA, Elefteriades JA. Machine learning: principles and applications for thoracic surgery. Eur J Cardiothorac Surg. 2021 Jul 30;60(2):213–21.

165. Ostherr K. Artificial Intelligence and Medical Humanities. J Med Humanit. 2022 Jun;43(2):211–32.

166. Ott T, Dabrock P. Transparent human – (non-) transparent technology? The Janus-faced call for transparency in AI-based health care technologies. Frontiers in Genetics. 2022 Aug 22;13:902960.

167. Ou WC, Polat D, Dogan BE. Deep learning in breast radiology: current progress and future directions. Eur Radiol. 2021 Jul;31(7):4872–85.

168. Pagliari C. Digital health and primary care: Past, pandemic and prospects. J Glob Health. 2021 Jul 2;11:01005.

169. Paladugu PS, Ong J, Nelson N, Kamran SA, Waisberg E, Zaman N, et al. Generative Adversarial Networks in Medicine: Important Considerations for this Emerging Innovation in Artificial Intelligence. Ann Biomed Eng. 2023 Oct;51(10):2130–42.

170. Paravastu S, Farhadi F, Hasani N, Theng E, Beegle C, Morris M, et al. Will seeing be believing? Threats to data integrity control in medical imaging. Journal of Nuclear Medicine. 2022 Aug 1;63(supplement 2):2730–2730.

171. Parker W, Jaremko JL, Cicero M, Azar M, El-Emam K, Gray BG, et al. Canadian Association of Radiologists White Paper on De-identification of Medical Imaging: Part 2, Practical Considerations. Can Assoc Radiol J. 2021 Feb;72(1):25–34.

172. Pashkov VM, Harkusha AO, Harkusha YO. Artificial intelligence in medical practice: regulative issues and perspectives. Wiad Lek. 2020;73(12 cz 2):2722–7.

173. Pedersen M, Verspoor K, Jenkinson M, Law M, Abbott DF, Jackson GD. Artificial intelligence for clinical decision support in neurology. Brain Commun. 2020;2(2):fcaa096.

174. Pergolizzi J, LeQuang JAK, Vasiliu-Feltes I, Breve F, Varrassi G. Brave New Healthcare: A Narrative Review of Digital Healthcare in American Medicine. Cureus. 2023 Oct;15(10):e46489.

175. Pierre K, Haneberg AG, Kwak S, Peters KR, Hochhegger B, Sananmuang T, et al. Applications of Artificial Intelligence in the Radiology Roundtrip: Process Streamlining, Workflow Optimization, and Beyond. Semin Roentgenol. 2023 Apr;58(2):158–69.

176. Prabhakaran D, Ajay VS, Tandon N. Strategic Opportunities for Leveraging Low-cost, High-impact Technological Innovations to Promote Cardiovascular Health in India. Ethn Dis. 2019;29(Suppl 1):145–52.

177. Pruinelli L, Michalowski M. Toward an Augmented Nursing-Artificial Intelligence Future. Comput Inform Nurs. 2021 Jun;39(6):296–7.

178. Rabbani N, Bedgood M, Brown C, Steinberg E, Goldstein RL, Carlson JL, et al. A Natural Language Processing Model to Identify Confidential Content in Adolescent Clinical Notes. Appl Clin Inform. 2023 May;14(3):400–7.

179. Rajpurkar P, Chen E, Banerjee O, Topol EJ. AI in health and medicine. Nat Med. 2022 Jan;28(1):31–8.

180. Ramsdale E, Snyder E, Culakova E, Xu H, Dziorny A, Yang S, et al. An introduction to machine learning for clinicians: How can machine learning augment knowledge in geriatric oncology? J Geriatr Oncol. 2021 Nov;12(8):1159–63.

181. Randazzo G, Reitano G, Carletti F, Iafrate M, Betto G, Novara G, et al. Urology: a trip into metaverse. World J Urol. 2023 Oct;41(10):2647–57.

182. Ribeiro MAFR, Smaniotto R, Gebran A, Zamudio JP, Mohseni S, Rodrigues JM da S, et al. The use of POTTER (Predictive Optimal Trees in Emergency Surgery Risk) calculator to predict mortality and complications in patients submitted to Emergency Surgery. Revista do Colégio Brasileiro de Cirurgiões. 2023 Nov 18;50:e20233624.

183. Roberts RHR, Ali SR, Dobbs TD, Whitaker IS. Can Large Language Models Generate Outpatient Clinic Letters at First Consultation That Incorporate Complication Profiles From UK and USA Aesthetic Plastic Surgery Associations? Aesthet Surg J Open Forum. 2024;6:ojad109.

184. Rodler S, Kopliku R, Ulrich D, Kaltenhauser A, Casuscelli J, Eismann L, et al. Patients’ Trust in Artificial Intelligence-based Decision-making for Localized Prostate Cancer: Results from a Prospective Trial. Eur Urol Focus. 2024 Jul;10(4):654–61.

185. Rose C, Barber R, Preiksaitis C, Kim I, Mishra N, Kayser K, et al. A Conference (Missingness in Action) to Address Missingness in Data and AI in Health Care: Qualitative Thematic Analysis. J Med Internet Res. 2023 Nov 23;25:e49314.

186. Rowell C, Sebro R. Who Will Get Paid for Artificial Intelligence in Medicine? Radiol Artif Intell. 2022 Sep;4(5):e220054.

187. Russell RG, Lovett Novak L, Patel M, Garvey KV, Craig KJT, Jackson GP, et al. Competencies for the Use of Artificial Intelligence-Based Tools by Health Care Professionals. Acad Med. 2023 Mar 1;98(3):348–56.

188. Saba L, Biswas M, Kuppili V, Cuadrado Godia E, Suri HS, Edla DR, et al. The present and future of deep learning in radiology. Eur J Radiol. 2019 May;114:14–24.

189. Sangers TE, Wakkee M, Moolenburgh FJ, Nijsten T, Lugtenberg M. Towards successful implementation of artificial intelligence in skin cancer care: a qualitative study exploring the views of dermatologists and general practitioners. Arch Dermatol Res. 2023 Jul;315(5):1187–95.

190. Sezgin E. Artificial intelligence in healthcare: Complementing, not replacing, doctors and healthcare providers. Digit Health. 2023;9:20552076231186520.

191. Sheikh A, Anderson M, Albala S, Casadei B, Franklin BD, Richards M, et al. Health information technology and digital innovation for national learning health and care systems. Lancet Digit Health. 2021 Jun;3(6):e383–96.

192. Shuaib A, Arian H, Shuaib A. The Increasing Role of Artificial Intelligence in Health Care: Will Robots Replace Doctors in the Future? International Journal of General Medicine. 2020 Oct 19;13:891.

193. Siddiqi DA, Miraj F, Raza H, Hussain OA, Munir M, Dharma VK, et al. Development and feasibility testing of an artificially intelligent chatbot to answer immunization-related queries of caregivers in Pakistan: A mixed-methods study. Int J Med Inform. 2024 Jan;181:105288.

194. Sidebottom R, Lyburn I, Brady M, Vinnicombe S. Fair shares: building and benefiting from healthcare AI with mutually beneficial structures and development partnerships. Br J Cancer. 2021 Oct;125(9):1181–4.

195. Sim JZT, Bhanu Prakash KN, Huang WM, Tan CH. Harnessing artificial intelligence in radiology to augment population health. Front Med Technol. 2023;5:1281500.

196. Spear J, Ehrenfeld JM, Miller BJ. Applications of Artificial Intelligence in Health Care Delivery. Journal of Medical Systems. 2023 Nov 17;47(1):121.

197. Sqalli MT, Aslonov B, Gafurov M, Nurmatov S. Humanizing AI in medical training: ethical framework for responsible design. Front Artif Intell. 2023;6:1189914.

198. Steil J, Finas D, Beck S, Manzeschke A, Haux R. Robotic Systems in Operating Theaters: New Forms of Team-Machine Interaction in Health Care. Methods Inf Med. 2019 Jun;58(S 01):e14–25.

199. Stogiannos N, Malik R, Kumar A, Barnes A, Pogose M, Harvey H, et al. Black box no more: a scoping review of AI governance frameworks to guide procurement and adoption of AI in medical imaging and radiotherapy in the UK. Br J Radiol. 2023 Dec;96(1152):20221157.

200. Sujan MA, White S, Habli I, Reynolds N. Stakeholder perceptions of the safety and assurance of artificial intelligence in healthcare. Safety Science. 2022 Nov 1;155:105870.

201. Suppadungsuk S, Thongprayoon C, Miao J, Krisanapan P, Qureshi F, Kashani K, et al. Exploring the Potential of Chatbots in Critical Care Nephrology. Medicines (Basel). 2023 Oct 20;10(10):58.

202. Tan M, Xiao Y, Jing F, Xie Y, Lu S, Xiang M, et al. Evaluating machine learning-enabled and multimodal data-driven exercise prescriptions for mental health: a randomized controlled trial protocol. Front Psychiatry. 2024;15:1352420.

203. Ting DS, Al-Aswad LA. Augmented Intelligence in Ophthalmology: The Six Rights. Asia-Pacific journal of ophthalmology (Philadelphia, Pa). 2021 Jul 13;10(3):231.

204. Trenfield SJ, Awad A, McCoubrey LE, Elbadawi M, Goyanes A, Gaisford S, et al. Advancing pharmacy and healthcare with virtual digital technologies. Adv Drug Deliv Rev. 2022 Mar;182:114098.

205. Tustumi F, Andreollo NA, Aguilar-Nascimento JE de. Future of the language models in healthcare: the role of ChatGPT. Arq Bras Cir Dig. 2023;36:e1727.

206. van der Stigchel B, van den Bosch K, van Diggelen J, Haselager P. Intelligent decision support in medical triage: are people robust to biased advice? J Public Health (Oxf). 2023 Aug 28;45(3):689–96.

207. van der Waa J, Verdult S, Bosch K van den, Diggelen J van, Haije T, Stigchel B van der, et al. Moral Decision Making in Human-Agent Teams: Human Control and the Role of Explanations. Frontiers in Robotics and AI. 2021 May 27;8:640647.

208. Vearrier L, Derse AR, Basford JB, Larkin GL, Moskop JC. Artificial Intelligence in Emergency Medicine: Benefits, Risks, and Recommendations. J Emerg Med. 2022 Apr;62(4):492–9.

209. Vo V, Chen G, Aquino YSJ, Carter SM, Do QN, Woode ME. Multi-stakeholder preferences for the use of artificial intelligence in healthcare: A systematic review and thematic analysis. Soc Sci Med. 2023 Dec;338:116357.

210. Volovici V, Syn NL, Ercole A, Zhao JJ, Liu N. Steps to avoid overuse and misuse of machine learning in clinical research. Nat Med. 2022 Oct;28(10):1996–9.

211. Walker R, Dillard-Wright J, Iradukunda F. Algorithmic bias in artificial intelligence is a problem-And the root issue is power. Nurs Outlook. 2023;71(5):102023.

212. Wang G, Badal A, Jia X, Maltz JS, Mueller K, Myers KJ, et al. Development of metaverse for intelligent healthcare. Nat Mach Intell. 2022 Nov;4(11):922–9.

213. Wang JX, Somani S, Chen JH, Murray S, Sarkar U. Health Equity in Artificial Intelligence and Primary Care Research: Protocol for a Scoping Review. JMIR Res Protoc. 2021 Sep 17;10(9):e27799.

214. Wang L, Chen X, Zhang L, Li L, Huang Y, Sun Y, et al. Artificial intelligence in clinical decision support systems for oncology. International Journal of Medical Sciences. 2023 Jan 1;20(1):79.

215. Watson D, Womack J, Papadakos S. Rise of the Robots: Is Artificial Intelligence a Friend or Foe to Nursing Practice? Crit Care Nurs Q. 2020;43(3):303–11.

216. Wehbe RM, Khan SS, Shah SJ, Ahmad FS. Predicting High-Risk Patients and High-Risk Outcomes in Heart Failure. Heart Fail Clin. 2020 Oct;16(4):387–407.

217. Werutsky G, Barrios CH, Cardona AF, Albergaria A, Valencia A, Ferreira CG, et al. Perspectives on emerging technologies, personalised medicine, and clinical research for cancer control in Latin America and the Caribbean. Lancet Oncol. 2021 Nov;22(11):e488–500.

218. Whiteson HZ, Frishman WH. Artificial Intelligence in the Prevention and Detection of Cardiovascular Disease. Cardiol Rev. 2023 Oct 17;

219. Wong RSY, Ming LC, Raja Ali RA. The Intersection of ChatGPT, Clinical Medicine, and Medical Education. JMIR Med Educ. 2023 Nov 21;9:e47274.

220. Xue VW, Lei P, Cho WC. The potential impact of ChatGPT in clinical and translational medicine. Clinical and Translational Medicine. 2023;13(3):e1216.

221. Yang J. ChatGPTs’ Journey in Medical Revolution: A Potential Panacea or a Hidden Pathogen? Ann Biomed Eng. 2023 Nov;51(11):2356–8.

222. Young AT, Xiong M, Pfau J, Keiser MJ, Wei ML. Artificial Intelligence in Dermatology: A Primer. Journal of Investigative Dermatology. 2020 Aug 1;140(8):1504–12.

223. Youngmann B, Allerhand L, Paltiel O, Yom‐Tov E, Arkadir D. A machine learning algorithm successfully screens for Parkinson’s in web users. Annals of Clinical and Translational Neurology. 2019 Nov 12;6(12):2503.

224. Zack T, Lehman E, Suzgun M, Rodriguez JA, Celi LA, Gichoya J, et al. Assessing the potential of GPT-4 to perpetuate racial and gender biases in health care: a model evaluation study. The Lancet Digital Health. 2024 Jan 1;6(1):e12–22.

225. Zhang X. Exploring Drone Policing Applications for Economic and Bio Commercial Advancements in the Context of Digital Health. Journal of Commercial Bioetechnology. 2023;28(4).

226. Zhang J, Wu J, Zhou XS, Shi F, Shen D. Recent advancements in artificial intelligence for breast cancer: Image augmentation, segmentation, diagnosis, and prognosis approaches. Semin Cancer Biol. 2023 Nov;96:11–25.

227. Zhang K, Khosravi B, Vahdati S, Erickson BJ. FDA Review of Radiologic AI Algorithms: Process and Challenges. Radiology. 2024 Jan;310(1):e230242.

228. Zhang J, Oh YJ, Lange P, Yu Z, Fukuoka Y. Artificial Intelligence Chatbot Behavior Change Model for Designing Artificial Intelligence Chatbots to Promote Physical Activity and a Healthy Diet: Viewpoint. J Med Internet Res. 2020 Sep 30;22(9):e22845.

229. Zhao IY, Ma YX, Yu MWC, Liu J, Dong WN, Pang Q, et al. Ethics, Integrity, and Retributions of Digital Detection Surveillance Systems for Infectious Diseases: Systematic Literature Review. J Med Internet Res. 2021 Oct 20;23(10):e32328.

230. Zhu J, Shi K, Yang C, Niu Y, Zeng Y, Zhang N, et al. Ethical issues of smart home-based elderly care: A scoping review. J Nurs Manag. 2022 Nov;30(8):3686–99.

231. Zidaru T, Morrow EM, Stockley R. Ensuring patient and public involvement in the transition to AI-assisted mental health care: A systematic scoping review and agenda for design justice. Health Expect. 2021 Aug;24(4):1072–124.
